# Comparative genomic, transcriptomic and secretomic profiling of *Penicillium oxalicum* HP7-1 and its cellulase and xylanase hyper-producing mutant EU2106, and identification of two novel regulatory genes of cellulase and xylanase gene expression

**DOI:** 10.1186/s13068-016-0616-9

**Published:** 2016-09-23

**Authors:** Shuai Zhao, Yu-Si Yan, Qi-Peng He, Lin Yang, Xin Yin, Cheng-Xi Li, Li-Chun Mao, Lu-Sheng Liao, Jin-Qun Huang, Shang-Bo Xie, Qing-Dong Nong, Zheng Zhang, Lei Jing, Ya-Ru Xiong, Cheng-Jie Duan, Jun-Liang Liu, Jia-Xun Feng

**Affiliations:** State Key Laboratory for Conservation and Utilization of Subtropical Agro-bioresources, Guangxi Key Laboratory of Subtropical Bioresources Conservation and Utilization, Key Laboratory of Ministry of Education for Microbial and Plant Genetic Engineering, College of Life Science and Technology, Guangxi University, 100 Daxue Road, Nanning, 530004 Guangxi People’s Republic of China

**Keywords:** *Penicillium oxalicum*, Genomics, Transcriptomics, Secretomics, Transcription factor, Cellulase, Xylanase, Regulation

## Abstract

**Background:**

The filamentous fungus *Penicillium oxalicum* is a potential alternative to *Trichoderma reesei* for industrial production of a complete cellulolytic enzyme system for a bio-refinery. Comparative omics approaches can support rational genetic engineering and/or breeding of filamentous fungi with improved cellulase production capacity. In this study, comparative genomic, transcriptomic and secretomic profiling of *P. oxalicum* HP7-1 and its cellulase and xylanase hyper-producing mutant EU2106 were employed to screen for novel regulators of cellulase and xylanase gene expression.

**Results:**

The 30.62 Mb *P*. *oxalicum* HP7-1 genome was sequenced, and 9834 protein-coding genes were annotated. Re-sequencing of the mutant EU2106 genome identified 274 single nucleotide variations and 12 insertion/deletions. Comparative genomic, transcriptomic and secretomic profiling of HP7-1 and EU2106 revealed four candidate regulators of cellulase and xylanase gene expression. Deletion of these candidate genes and measurement of the enzymatic activity of the resultant mutants confirmed the identity of three regulatory genes. *POX02484* and *POX08522*, encoding a putative Zn(II)_2_Cys_6_ DNA-binding domain and forkhead protein, respectively, were found to be novel, while PoxClrB is an ortholog of ClrB, a key transcriptional regulator of cellulolytic enzyme gene expression in filamentous fungi. Δ*POX02484* and Δ*POX08522* mutants exhibited significantly reduced β-glucosidase activity, increased carboxymethylcellulose cellulase and xylanase activities, and altered transcription level of cellulase and xylanase genes compared with the parent strain Δ*PoxKu70*, with Avicel as the sole carbon source.

**Conclusions:**

Two novel genes, *POX02484* and *POX08522*, were found and characterized to regulate the expression of cellulase and xylanase genes in *P. oxalicum*. These findings are important for engineering filamentous fungi to improve cellulase and xylanase production.

**Electronic supplementary material:**

The online version of this article (doi:10.1186/s13068-016-0616-9) contains supplementary material, which is available to authorized users.

## Background

Plant cell walls, which primarily consist of cellulose and hemicellulose, are a potential bio-energy source that is an alternative to unsustainable fossil fuels. However, integrated bio-refineries that process lignocellulosic biomass, including agricultural wastes, woody biomass, forestry residues, and grasses, to generate biomaterials such as second-generation biofuels, still face several challenges, including feedstock production and logistics, the development of energy-efficient technologies (pretreatment, enzyme hydrolysis, and microbial fermentation), and societal acceptance [1]. Of these, insufficient amounts and high costs of cellulolytic enzymes hamper the development of bio-refineries. In the biosphere, most organisms do not directly utilize natural cellulose, except for a few cellulose-utilizing microorganisms that are present in soil and the gut of animals [2]. Therefore, to reduce cellulolytic enzyme costs, manipulation of genes regulating the expression of cellulolytic enzymes in genetically amenable microbes has the potential to enhance enzyme production.

Cellulases are produced commercially by filamentous fungi, mainly *Trichoderma reesei* in recent decades [3]. However, in the native extracellular enzyme system of this organism, secretion of β-glucosidase (BGL, EC 3.2.1.21) is low [4], and cellulase preparations from derivatives of *T*. *reesei* must be supplemented with BGL from other sources to improve the efficiency of cellulose hydrolysis [3]. In contrast, the filamentous fungus *Penicillium oxalicum* secretes a complete cellulase system with a high level of BGL activity [3, 5], and *P*. *oxalicum* might be therefore a potential alternative to *T. reesei* for bioenergy applications [3], although cellulase production must be enhanced if *P*. *oxalicum* is to meet the demands of a commercial cellulase source.

Cellulase is a mixture of endo-glucanase (EG, EC 3.2.1.4), cellobiohydrolase (CBH, EC 3.2.1.91), and BGL, that act synergistically with hemicellulases such as endo-β-1,4-xylanases (EC 3.2.1.8) and β-xylosidases (EC 3.2.1.37), along with other enzymes, to hydrolyse cellulose in the plant cell wall into glucose [6]. The expression of genes that encode these plant cell wall-degrading enzymes (CWDEs) is controlled by a complex regulatory system [7].

Several transcription factors involved in cellulase and hemicellulase gene expression have been identified and characterized, including transcriptional repressors CRE1/CreA in *T*. *reesei* QM9414 [8] and *P*. *oxalicum* 114-2 [9] and Ace1 in *T*. *reesei* ALKO2221 [10], as well as activators Clr1 in *Neurospora crassa* FGSC 2489 [11], Clr2/ClrB in *N. crassa* FGSC 2489 and *P*. *oxalicum* 114-2 [9], Vib1 in *N*. *crassa* FGSC 2489 [12], Bgl2 in *P*. *oxalicum* 114-2 [13], and XlnR in *Aspergillus niger* CBS 120.49 [14] and *P*. *oxalicum* 114-2 [9]. Of these, Clr2/ClrB, which contains a binuclear zinc cluster, is a key transcriptional activator that is essential for inducing the expression of major cellulases, some major hemicellulases, and mannanolytic enzymes in the presence of plant cell walls (*Miscanthus*), cellulose, or cellodextrins. Clr2/ClrB is highly conserved in most filamentous ascomycete fungi such as *N. crassa*, *Aspergillus* sp., *T*. *reesei* and *Penicillium* sp. [9, 10, 15]. Experimental data showed that manipulating Clr2/ClrB expression in filamentous fungi has great potential for enhancing enzyme production for plant cell wall deconstruction [15]. Very recently, the cellulase yield of a genetically engineered *P*. *oxalicum* strain was increased several-fold following induction and/or repression of known transcription factors including ClrB [9, 16]. However, cellulases suitable for use in the industrial-scale bio-refinery of lignocellulosic biomass remain elusive, and the identification and manipulation of additional regulatory genes could be a major step forward in this regard.

In this study, comparative genomic, transcriptomic and secretomic profiling of *P*. *oxalicum* HP7-1 and its cellulase and xylanase hyper-producing mutant EU2106 were employed to screen for candidate regulatory genes that regulate cellulase and/or xylanase gene expression. Knockout of candidate transcription factor genes resulted in mutants that were tested for cellulase and xylanase production, and two novel genes regulating the expression of cellulase and/or xylanase genes were identified.

## Results

### Sequencing of the *P. oxalicum* HP7-1 genome

**Fig. 1:**
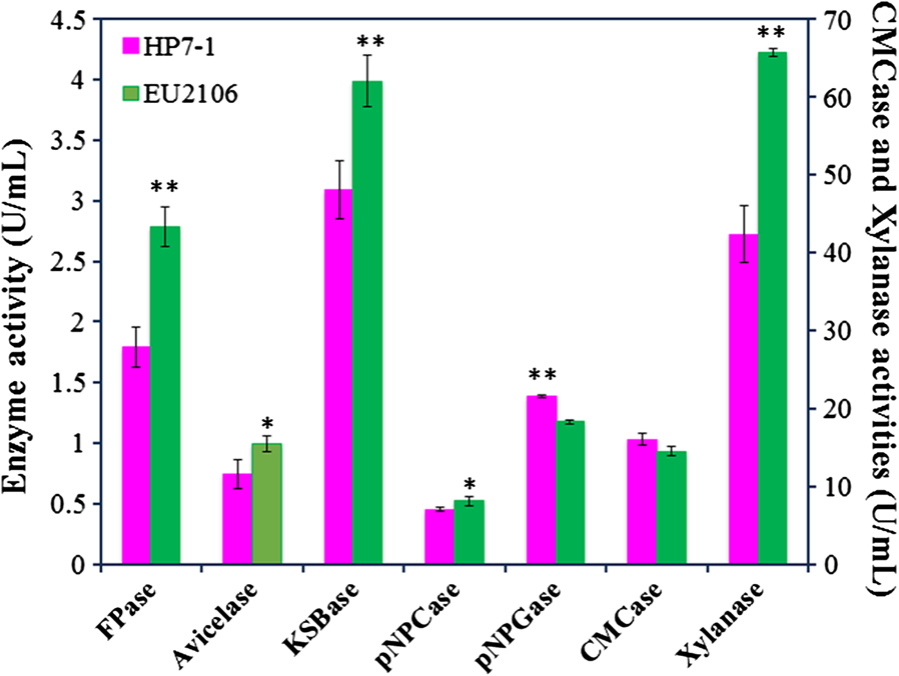
Enzyme activities of FPase, Avicelase, KSBase, CMCase, pNPCase and pNPGase cellulases from *P*. *oxalicum* strains HP7-1 and EU2106. The symbols * and ** indicate significant differences (*P* ≤ 0.05 and *P* ≤ 0.01, respectively) between the two strains (by Student’s *t* test)

*Penicillium oxalicum* strain HP7-1 was isolated from a decayed forest soil system in China [17]. This strain displayed high cellulase activity [5], particularly towards KOH-pretreated sugarcane bagasse (Fig. 1). The cellulase and xylanase hyper-producing mutant EU2106 was derived from HP7-1 after three rounds of γ-irradiation and two rounds of ethyl methanesulfonate/ultraviolet light mutagenesis [18]. To comprehensively characterize cellulolytic enzymes secreted by EU2106, filter paper cellulase (FPase), Avicelase, KOH-pretreated sugarcane bagasse cellulase (KSBase), carboxymethylcellulose cellulase (CMCase), *p*-nitrophenyl-β-cellobioside cellulase (pNPCase), *p*-nitrophenyl-β-glucopyranoside cellulase (pNPGase), and xylanase enzyme activities were measured. The results showed that the secreted FPase activity (2.78 ± 0.16 U/mL) was significantly higher (*P* ≤ 0.01, Student’s *t* test) than that of the wild-type HP7-1 (1.79 ± 0.16 U/mL). Similarly, EU2106 possessed higher Avicelase, KSBase, pNPCase and xylanase activities (*P* ≤ 0.05, Student’s *t* test; Fig. 1), whereas the CMCase and pNPGase activities of strain EU2106 were similar and lower than those of strain HP7-1, respectively.

To further explain the mechanism of enhanced cellulase production in the mutant EU2106, comparative genomics, transcriptomics and secretomics were performed. First, genome sequencing of HP7-1 was conducted using an Illumina HiSeq 2000 system. The genome (accession number JRVD00000000) was assembled using three read libraries, with lengths of 170, 500 and 6000 bp, which were generated by deep sequencing. In total, 30.62 Mbp of nucleotides were generated and covered by 43 scaffolds comprising 263 contigs with 105-fold coverage. The longest contig was 1.18 Mbp, which indicated good assembly continuity. Similar to the genome of *P*. *oxalicum* strain 114-2 reported previously [18], 9834 protein-coding genes were annotated in the genome of *P*. *oxalicum* strain HP7-1 (Table 1) following curation of genes from four databases (*E* value <1e−5), namely, the Kyoto Encyclopedia of Genes and Genomes (KEGG), the National Center for Biotechnology Information non-redundant (NCBI NR), UniProt, and Gene Ontology (GO) databases (Additional file 1: Figure S1). In total, 92.3 % of HP7-1 genes shared more than 90 % identity with those in strain 114-2. A comparative analysis of the general features of the *P*. *oxalicum* HP7-1 and 114-2 genomes is presented in Table 1.

**Table 1:** General genome features of *P*. *oxalicum* strains HP7-1 and 114-2

Genome feature	Value	
	HP7-1	114-2
Size of assembled genome (Mbp)	30.62	30.18
GC content of assembled genome (%)	50.65	50.66
All protein-coding genes	9834	9979
Protein-coding genes ≥60 amino acids	9602	9784
GC content of protein-coding regions (%)	54.44	54.41
Average gene length (bp)	1621	1598
Average number of introns per gene	1.88	1.95
Average intron size (bp)	103	117
Average exon size (bp)	485	464

Carbohydrate-active enzymes (CAZymes) and transcription factors may contribute to the ability of strain HP7-1 to hydrolyse plant cell walls [19]. Notably, 477 genes encoding CAZymes and 484 genes encoding predicted transcription factors were annotated in the genome of HP7-1.

The genome of the cellulase and xylanase hyper-producing mutant EU2106 was re-sequenced (accession number SRA399107) and mapped onto the genome of the wild-type strain HP7-1. In total, 1664 Mbp of clean data were generated by constructing a read library with a length of 500 bp using the Illumina HiSeq 2000 system, which covered approximately 30.59 Mbp of the HP7-1 genome with 99.81 % coverage.

**Fig. 2:**
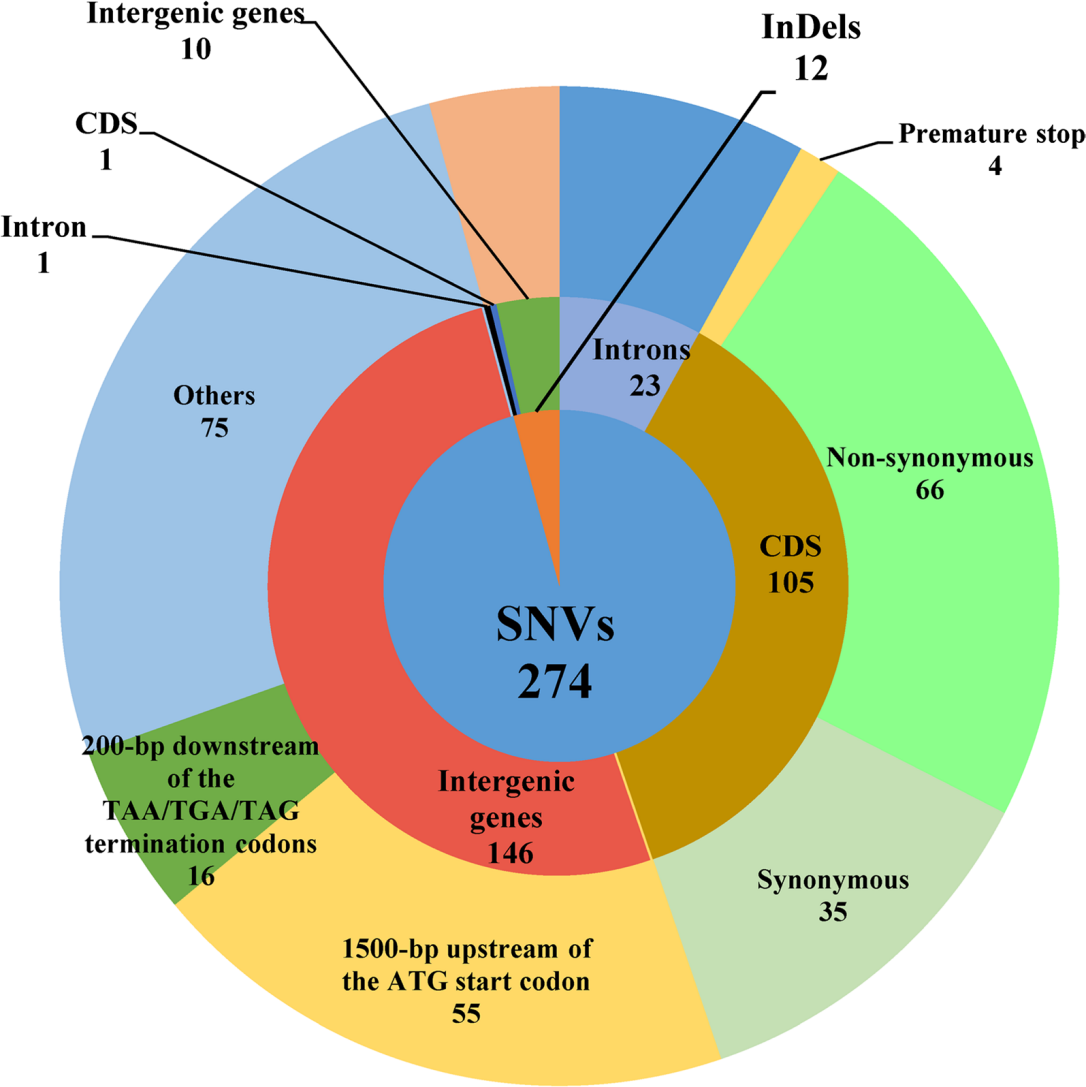
Distribution of single nucleoside variations and InDels in *P*. *oxalicum* strain EU2106. *CDS* coding DNA sequence, *InDels* insertion/deletions

A comparative analysis of the HP7-1 and EU2106 genomes revealed 274 single nucleotide variations (SNVs) and 12 insertion/deletions (InDels) (Additional file 2: Table S1) in EU2106, which was far fewer than reported previously for the *P*. *oxalicum* mutant JU-A10-T compared with its wild-type strain 114-2 [20]. Of the 274 SNVs, 105, 146 and 23 were located in coding DNA sequences (CDSs), intergenic regions, and introns, respectively (Fig. 2). Of the 12 InDels, 11 are deletions and 1 is an insertion (Fig. 2), but only one deletion is located in a CDS, specifically in the *POX01312* gene that encodes a hypothetical protein (according to NCBI BLASTP analysis), resulting in a frameshift mutation.

### Comparison of global gene expression in *P*. *oxalicum* HP7-1 and EU2106

*Penicillium oxalicum* HP7-1 and EU2106 were grown in medium containing wheat bran and Avicel as the carbon source (see the “Methods” section for more details) to investigate global gene expression using transcriptome sequencing on an Illumina HiSeq 2000 system. In total, approximately 25–27 million clean reads of 90 bp in length were generated from each sample [accession number GSE80076 in the gene expression omnibus (GEO) database, or SRA399500 for HP7-1 and SRA399511 for EU2106 in the sequence read archive (SRA) database], representing an average 80-fold coverage for the entire HP7-1 genome. Clean reads were mapped to 8441 predicted protein-coding genes in the HP7-1 genome.

Comparative analysis of the available transcriptome data using the R Bioconductor package NOISeq [21] revealed 347 genes that were differentially expressed (|log_2_ fold change| ≥ 1 and probability ≥ 0.8) in mutant strain EU2106 compared with HP7-1 (grown in medium containing wheat bran and Avicel). Of these, 142 were up-regulated and 205 were down-regulated in EU2106 (Additional file 3: Table S2). After screening the InterPro database [22], approximately 79 % of identified genes were functionally annotated, and the remaining genes were of unknown function and thus could be novel genes involved in cellulase gene expression in *P*. *oxalicum*. Among those of unknown function, 17 genes were up-regulated (1.27 ≤ log_2_ fold change ≤ 5.83), while 56 genes were down-regulated (−12.59 ≤ log_2_ fold change ≤ −1.28) in EU2106, compared with HP7-1 (Additional file 3: Table S2). In addition, 18 genes (4 up- and 14 down-regulated) included predicted secretion signals, which indirectly indicated that other unknown mechanisms were affected by the mutations.

Transporter genes encoding the major facilitator superfamily (MFS), such as the cellodextrin transporter Cdt-C homolog gene *POX06051* [23], together with genes encoding extracellular glycosyl hydrolases, were the most abundant, accounting for 8.1 and 15.9 %, respectively, of all genes that were differentially expressed in EU2106 (Additional file 4: Figure S2A).

Of the up-regulated genes, the transcriptional levels of 10 CAZyme-encoding genes (*POX01796*, *POX01961*, *POX02490*, *POX03641*, *POX05188*, *POX05260*, *POX05570*, *POX05571*, *POX06079* and *POX07971*) in EU2106 were 2.38 to 61.93-folds of that in HP7-1. Interestingly, *POX05570* and *POX05571*, *POX03641* and *POX06079*, and *POX02490* encode EG, BGL and CBH, respectively (Additional file 4: Figure S2B). In contrast, the expression of *POX07535* and *POX06835* that encode an EG and a BGL, respectively [19], and which are abundantly secreted by *P*. *oxalicum*, were significantly down-regulated in EU2106 (log_2_ fold change = −3.85 and −1.59, respectively) compared with HP7-1, and the xylanase-encoding gene *POX05916* was also down-regulated (log_2_ fold change = −2.54) in the mutant strain (Additional file 4: Figure S2C). Of note, a reduction in BGL activity in *P*. *oxalicum* might stimulate cellulase activity by increasing cellobiose concentration, which is an inducer of the cellulase-induction pathway, which could explain the enhanced cellulase activity in the EU2106 mutant strain.

Also of note, eight major MFS members (POX01812, POX01981, POX05155, POX06051, POX06283, POX06915, POX07416, and POX09632) were up-regulated at least two-fold in EU2106 compared with HP7-1. Among them, *POX06051* encodes an ortholog of the cellodextrin transporter Cdt-C (log_2_ fold change = 1.47). This could also contribute to the enhanced cellulase activity of the mutant strain, since over-expression of *cdt*-*c* in *P*. *oxalicum* 114-2 enhanced cellobiohydrolase production by stimulating the expression of cellobiohydrolase-encoding gene *Cel7A*-*2* [23]. Expression of two predicted Zn(II)_2_Cys_6_ domain-containing transcription factors POX02484 and POX03873 was down-regulated approximately 3.5-fold in EU2106 compared with HP7-1, whereas *POX07938,* that also encodes a protein with a Zn(II)_2_Cys_6_ domain, was up-regulated 14.8-fold (Additional file 3: Table S2).

### Comparative analysis of the secretomes of *P*. *oxalicum* HP7-1 and EU2106

A total of 240 proteins were identified in the secretomes of HP7-1 and EU2106 (accession number PXD004046) using isobaric tags for relative and absolute protein quantitation, which increases our understanding of the complete enzyme set secreted by *P*. *oxalicum*. According to KEGG annotation, these proteins are mainly involved in carbohydrate metabolism (Additional file 5: Figure S3). Approximately 55 % of these proteins lack a predicted secretion signal (Additional file 6: Table S3), which is comparable to the secretome of *T*. *asperellum* S4F8 and *T*. *reesei* Rut C30 [24]. This could indicate possible non-classic secretory mechanisms or protein leakage due to occasional cell autolysis or autophagy.

Many of the 240 proteins involved in the hydrolysis of plant cell wall polysaccharides were elevated in EU2106 compared with HP7-1. Of particular note, three CBHs (POX02490, POX04786, and POX05587), eight EGs (POX01166, POX01896, POX02740, POX04137, POX05571, POX06147, POX06983, and POX07535), two BGLs (POX003641 and POX06079), and five xylanases (POX00063, POX05916, POX06601, POX08484, and POX08990) were up-regulated (Additional file 6: Table S3), consistent with some of the transcriptome data described above.

### Screening of candidate regulators of cellulase and/or xylanase gene expression

To identify novel genes regulating cellulase and/or xylanase gene expression, all 274 SNVs in EU2106 were analyzed in detail. Non-synonymous SNVs in CDSs that result in amino acid substitutions in the translated protein could potentially affect protein function [25], while SNVs that occur in the promoters and terminators of protein-coding genes could influence their transcriptional levels. Among the 70 non-synonymous SNVs, none were located in sequences encoding CWDEs, except for *POX00008*, which encodes an exo-α-l-1,5-arabinanase. The four SNVs that occurred in *POX02087*, *POX03561*, *POX03683*, and *POX07858* would halt translation and possibly affect the functions of their encoded proteins, which are putatively associated with DNA repair and protein modification. Notably, three SNVs were located in the CDSs of *PoxClrB* (*POX01960*), *POX03199* and *POX08522*, that encode two predicted transcription factors and a homolog of the ClrB transcription factor that positively regulates cellulase and/or hemicellulase gene expression in *P. oxalicum* 114-2 in the presence of various carbon sources [9].

In contrast, 53 and 10 SNVs were found within 1500 and 200 bp of ATG start codons and termination codons, respectively. KEGG annotation indicated that most genes containing SNVs were associated with genetic information processing and metabolism (Additional file 2: Table S1).

Transcriptional of all genes containing SNVs in both HP7-1 and the cellulase and xylanase hyper-producing EU2106 mutant was measured in media containing wheat bran and Avicel as the carbon source. In total, eight genes containing SNVs were altered more than twofold in EU2106 compared with HP7-1 (five up- and three down-regulated, respectively; Additional file 3: Table S2). Of these eight genes, five (*POX00301*, *POX06751*, *POX06820*, *POX07291*, and *POX09827*) are particularly relevant since the SNVs are located in CDS, promoter, or terminator regions of open reading frames.

Together, these results identified 11 potentially important genes, including five (*POX00301*, *POX06751*, *POX06820*, *POX07291*, and *POX09827*) in which SNVs were located in CDSs or within 1500 or 200 bp of ATG start codons or termination codons, respectively, and that were differentially expressed (|log_2_ fold change| ≥ 1) in the mutant strain. The other six potentially important genes comprised three transcription factor-encoding genes (*PoxClrB*, *POX03199*, and *POX08522*) with SNVs in their CDS regions, and three genes (*POX02484*, *POX03873* and *POX07938*) with significantly altered expression (|log_2_ fold change| ≥ 1 and probability ≥ 0.8) in EU2106. Detailed analysis using the NCBI protein basic local alignment search tool (BLASTP) indicated that four proteins (PoxClrB, POX02484, POX07291 and POX08522) were worthy candidates for further characterization of their regulatory functions in cellulase and xylanase gene expression (Additional file 7: Table S4).

### Construction and characterization of deletion mutants for four candidate cellulase and xylanase regulators

To comprehensively investigate gene function in filamentous fungi, a highly efficient gene targeting system that allows rapid genetic manipulation is needed, and can be achieved by deleting genes involved in the non-homologous end-joining pathway such as *Ku70* and *Ku80* [26]. In the present study, the double-stranded break repair complex subunit-encoding gene *POX01582*, which is homologous to *Ku70*, was identified and subsequently knocked out in *P*. *oxalicum* strain HP7-1 as described previously [26]. The resultant mutant Δ*PoxKu70* displayed no significant defects in phenotypes such as vegetative growth and conidiation or cellulase/xylanase activities, and sensitivity to hygromycin B, G418, and ultraviolet light was comparable with HP7-1 (Additional file 8: Figure S4), suggesting Δ*PoxKu70* was suitable for the construction of deletion mutants.

The four gene candidates (*PoxClrB*, *POX02484*, *POX07291*, and *POX08522*) were individually knocked out in the Δ*PoxKu70* strain by homologous recombination, and their roles in regulating cellulase and xylanase activities were investigated by measuring FPase, CMCase, pNPCase, pNPGase, and xylanase enzyme activity. All deletion mutants were verified by PCR with gene-specific primers and by Southern hybridization analysis (Additional file 9: Figure S5). A crude enzyme solution from each strain was collected after 6 days of cultivation in medium containing Avicel as the sole carbon source. As expected, all enzyme activities were significantly decreased (by 62.18 ± 4.60 to 94.51 ± 1.36 %; *P* ≤ 0.01, Student’s *t* test) in the Δ*PoxClrB* mutant, compared with the Δ*PoxKu70* parent strain, except for pNPGase activity, which was increased by 134.55 ± 26.39 %. This result is consistent with previously findings [9], and confirmed that PoxClrB is a global transcription factor that positively regulates cellulolytic enzyme production. Similarly, FPase, CMCase, pNPCase, and pNPGase enzyme activities were decreased by 36.42 ± 5.58, 17.74 ± 2.96, 35.91 ± 6.61 and 76.64 ± 5.23 %, respectively, (*P* ≤ 0.01, Student’s *t* test) in the Δ*POX02484* mutant (Additional file 10: Figure S6). The Δ*POX08522* mutant lost 85.85 ± 1.22 % (*P* ≤ 0.01, Student’s *t* test) of its pNPGase activity, whereas xylanase activity was increased by 17.38 ± 1.93 % (*P* ≤ 0.01, Student’s *t* test) in this mutant strain. In contrast, there were no significant differences in enzyme activity between the Δ*POX07291* mutant and Δ*PoxKu70* (data not shown). In addition, the yield of secreted protein was decreased by 33.50 ± 4.95 and 62.18 ± 4.60 % respectively in the Δ*POX02484* and Δ*PoxClrB* mutants (*P* ≤ 0.01, Student’s *t* test), compared with Δ*PoxKu70* (Additional file 10: Figure S6).

Together, these preliminary results indicated that POX02484 and POX08522 are novel transcriptional factors that are potentially involved in cellulase and/or xylanase production in *P*. *oxalicum*. Δ*POX02484* and Δ*POX08522* mutants, and the Δ*PoxClrB* strain in which the known regulator PoxClrB was deleted, were therefore selected for further investigation.

### Cell growth and enzyme production in Δ*PoxClrB*, Δ*POX02484* and Δ*POX08522* under Avicel induction

**Fig. 3:**
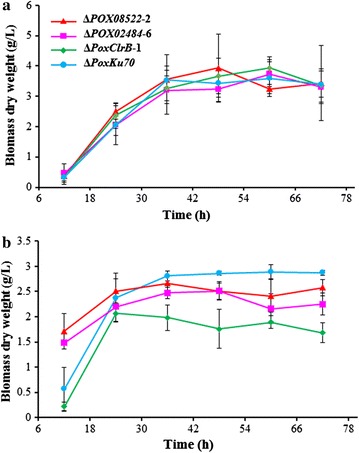
Growth profiles of *P*. *oxalicum* grown on glucose (**a**) and Avicel (**b**). The biomass dry weight per litre was directly measured gravimetrically for glucose or by calculating from the intracellular protein content for Avicel on the basis of 0.28 g intracellular proteins per gram dry biomass. Data are presented as the mean of three biological replicates

To determine whether the candidate regulators were involved in cellulase and xylanase production under Avicel induction conditions, we investigated the growth of Δ*PoxClrB*, Δ*POX02484* and Δ*POX08522* mutants, along with the Δ*PoxKu70* parent strain, in glucose and Avicel. As shown in Fig. 3a, the growth of all three mutants showed no significant differences from the parent strain in medium containing glucose as the sole carbon source, suggesting these genes are not involved in fungal basic metabolism. In contrast, Δ*POX02484* and Δ*POX08522* grew significantly faster before 12 h with Avicel as the sole carbon source than did mutant Δ*PoxClrB* or the Δ*PoxKu70* parent strain. Furthermore, after 24 h of cultivation, the growth of Δ*PoxKu70* was fastest, followed by Δ*POX08522* and Δ*POX02484*, and the Δ*PoxClrB* grew at slowest rate (Fig. 3b).

**Fig. 4:**
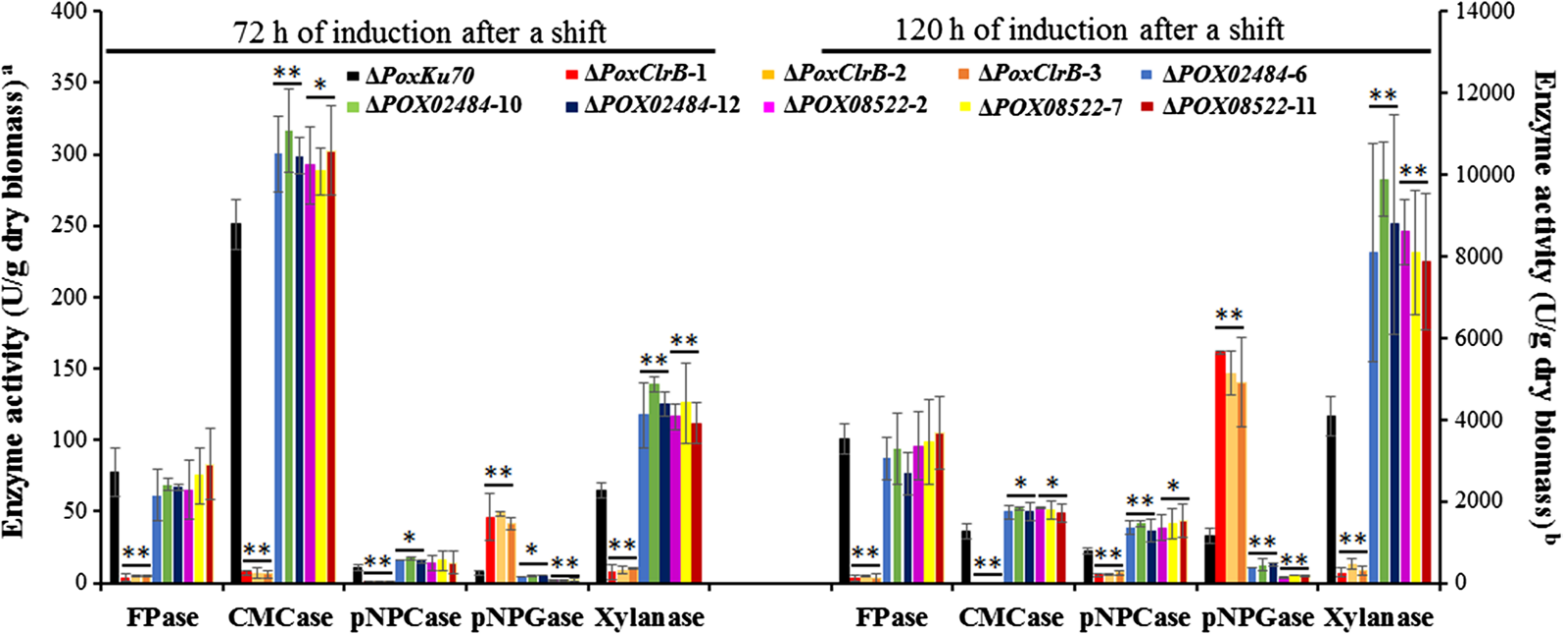
Activities of crude enzymes from *PoxClrB*, *POX02484* and *POX08522* deletion mutants following a shift from glucose to Avicel. Crude enzymes were produced by fungal strains grown in 2.0 % Avicel as the sole carbon source. Enzymatic activity was measured at 3 and 5 days after the shift. The symbols * and ** indicate significant differences (*P* ≤ 0.05 and *P* ≤ 0.01, respectively) between the candidate mutants and the parent Δ*PoxKu70* strain, as assessed by Student’s *t* test. ^a^ FPase, pNPCase, pNPGase activities and CMCase activity from 72 h of induction; ^b^ Xylanase activity and CMCase activity from 120 h of induction

Enzyme production was then tested after 3 and 5 days of induction by Avicel. The activity of all tested enzymes was reduced to below 14 % that of Δ*PoxKu70* in mutant Δ*PoxClrB* after 3 days, except for pNPGase activity, which was 526 % higher than that of Δ*PoxKu70* (*P* ≤ 0.01, Student’s *t* test; Fig. 4), consistent with enzyme production in Avicel after 6 days as described above. Interestingly, in the Δ*POX08522* mutant, only pNPGase activity was significantly decreased, by 71.82 ± 3.62 % (*P* ≤ 0.01, Student’s *t* test), whereas CMCase and xylanase activities were increased by 17.31 ± 2.98 % (*P* ≤ 0.05, Student’s *t* test) and 80.76 ± 10.88 % (*P* ≤ 0.01, Student’s *t* test), compared with Δ*PoxKu70*. Similar to the Δ*POX08522* mutant, Δ*POX02484* also showed significantly decreased pNPGase activity, while CMCase, pNPCase and xylanase activities were increased to varying extents, and xylanase activity was particularly increased, by 94.63 ± 16.83 % (Fig. 4).

After 5 days of Avicel induction, the trends in enzyme activities in all three mutant strains were in agreement with those observed after 3 days, although the variation was larger (Fig. 4).

### POX02484 and POX08522 regulate the expression of cellulase and xylanase genes

**Fig. 5:**
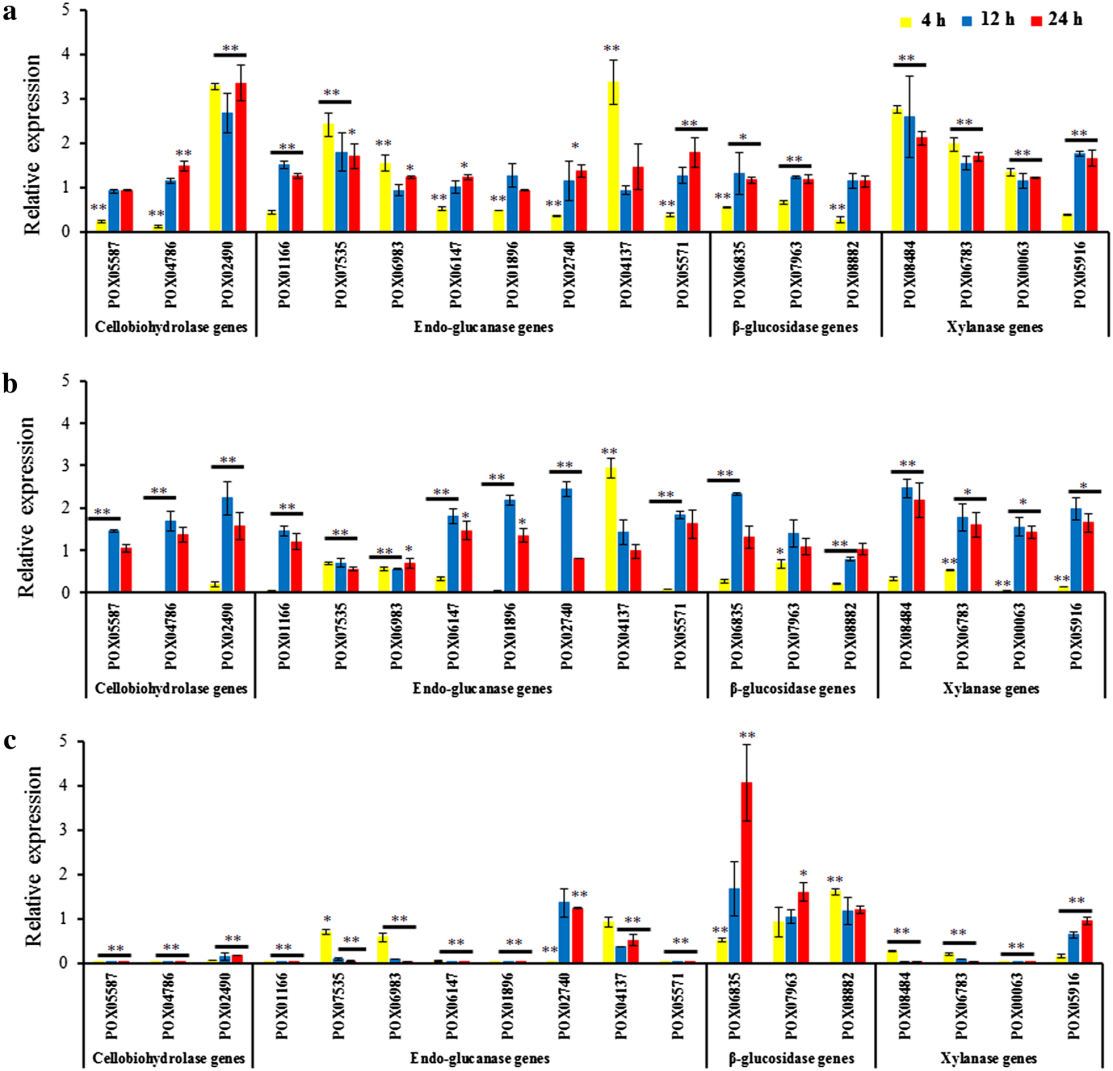
Regulation of gene expression by *PoxClrB*, *POX02484*, and *POX08522*. Under induction by the same carbon source, the expression levels of genes encoding three cellobiohydrolases, eight endo-glucanases, three β-glucosidases and four xylanases were determined in the Δ*POX02484*-6 (**a**), Δ*POX08522*-2 (**b**) and Δ*PoxClrB*-1 (**c**) mutants at three different time points after the shift (4, 12 and 24 h) by qRT-PCR. Expression levels were normalized against the Δ*PoxKu70* parent strain. The symbols * and ** indicate significant differences (*P* ≤ 0.05 and *P* ≤ 0.01, respectively) between the tested samples and the Δ*PoxKu70* strain, as assessed by Student’s *t* test

To directly elucidate transcriptional regulatory roles of these candidate transcription factors, real-time quantitative reverse transcription-PCR (qRT-PCR) was used to measure the expression of their target genes at 4, 12 and 24 h after a shift from glucose to Avicel. Target genes were chosen based on the secretome data described above, and included three cellobiohydrolase genes (*cbh*), eight endo-glucanase genes (*eg*), three β-glucosidase genes (*bgl*), and four xylanase genes (*xyn*) (Fig. 5). The results showed that in the Δ*POX02484* mutant, expression of a number of cellulase and xylanase genes was down-regulated at 4 h. Specifically, two major *cbh* genes (*POX05587*/*Cel7A*-*2* and *POX04786*/*Cel6A*), five *eg* genes *(POX06147*/*Cel5A*, *POX01166*/*Cel5B*, *POX01896/Cel5C*, *POX05571*/*Cel7B*, and *POX02740*, *P* ≤ 0.01, Student’s *t* test), three *bgl* genes (*POX07963*, *POX08882*, *P* ≤ 0.01, Student’s *t* test; *POX06835/Bgl3A*, *P* ≤ 0.05, Student’s *t* test), and one *xyn* gene (*POX05916*, *P* ≤ 0.01, Student’s *t* test) were significantly down-regulated by 34.30–87.29 %, compared with Δ*PoxKu70*. Conversely, *cbh* gene *POX02490/Cel7A*-*1* (*P* ≤ 0.01, Student’s *t* test), three *eg* genes (*POX07535/Cel12A*, *POX04137*, *P* ≤ 0.01, Student’s *t* test; *POX06983*, *P* ≤ 0.05, Student’s *t* test), and three *xyn* genes (*POX00063/xyn10A*, *POX06783/xyn11A* and *POX08484/xyn11B*, *P* ≤ 0.01, Student’s *t* test) were significantly up-regulated from 34.01 to 236.31 % in the Δ*POX02484* mutant (Fig. 5a). Notably, the expression levels of cellulase and xylanase genes at 12 h were significantly different from those at 4 h. For example, two *cbh* genes (*POX05587*/*Cel7A*-*2* and *POX04786*/*Cel6A*), four *eg* genes *(POX06147*/*Cel5A*, *POX01896/Cel5C*, *POX07535/Cel12A* and *POX04137*), and *bgl* gene *POX08882* in Δ*POX02484* were comparable to Δ*PoxKu70*, whereas three *eg* genes (*POX01166*/*Cel5B*, *POX05571*/*Cel7B*, *P* ≤ 0.01, Student’s *t* test) and *POX02740* (*P* ≤ 0.05, Student’s *t* test), two *bgl* genes (*POX06835/Bgl3A*, *P* ≤ 0.05, Student’s *t* test; *POX07963*, *P* ≤ 0.01, Student’s *t* test), and *xyn* gene *POX05916* (*P* ≤ 0.01, Student’s *t* test) were significantly up-regulated compared with Δ*PoxKu70*. At 24 h, most cellulase and xylanase genes were expressed at levels comparable with those measured at 12 h (Fig. 5a).

Like POX02484, another regulator POX08522 also positively regulated all tested cellulase and xylanase genes at 4 h (by 31.31–98.43 %), except for *eg* gene *POX04137* that was increased by 193.67 ± 29.75 % (*P* ≤ 0.01, Student’s *t* test) compared with Δ*PoxKu70* at 4 h (Fig. 5b). In contrast, most cellulase and xylanase genes were significantly up-regulated at 12 h, including three *cbh* genes (*POX05587*/*Cel7A*-*2*, *POX02490*/*Cel7A*-*1* and *POX04786*/*Cel6A*), five *eg* genes (*POX06147*/*Cel5A*, *POX01166*/*Cel5B*, *POX01896/Cel5C*, *POX05571*/*Cel7B*, and *POX02740)*, one *bgl* gene (*POX06835/Bgl3A*), and four *xyn* genes (*POX00063/Xyn10A*, *POX06783/Xyn11A*, *POX08484/Xyn11B* and *POX05916*; Fig. 5b) compared with Δ*PoxKu70* at 12 h. Again, expression levels of most of cellulase and xylanase genes at 24 h were comparable to those observed at 12 h (Fig. 5b).

Similar to previously reported data [9], transcription of all tested *cbh*, *eg* and *xyn* genes at 4, 12 and 24 h was down-regulated in the Δ*PoxClrB* mutant compared with Δ*PoxKu70* (*P* ≤ 0.01, Student’s *t* test; Fig. 5c), except for *eg* genes *POX02740* at 12 and 24 h and *POX04137* at 4 h, and *xyn* gene *POX05916* at 24 h. Interestingly, transcription of all three *bgl* genes varied significantly with induction time. *POX06835*/*Bgl3A* expression in the Δ*PoxClrB* mutant was decreased by 48.11 ± 3.59 % at 4 h, but increased to levels comparable with Δ*PoxKu70* at 12 h, and continued to increase to levels that were 406.41 ± 86.43 % higher than those of the Δ*PoxKu70* strain at 24 h. In comparison, expression levels of *POX07963* and *POX08882* in the Δ*PoxClrB* mutant were 159.38 ± 20.87 and 161.45 ± 67.32 % higher than in Δ*PoxKu70* at 24 and 4 h, respectively (Fig. 5c).

### Sequence analysis of POX02484 and POX08522

The CDSs of *POX02484* and *POX08522* (accession numbers KU597419 and KU597417) were found to be 2067 and 2151 bp, respectively, encoding proteins of 688 and 716 aa, respectively. The POX02484 and POX08522 protein sequences were used as queries in a BLASTP search of the NCBI database (http://www.ncbi.nlm.nih.gov/), and phylogenetically analyzed.

**Fig. 6:**
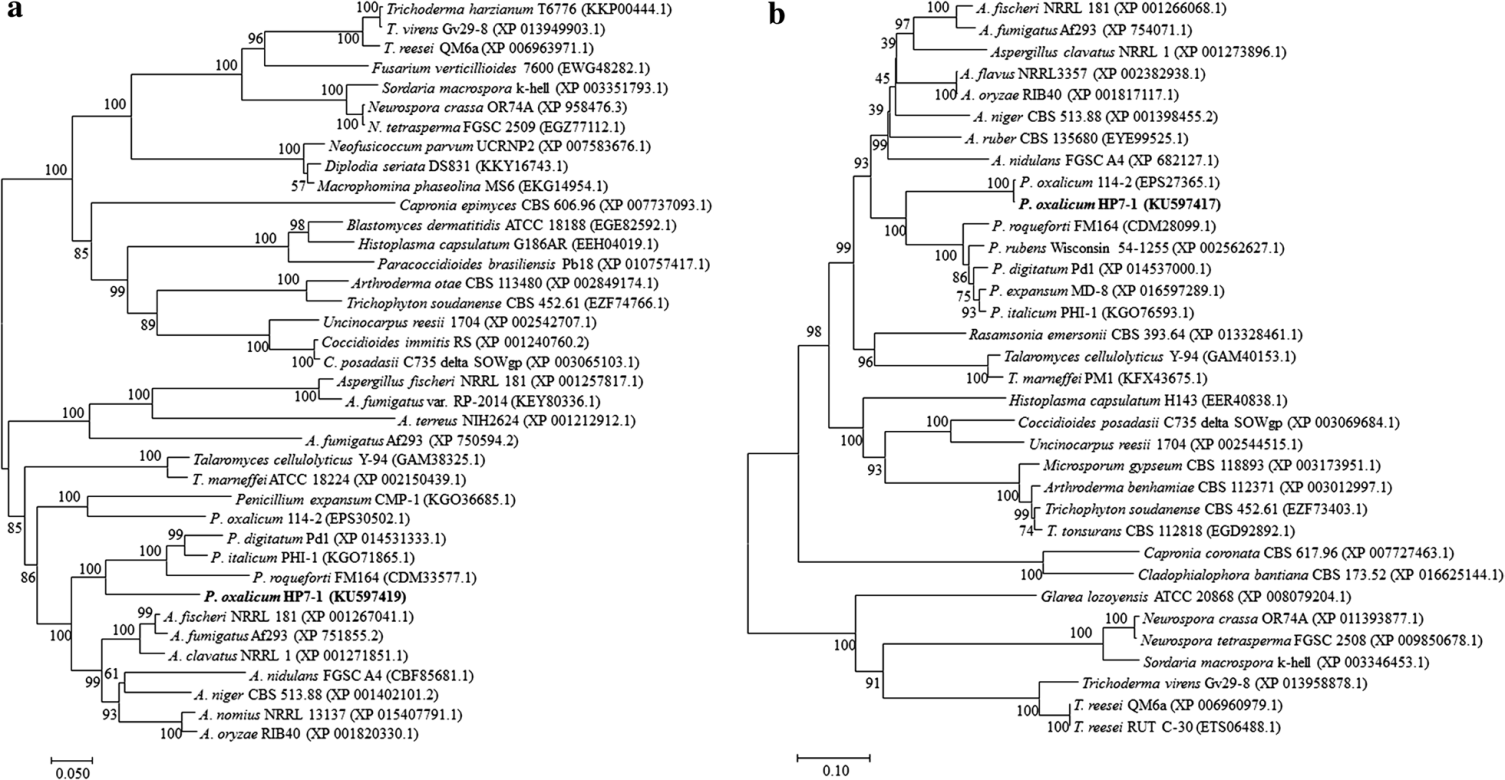
Unrooted phylogenetic analysis of POX02484 (**a**) and POX08522 (**b**) and putative homologs. The dendrogram was constructed using MEGA 7.0 software with the neighbour-joining method and Poisson model. Bootstrap values shown at the nodes are derived from 1000 replicates, and branch lengths corresponding to the divergence of sequences are indicated by the *scale bar*

POX02484 was found to contain a GAL4-like Zn_2_Cys_6_ binuclear cluster DNA-binding domain (cd00067, *E* value = 5.80e−11) and a conserved fungi-specific transcription factor domain (pfam11951, 1.14e−77). POX02484 shares 69, 60, 45 and 43 % identity with the C6 transcription factor AFUA_4G09710 from *A*. *fumigatus* AF293 (XP_751855.2), PDE_05453 from *P*. *oxalicum* 114-2 (EPS30502.1), transcriptional regulatory protein NCU07392 from *N*. *crassa* (EAA29240.3), and TRIREDRAFT_76590 from *T. reesei* QM6a (XP_006963971.1), respectively. Phylogenetic tree analysis clustered POX02484 homologs in *Penicillium* with homologs in *Aspergillus* (Fig. 6a).

POX08522 contains a forkhead box (cd00059, *E* value 1.15e−44) between aa 195 to 271. This protein shares 99 % identity with PDE_02308 (EPS27365.1) from *P*. *oxalicum* 114-2, and 69 and 61 % identity with Pc20g00650 from *P. rubens* Wisconsin 54-1255 (XP_002562627.1) and AOR_1_1186184 of *A*. *oryzae* RIB40 (XP_001817117), respectively, and a somewhat lower identity (37.35 and 43 %) with NCU06173 of *N*. *crassa* (ESA43188.1) and M419DRAFT_67752 of *T*. *reesei* Rut C-30 (ETS06488.1). As with POX02484, phylogenetic tree analysis clustered POX08522 homologs in *Penicillium* with their counterparts in *Aspergillus* (Fig. 6b).

## Discussion

In this study, we performed a comparative omics analysis combining genomics, transcriptomics and secretomics of *P*. *oxalicum* strain HP7-1 and its cellulase and xylanase hyper-producing mutant EU2106. This microorganism has potential for application in the bio-refinery of lignocellulosic biomass [18, 27, 28]. Unsurprisingly, there were no mutations in the catalytic residues of the major cellulase and hemicellulase enzymes in EU2016. However, expression of these enzymes was significantly up-regulated in the mutant strain, as previously observed in *P*. *oxalicum* JU-A10-T and *T*. *reesei* cellulase over-producing mutants [20, 29], although fewer mutations in genes encoding transcription factors were found in *P*. *oxalicum* EU2106 than in JU-A10-T [20]. Functional studies on *P*. *oxalicum* in this work demonstrated that some of the mutated transcription factors were involved in cellulase gene expression. In particular, the master transcription factor PoxClrB included a SNV in its CDS, which changed S725 to N, and transcription factor POX08522 underwent a SNV in its CDS that changed H612 to Y. However, comparative transcriptomics showed that these two transcription factors were expressed equally in the EU2106 mutant and wild-type strain HP7-1. Therefore, whether these alterations affect protein function requires further investigation.

In addition, the transcription factor POX02484 was found to be down-regulated (log_2_ fold change = −1.82) in mutant EU2106, which resulted in an increase in the expression of major cellulase and xylanase genes at 12 and 24 h after carbon source induction, which could partially explain the enhanced cellulase activity in the mutant strain. However, the detailed molecular mechanism underlying the cellulase hyper-producing activity of EU2106 remains unknown.

Phylogenetic analysis indicated that homologs of POX02484 and POX08522 exist in a wide variety of filamentous fungi, including *Neosartorya* spp., *Penicillium* spp., *Aspergillus* spp. and *N*. *crassa*. Interestingly, POX02484 contains a GAL4-like binuclear Zn_2_Cys_6_ cluster DNA-binding domain, which is found in many transcription factors and plays a role in transcriptional regulation in a wide range of processes, including carbon and nitrogen metabolism, amino acid and vitamin synthesis, and morphogenesis, in combination with a regulation domain such as Fungal_trans_2 (pfam11951) and Fungal_TF_MHR (cd12148) [28]. POX08522 harbours a forkhead domain, which can bind nucleosomal DNA and participate in a wide variety of cellular processes, including cell cycle regulation, control of cell death, pre-mRNA processing, and morphogenesis, in both yeast and filamentous fungi, as well as mammals [30].

Multiple alignment of homologs revealed that POX02484 shares 45–69 % identity with Pro1 in *N*. *crassa* OR74A (NCU07392; XP_958476.3) and *Sordaria macrospora* K-hell (SMAC_00338; XP_003351793.1), and NosA (number of sexual spores) in *A*. *fumigatus* AF293 (AFUA_4G09710; XP_751855.2) and *A. nidulans* FGSC A4 (ANIA_01848; CBF85681.1). Transcription factor Pro1 from *S*. *macrospora* is a developmental regulator that is required for fruiting body development and preventing the transition of primordia into mature fruiting bodies [31]. NosA from *Aspergillus* spp., shares ~45 % identity with *S*. *macrospora* Pro1, and is involved in the control of sexual development. Deletion of *NosA* impaired the development of sexual primordia, drastically reduced sexual spore production during self-mating, and increased vegetative growth and germination rates by regulating the expression of several genes associated with sexual development, including the glucose transporter *hxtA* and the catalase peroxidase *cpeA* [32, 33]. Furthermore, NosA function is conserved in other *Aspergillus* species, and when constitutively expressed during the life cycle of *A*. *nidulans*, it was up-regulated during late asexual development following carbon starvation [33].

In addition, multiple alignment of homologs also revealed that POX08522 shares 49 % identity with transcription factor Sep1 in *Schizosaccharomyces pombe* 972 h (SPBC4C3.12; NP_596301.1). Sep1 as a transcription activator that controls mitosis in *S*. *pombe* through the binding and activation of a small subset of mitotic genes involved in septation [34].

Although there are mechanistic differences between Pro1/NosA and Sep1, deletion of either results in an increase in vegetative growth [32, 35]. Here, deletion of POX02484 and POX08522 in *P*. *oxalicum* HP7-1 increased growth during the early stages of development under carbon starvation, but growth was slightly slower than the wide-type during the later stages. Notably, vegetative growth was similar between mutants Δ*POX02484* and Δ*POX08522* and the parent Δ*PoxKu70* in the present study when grown on glucose, suggesting POX02484 and POX08522 act through a mechanism that is different from their homologs in other species.

This is the first study to report the involvement of POX02484 and POX08522 or their homologs in cellulase and xylanase production under cellulosic induction, and the first to conclude that these proteins act as regulators of cellulase and xylanase gene expression in filamentous fungi. Enzymatic activity assays under insoluble cellulose induction indicated that both POX02484 and POX08522 regulated BGL activity, as did PoxClrB. BGL activity is generally considered rate-limiting and can act as a bottleneck that limits the efficient enzymatic degradation of cellulose [4]. Deletion of either POX02484 or POX08522 increased the activity of all cellulases tested except for pNPGase, which is a measure of the proportion of BGL activity responsible for the degradation of cellobiose, a major inducer of cellulase gene expression. In the presence of a strong inducer, cellulase gene expression is up-regulated resulting in very high levels of cellulase activity. This is reminiscent of intracellular β-glucosidase Bgl2 in *P*. *oxalicum* 114-2, since production of all extracellular cellulolytic enzymes including CMCase, pNPCase, pNPGase and xylanases were up-regulated in the Δ*Bgl2* strain when grown on insoluble cellulose [13]. Conversely, all cellulase activities in the Δ*PoxClrB* mutant were reduced except for pNPGase, indicating that a low level of intracellular cellobiose could not stimulate cellulase gene expression in this mutant. To our knowledge, this is the first report on the involvement of PoxClrB on BGL activity. As expected, all three regulators regulated BGL gene expression under induction conditions, to varying degrees. Cellobiose is reported to act as an adaptive BGL inducer rather than a cellulase inducer [36], and may therefore perform a more specific regulatory role in *P*. *oxalicum* than the other transcription factors identified in this study.

In the comparative secretome analysis, more proteins were found to be associated with secondary metabolism pathways in the secretome of strain HP7-1, particularly carbohydrate metabolism, amino acid metabolism, xenobiotics biodegradation and metabolism, and energy metabolism, compared with the 105 proteins in the secretomes of both *P*. *oxalicum* 114-2 and JA-10-T [20]. Specifically, 42 additional CAZymes and one extra MFS were detected, including EGs POX04137 and POX06983, polysaccharide monooxygenases POX05968 and POX08897, expansin-like protein POX01524, and cellodextrin transporter POX06051, indicating a complex synergism between enzymes involved in plant cell wall degradation. Surprisingly, POX03641 and POX06079, previously defined as intracellular BGLs [13], were found in the secretomes of *P*. *oxalicum* strains HP7-1 and EU2106, which may merit further investigation.

In *P*. *oxalicum* strain 114-2, Li et al. [9] recently reported that 20 transcription factors were involved in the regulation of cellulase and/or hemicellulase gene expression by constructing a single-gene disruptive mutant library of its putative transcription factors. Hydrolysis zones on plates containing cellulose produced by the constructed mutants were used for screening. In this study, we directly tested the activities of five enzymes (FPase, CMCase, pNPCase, pNPGase and xylanase) in crude enzyme preparations from each mutant cultivated in the presence of Avicel as the sole carbon source. Through this approach, we identified two novel regulatory genes (*POX02484* and *POX08522*) that were not listed among the regulatory genes reported by Li et al. [9]. The novel transcription factors POX02484 and POX08522 extend the list of fungal gene regulators known to regulate cellulase and xylanase gene expression. These findings may be useful for the genetic engineering of filamentous fungi to improve cellulase and xylanase activities for use in bio-refinery applications.

## Conclusions

In this study, comparative genomic, transcriptomic and secretomic profiling of *Penicillium oxalicum* HP7-1 and its cellulase and xylanase hyper-producing mutant EU2106 were employed to screen for novel regulators of cellulase and xylanase gene expression. Two novel genes (*POX02484* and *POX08522*) were discovered and characterized to regulate the expression of cellulase and xylanase genes. These findings may prove important for engineering filamentous fungi to improve cellulase and xylanase production.

## Methods

### Strains and growth conditions

*P*. *oxalicum* strain HP7-1 (China General Microbiological Culture Collection (CGMCC) 10781) was isolated from a subtropical forest soil system in Guangxi Zhuang Autonomous Region, China [17], and its cellulase hyper-producing mutant, EU2106, (CGMCC 6471) was obtained by multiple rounds of mutagenesis [18]. The strains were maintained on potato-dextrose-agar (PDA) plates at 4 °C. In this study, the constructed deletion mutants Δ*PoxKu70*, Δ*PoxClrB*, Δ*POX02484* and Δ*POX08522* were respectively deposited in CGMCC with numbers of 3.15650, 3.15649, 3.15647 and 3.15648, respectively. *Penicillium oxalicum* strains were cultivated on PDA plates at 28 °C for 6 days to complete sporulation.

For gene expression assays for comparative transcriptomics, a spore suspension of 1 mL containing approximately 1.0 × 10^8^ spores per mL was inoculated into 100 mL of modified minimal medium (MMM) containing (g/L) KH_2_PO_4_ (4.0), (NH_4_)_2_SO_4_ (4.0), MgSO_4_·7H_2_O (0.60), CaCl_2_ (0.6), FeSO_4_·7H_2_O (0.0050), MnSO_4_ (0.001600), ZnCl_2_ (0.0017), CoCl_2_ (0.002), 1 mL of Tween, and Avicel and wheat bran that were added to a final concentration of 1 % (w/v) and 4 % (w/v), respectively, as the carbon source. Cultures were incubated at 28 °C with shaking at 180 rpm for 72 h. Mycelia were separated from the culture by an eight-layer filter fabric and washed three times using diethyl pyrocarbonate-treated water prior to RNA extraction.

For gene deletion mutants, Avicel at a final concentration of 2 % (w/v) in 100 mL of MMM was used as sole carbon source. The inoculated medium was incubated at 28 °C with shaking at 180 rpm for 6 days. The supernatant was collected after centrifugation at 11,300*g* for 10 min, and filtered through a Whatman No. 1 filter paper for enzyme activity and secretome analyses, while the mycelia were collected for DNA extraction.

A shift experiment was performed for qRT-PCR analysis of gene expression and measurement of enzymatic activity in *P*. *oxalicum*. Cultures containing approximately 1.0 × 10^8^ spores per mL were pre-grown for 20 h in MMM containing 1 % (w/v) glucose at 28 °C with shaking at 180 rpm. Then, equal portions of the harvested and washed mycelial sample were aseptically re-placed into MMM containing 2 % Avicel as the sole carbon source. After cultivation at 28 °C with shaking at 180 rpm for 4, 12 and 24 h, mycelia were harvested for qRT-PCR assay, while supernatants were collected at 3 and 5 days for enzymatic activity assays.

### DNA and RNA extraction

Total DNA was extracted from mycelia using the modified phenol-chloroform method [37]. Briefly, after washing with sterile water, mycelia were immediately ground in liquid nitrogen, and 1 mL of lysate reagent [40 mM Tris-HCl, 20 mM sodium acetate, 10 mM ethylenediaminetetraacetic acid (EDTA), and 1 % sodium dodecyl sulfate, pH 8.0] was added per 100 mg of powder. Whole genomic DNA was collected after precipitation via centrifugation at 11,300*g* for 10 min.

Total RNA was extracted from mycelia using the TRIzol RNA Kit (Life Technologies, Carlsbad, CA, USA) according to the manufacturer’s instructions. The concentration and integrity of the extracted RNA were determined by measuring the absorbance at 260 and 280 nm (the A_260_/A_280_ ratio) by spectrophotometer and electrophoresis on 1 % agarose gels, respectively.

### Genome sequencing, assembly, and gene prediction and annotation

The genome of *P. oxalicum* strain HP7-1 was sequenced using an Illumina HiSeq 2000 system (Illumina, San Diego, CA, USA) at the Beijing Genomics Institute (Shenzhen, China). Genomic DNA was sheared randomly to construct three read libraries with lengths of 170, 500 and 6000 bp by a Bioruptor ultrasonicator (Diagenode, Denville, NJ, USA) and physico-chemical methods. The paired-end fragment libraries were sequenced according to the Illumina HiSeq 2000 system’s protocol. The sequenced reads were assembled using SOAPdenovo v1.05 software (http://soap.genomics.org.cn/soapdenovo.html), while Augustus v2.6.1 (http://bioinf.uni-greifswald.de/augustus/), GeneMark v2.3e (http://exon.gatech.edu/) and GeneWise v2.20 (http://www.sanger.ac.uk/Software/Wise2/) were used for gene prediction. The predicted genes were functionally annotated by homologous alignment using the GO, KEGG, UniProt, Cluster of Orthologous Groups of proteins (COG), and NCBI NR databases, using the BLAST algorithm (http://www.ncbi.nlm.nih.gov/blast/executables/blast+/LATEST/). Transfer RNA and rRNA were detected using RNAmmer v1.2 (http://www.cbs.dtu.dk/services/RNAmmer/) and tRNAscan-SE v1.23 (http://gtrnadb.ucsc.edu/) software, respectively. Secreted proteins were predicted by both SignalP v3.0 (http://www.cbs.dtu.dk/services/SignalP/) and TargetP v1.1 (http://www.cbs.dtu.dk/services/TargetP/). Transcription factors were annotated according to their InterPro IDs in the Fungal Transcription Factor Database [38]. Transporters were identified and classified by BLASTP searches of the TransportDB database (http://www.membranetransport.org) with an *E* value threshold of 1e−5. CAZymes were predicted by searching the dbCAN database (http://csbl.bmb.uga.edu/dbCAN/), with an *E* value threshold of 1e−7, F3 = 1e−10, F2 = 1e−7, and F1 = 0.001.

### Transcription profiling data assays

Samples for transcriptional profiling were collected after 72 h of induction in the presence of wheat bran and Avicel. Three biological replicates of each sample were analyzed. A cDNA library was constructed, tested by an Agilent 2100 Bioanalyzer (Agilent Technologies, Santa Clara, CA, USA) and an ABI StepOnePlus Real-Time PCR System (Applied Biosystems, Foster City, CA, USA), and subsequently sequenced using the Illumina HiSeq 2000 system. The generated clean reads were mapped to the *P*. *oxalicum* HP7-1 genome using BWA v0.7.10-r789 (http://sourceforge.net/projects/bio-bwa/files/) and Bowtie2 v2.1.0 software [39]. The gene expression level (fragments per kilobase of exon per million mapped reads) was analyzed with RSEM v1.2.12 software [40]. Differentially expressed genes were detected using the NOISeq tool (http://www.bioconductor.org/packages/release/bioc/html/NOISeq.html) (log_2_ fold change| ≥ 1, *P* ≤ 0.01 and probability ≥ 0.8 as thresholds). Blast2GO v2.3.5 (https://www.blast2go.com), and BLAST v2.2.26 (http://blast.ncbi.nlm.nih.gov/Blast.cgi) was used for gene homology and function annotation.

### Comparative secretome assays

Culture supernatants from *P*. oxalicum strains HP7-1 and EU2106 were filtered through a 0.22 μm membrane filter (Pall Corp., Ann Arbor, MI, USA) after adding two cOmplete, EDTA-free tablets (cOmplete EASYpack, Roche, Basel, Switzerland) per 100 mL of medium, and subsequently concentrated by ultrafiltration with a 10 kDa centrifugal concentrator (Millipore, Darmstadt, Germany). Secreted proteins were precipitated with acetone at −20 °C overnight. The collected proteins were denatured, digested, and subsequently labelled using the isobaric tags for relative and absolute protein quantitation Reagent 8 Plex One Assay Kit (AB Sciex, Framingham, MA, USA) according to the manufacturer’s protocol. The labelled peptides were separated on an XBridge C18 chromatographic column (250 mm × 4.6 mm, 3.5 μm, Waters, Milford, MA, USA). The collected peptide mixtures were then desalted with a ZipTip C18 column (Sigma-Aldrich, St. Louis, MO, USA) and dissolved in 10 μL of 2 % (v/v) acetonitrile in 0.1 % (v/v) formic acid for the liquid chromatography coupled with tandem mass spectrometry (LC–MS/MS) assay. After screening using the Proteome Discovery with SEQUEST search engine software (Thermo Fisher Scientific, Waltham, MA, USA), LC–MS/MS data were mapped to the *P*. *oxalicum* protein database and analyzed.

### Protoplast preparation and transformation

*Penicillium oxalicum* protoplasts were prepared as described by Churchill et al. [41], with some modifications. Fresh conidia were inoculated into 200 mL of CM medium containing 50 ml of 20× nitrate [(g/L) NaNO_3_ (120.0), KCl (10.40), MgSO_4_·7H_2_O (10.40), KH_2_PO_4_ (30.40)], d-glucose (10.0), peptone (2.0), yeast extract (1.0), and acid hydrolysed casein (1.0) at pH 6.5, and cultivated at 28 °C with shaking at 180 rpm for 8 h. Mycelia were then harvested, washed three times with 0.6 M MgSO_4_·7H_2_O, and lysed in OM solution (1.2 M MgSO_4_·7H_2_O, 10 mM NaH_2_PO_4_, 6 g/L snailase, 4 g/L lysozyme, and 6 g/L lysing enzymes from *T. harzianum* (Sigma-Aldrich) at pH 5.8 for 2.5 h. Protoplasts were separated by adding trapping buffer (0.4 M sorbitol and 0.1 M Tris-HCl at pH 7.0), and subsequently collected in 50 mL centrifuge tubes. The collected protoplasts were precipitated using 1 M sorbitol and washed at least three times with 30 mL of STC (1 M sorbitol, 0.1 M Tris-HCl, and 0.1 M CaCl_2_ at pH 8.0). Finally, the protoplasts were resuspended in 0.5 mL of 4× STC and 1× PTC (40 % PEG3350, 0.1 M Tris-HCl, and 0.1 M CaCl_2_ at pH 8.0), adjusted to a concentration of 2 × 10^7^ protoplasts per mL, and stored at −80 °C for further study.

For transformations, approximately 5 μg of DNA fragments were dissolved in 10 μL of 0.1 M spermidine and added to 100 μL of the protoplast suspension, followed by incubation on ice for 30 min. The mixture of DNA fragments and protoplasts was cultured for 25 min at room temperature after adding 1 mL of PTC. The cultivated mixture was added to 50 mL of OCM medium at 50 °C containing (g/L) casein enzymatic hydrolysate (1.0), yeast extract (1.0), sucrose (273.6), and agar (10.0), mixed briefly, and poured into Petri plates. After 30 min, PDA medium containing 250 μg/mL hygromycin B and 500 μg/mL G418 was added to the OCM medium. Transformants became visible after 5 days of cultivation at 28 °C.

### Construction of gene deletion mutants

The Δ*PoxKu70* strain derived from *P*. *oxalicum* strain HP7-1 was constructed to improve the efficiency of homologous recombination, as described by Li et al. [26], with some modifications. Briefly, approximately 2 kb of upstream and downstream flanking sequences of the *PoxKu70* gene and 2.1 kb of the hygromycin phosphotransferase (*hph*) resistance gene were amplified from the genomic DNA of *P*. *oxalicum* strain HP7-1 and plasmid pCPXHY2GFP, respectively, which were maintained in our laboratory, and fused to construct the knockout cassette by double-joint PCR. The knockout cassette was introduced into wild-type *P. oxalicum* HP7-1 protoplasts as described above to generate the Δ*PoxKu70* strain, in which the *Poxku70* gene was replaced by the *hph* gene.

Subsequently, candidate transcription factor genes were knocked out in the Δ*PoxKu70* strain using the method for deleting the *PoxKu70* gene in *P*. *oxalicum* strain HP7-1. In this instance, the G418 resistance gene, which was amplified from the plasmid pCPXG418 and maintained in our laboratory, was used to replace the target gene. Three transformants for each gene knockout were randomly chosen for further study.

### Southern hybridization analysis

Probes used for southern hybridization were amplified with the primers shown in Additional file 11: Table S5. Genomic DNA of the five mutants (Δ*PoxKu70,* Δ*PoxClrB*, Δ*POX02484,* Δ*POX07291* and Δ*POX08522*) was digested with *Apa*I, *Sac*I, *Eco*RV and/or *Bgl*I (TaKaRa, Shiga, Japan). Enzyme-digested products were separated by 0.8 % agarose gel and transferred to Hybond-N^+^ nylon membranes (GE Healthcare Limited, Buckinghamshire, UK). A DIG-High prime DNA labelling and detection starter kit (Life Technologies, Carlsbad, CA, USA) was used to label and detect DNA according to the manufacturer’s protocol.

### Measurement of biomass dry weight

Biomass formation of *P*. *oxalicum* strains in medium containing glucose as sole carbon source was determined gravimetrically as previously described [42]. Biomass concentration in medium containing Avicel as sole carbon source was indirectly measured by calculating the amount of essential intracellular proteins [42]. Briefly, the solid fraction was collected from the culture after centrifugation and immediately ground in liquid nitrogen, and 1 mL of protein extraction reagent consisting of PBS buffer containing 5 mM EDTA, 5 mM Phenylmethanesulfonyl fluoride (PMSF), and one complete ULTRA protease inhibitor cocktail tablet (Life Technologies, Carlsbad, CA, USA) at pH 7.4 was added per 100 mg of powder. Total protein was collected after precipitation via centrifugation at 11,300*g* for 10 min. Protein concentration was determined by the Bradford method (TIANQEN, Beijing, China). The final protein content was corrected using a set of substrate controls in which no inoculum was added to the Avicel medium. The biomass dry weight was calculated assuming an average content of 0.28 g intracellular protein per gram of dry cell mass. All experiments were repeated three times and the mean of the three experiments is reported.

### Enzyme activity and protein concentration assays

Enzyme activities, including FPase, CMCase (EG), xylanase, Avicelase, KSBase, pNPCase (CBH), and pNPGase (BGL) activities, were determined as previously reported [5, 9]. FPase activity was investigated by incubating Whatman No. 1 filter paper (50 mg, 1.0 × 6.0 cm) in a 1.5 mL reaction system containing 1.0 mL of citrate buffer (100 mM, pH 5.0) and 0.5 mL of suitably diluted crude cellulase for 1 h at 50 °C. CMCase activity was measured in a 0.5 mL reaction system containing 0.45 mL of 1.0 % CMC-Na solution in citrate buffer (100 mM, pH 5.0) and 0.05 mL of crude cellulase. This mixture was incubated at 50 °C for 30 min. Xylanase, Avicelase, and KSBase activities were determined under similar conditions, except that 1.0 % xylan, 1.0 % Avicel, or 1.0 % KSB were used as substrates in place of CMC-Na, and the incubation time was 10, 60, or 30 min, respectively. The concentration of reducing sugars produced, in terms of glucose or xylose equivalents, was measured at 540 nm after adding two volumes of 3,5-dinitrosalicylic acid. One unit of enzymatic activity was defined as the amount of enzyme capable of producing 1 μmol of glucose or xylose from the appropriate substrates per min.

Measurement of pNPCase and pNPGase activity was conducted using *p*-nitrophenyl-β-cellobioside (pNPC) and *p*-nitrophenyl-β-glucopyranoside (pNPG), respectively, as substrates. A 140 μL mixture comprising 116 μL of citrate buffer (100 mM, pH 5.0), 14 μL of 25 mM pNPC or pNPG solution, and 10 μL of diluted crude cellulase was incubated at 50 °C for 15 min. Liberated *p*-nitrophenol was measured at 410 nm after adding 70 μL of sodium carbonate (0.4 M). One unit of enzyme activity was defined as the amount of enzyme capable of producing 1 μmol of *p*-nitrophenol from the appropriate substrates per min. Triplicate experiments were analyzed independently for each sample.

Determination of total protein concentration was performed using a Bradford assay kit (Pierce Biotechnology, Rockford, IL, USA) according to the manufacturer’s instructions.

### qRT-PCR

The expression levels of cellulase and hemicellulase genes in mutant and wild-type strains were measured by qRT-PCR on a LightCycle480 instrument with version 4.0 software (Life Technologies, Carlsbad, CA, USA). First-strand cDNA was synthesized using the PrimeScript RT Reagent Kit with gDNA Eraser (TaKaRa, Shiga, Japan) according to the manufacturer’s instructions. Each qRT-PCR was conducted in a final volume of 20 μL that contained 6.4 μL of sterile water, 0.8 μL of 10 μM of the corresponding primers, 2.0 μL of suitable cDNA as the template, and 10 μL of SYBR Premix ExTaq II (TaKaRa, Shiga, Japan). All reactions were run for 40 cycles, comprising 3 s at 95 °C and 30 s at 60 °C. The melting curve program was set for 5 s at 95 °C, 1 min at 60 °C, and 15 s at 95 °C. The fluorescence signal was measured at the end of each extension step at 80 °C. Gene expression levels for cellulase and xylanase genes were measured using actin gene (*POX09428*) as a control and normalized to the parental strain Δ*PoxKu70*. Primers used for qRT-PCR analysis are shown in Additional file 11: Table S5. All qRT-PCRs were independently repeated in triplicate.

### Phylogenetic analysis

POX02484 and POX08522 homologs were identified from NCBI (http://blast.ncbi.nlm.nih.gov/) using BLASTP, and phylogenetic tree analysis was performed using MEGA software version 7.0 [43] with the neighbour-joining method and a Poisson correction model. In this process, 1000 replicates were used for bootstrap values and gaps, and missing data treatments.

### Statistics analysis

The Student’s *t* test (two-tailed) was performed using Microsoft Excel (Office 2013) (Microsoft, Redmond, WA, USA).

### Sequence accession numbers

The genome sequence of *P*. *oxalicum* strain HP7-1 reported in this study was deposited in the GenBank database under accession number JRVD00000000. The re-sequenced clean data of *P*. *oxalicum* mutant EU2106 was submitted to SRA (http://www.ncbi.nlm.nih.gov/sra) under accession number SRA399107. The transcriptome of *P*. *oxalicum* HP7-1 and EU2106 grown on wheat bran and Avicel as the carbon source were respectively deposited at the GEO (http://www.ncbi.nlm.nih.gov/geo/) and the SRA databases under accession numbers GSE80076 and SRA399500 for HP7-1 and SRA399511 for EU2106. The secretomes of *P*. *oxalicum* HP7-1 and EU2106 grown on wheat bran and Avicel as the carbon source were submitted to the proteomics identifications (PRIDE) database (http://www.ebi.ac.uk/pride/archive/) under the accession numbers PXD004046. Sequences of *PoxClrB*, *POX02484* and *POX08522* from *P*. *oxalicum* HP7-1 and EU2106 were deposited in the GenBank database under accession numbers KU597415 to KU597419.

## Additional files

10.1186/s13068-016-0616-9 Venn diagram showing unique and shared proteins in *P. oxalicum* strain HP7-1 annotated using the non-redundant (NR), UniProt, Kyoto Encyclopedia of Genes and Genomes (KEGG), and Gene Ontology (GO) databases.
10.1186/s13068-016-0616-9 List of 274 single nucleotide variations and 12 Insertion/deletions (InDels) that occurred in *P*. *oxalicum* mutant strain EU2106 compared with the wild-type strain HP7-1.
10.1186/s13068-016-0616-9 List of 347 genes displaying altered expression in the cellulase and xylanase hyper-producing mutant *P*. *oxalicum* strain EU2106 compared with the wild-type strain HP7-1 when cultivated in medium containing wheat bran and Avicel as the carbon source.
10.1186/s13068-016-0616-9 Gene expression profiles of *P. oxalicum* strains HP7-1 and EU2106 during growth in the presence of wheat bran and Avicel as the carbon source. (A) Gene expression profile (|log_2_ fold change| ≥ 1, *P* ≤ 0.01 and probability ≥ 0.8 were used as thresholds). (B) Up-regulated genes encoding plant cell wall degrading enzymes (CWDEs) and major facilitator superfamily (MFS) members. (C) Down-regulated genes encoding CWDEs and MFS members. The expression scale is represented as the Log_2_ fold-change in B and C.
10.1186/s13068-016-0616-9 Functional annotation of secreted proteins as determined using the Kyoto Encyclopedia of Genes and Genomes database, and comparative analysis of the proteins identified in *P*. *oxalicum* strains HP7-1 and 114-2.
10.1186/s13068-016-0616-9 List of 240 proteins secreted by *P*. *oxalicum* strains HP7-1 and EU2106 when grown in medium containing wheat bran and Avicel as the carbon source.
10.1186/s13068-016-0616-9 Eleven candidate transcription factors identified from the initial screen.
10.1186/s13068-016-0616-9 Phenotypic comparison of wild-type *P*. *oxalicum* strain HP7-1 and the Δ*PoxKu70* mutant. (A) Comparison of growth on potato-dextrose-agar plates between HP7-1 and Δ*PoxKu70*. (B–E) Comparison of the sensitivity to different concentrations of hygromycin B, G418, and ultraviolet light, as well as the cellulase/xylanase activities, between HP7-1 and Δ*PoxKu70.*

10.1186/s13068-016-0616-9 PCR and Southern hybridization analysis of deletion mutants of four candidate genes derived from the Δ*PoxKu70* parent strain. (A) Mutant Δ*PoxClrB*, M, 1 kb marker; 1, Δ*PoxKu70*; 2, ddH_2_O; 3, Δ*PoxClrB*-1; 4, Δ*PoxClrB*-2; 5, Δ*PoxClrB*-3; (B) Mutant Δ*POX02484*, M, 1 kb marker; 1, Δ*PoxKu70*; 2, ddH_2_O; 3, Δ*POX02484*-6; 4, Δ*POX02484*-10; 5, Δ*POX02484*-17; (C) Mutant Δ*POX07921*, M, 1 kb marker; 1, Δ*PoxKu70*; 2, ddH_2_O; 3, Δ*POX07291*-1; 4, Δ*POX07291*-2; 5, Δ*POX07291*-3; (D) Mutant Δ*POX08522*, M, 1 kb marker; 1, Δ*PoxKu70*; 2, ddH_2_O; 3, Δ*POX08522*-2; 4, Δ*POX08522*-11; 5, Δ*POX08522*-13; (E) Southern hybridization of Δ*PoxClrB*, M, 1 kb marker; 1, Δ*PoxKu70*; 2, Δ*PoxClrB*-1; 3, Δ*PoxClrB*-2; 4, Δ*PoxClrB*-3; (F) Southern hybridization of Δ*POX02484*, M, 1 kb marker; 1, Δ*PoxKu70*; 2, Δ*POX02484*-6; 3, Δ*POX02484*-10; 4, Δ*POX02484*-17; (G) Southern hybridization of Δ*POX07291*, M, 1 kb marker; 1, Δ*PoxKu70*; 2, Δ*POX07291*-1; 3, Δ*POX07291*-2; 4, Δ*POX07291*-3; (H) Southern hybridization of Δ*POX08522*, M, 1 kb marker; 1, Δ*PoxKu70*; 2, Δ*POX08522*-2; 3, Δ*POX08522*-11; 4, Δ*POX08522*-13. In A–D, the top figure shows the production of each target gene, the middle figure shows the production of the fragment on the left of the target gene, and the bottom figure shows the production of the fragment on the right of the target gene.
10.1186/s13068-016-0616-9 Activities of crude enzymes from *PoxClrB*, *POX02484*, and *POX08522* deletion mutants following direct inoculation in Avicel. Crude enzymes were produced by fungal strains grown in 1.0 % Avicel as the sole carbon source. The symbols * and ** indicate significant differences (*P* ≤ 0.05 and *P* ≤ 0.01, respectively) between candidate mutants and the Δ*PoxKu70* parent strain, as assessed by Student’s *t* test.
10.1186/s13068-016-0616-9 Primers used in this study.

## Abbreviations

BGL: β-glucosidase
BLASTP: protein basic local alignment search tool
CBH: cellobiohydrolase
CAZymes: carbohydrate-active enzymes
CDSs: coding DNA sequences
CMCase: carboxymethylcellulose cellulase
CWDEs: plant cell wall-degrading enzymes
EDTA: ethylenediaminetetraacetic acid
EG: endo-glucanase
FPase: filter paper cellulase
GEO: gene expression omnibus
GO: gene ontology
Inter: intergenic gene
hph: the hygromycin phosphotransferase
InDels: insertion/deletions
KEGG: Kyoto Encyclopedia of Genes and Genomes
KSBase: KOH-pretreated sugarcane bagasse
LC-MS/MS: the liquid chromatography coupled with tandem mass spectrometry
MFS: major facilitator family
MMM: modified minimal medium
NCBI NR: National Center for Biotechnology Information non-redundant
PMSF: phenylmethanesulfonyl fluoride
pNPGase: *p*-nitrophenyl-β-glucopyranoside cellulase
PCR: polymerase chain reaction
PDA: potato-dextrose-agar
pNPCase: *p*-nitrophenyl-β-cellobioside cellulase
PRIDE: the proteomics identifications
qRT-PCR: real-time quantitative reverse transcription-PCR
SB: sugarcane bagasse
SNVs: single nucleoside variations
SRA: sequence read archive
