# Supplementary figures and images for: Comparative genomic, transcriptomic and secretomic profiling of Penicillium oxalicum HP7-1 and its cellulase and xylanase hyper-producing mutant EU2106, and identification of two novel regulatory genes of cellulase and xylanase gene expression
Source: Biotechnol Biofuels. 2016 Sep 23;9:203. doi: 10.1186/s13068-016-0616-9 (PMC5035457; doi:10.1186/s13068-016-0616-9)

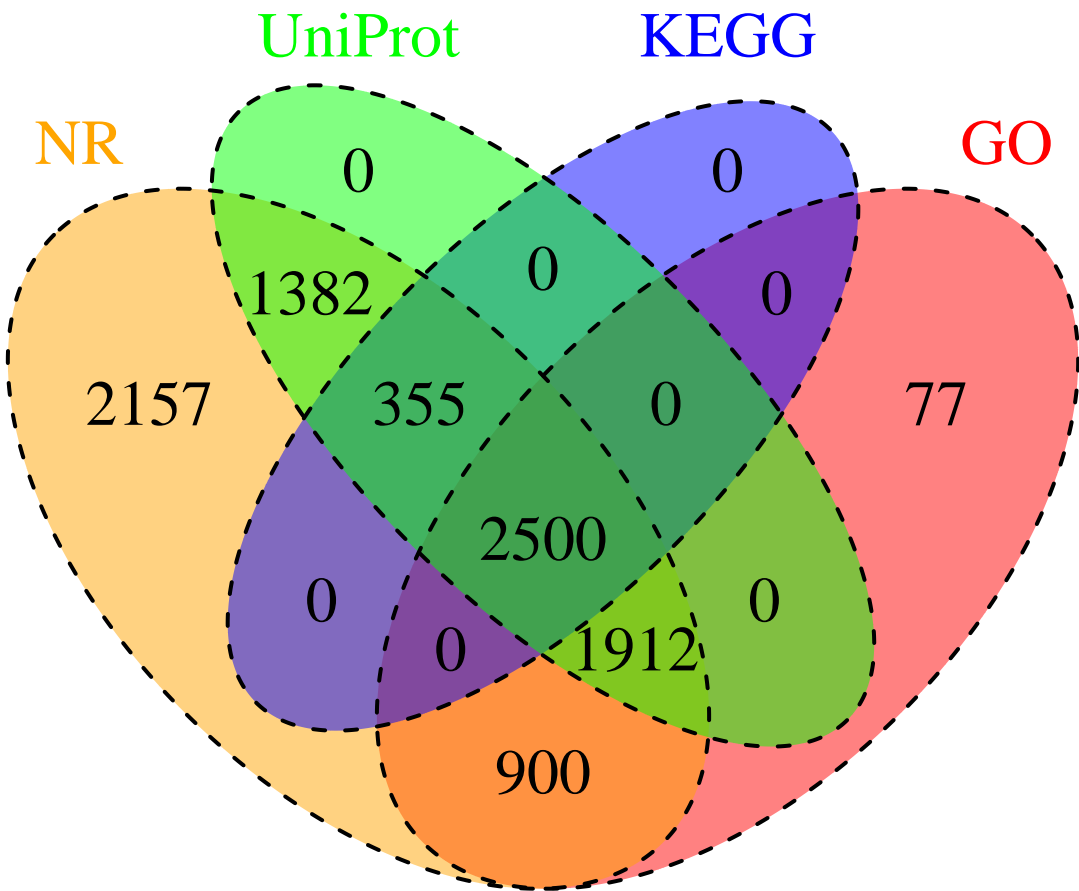

Supplement: Supplementary file 1 — 10.1186/s13068-016-0616-9 Venn diagram showing unique and shared proteins in P. oxalicum strain HP7-1 annotated using the non-redundant (NR), UniProt, Kyoto Encyclopedia of Genes and Genomes (KEGG), and Gene Ontology (GO) databases. [file 13068_2016_616_MOESM1_ESM.pdf]

**A**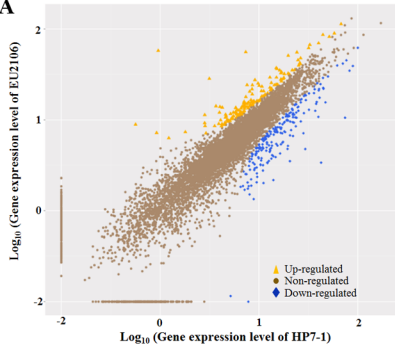**B**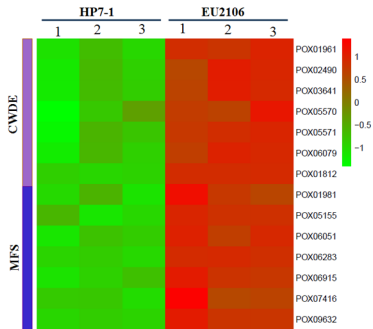**C**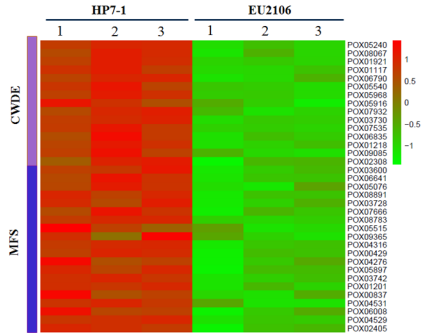

Supplement: Supplementary file 4 — 10.1186/s13068-016-0616-9 Gene expression profiles of P. oxalicum strains HP7-1 and EU2106 during growth in the presence of wheat bran and Avicel as the carbon source. (A) Gene expression profile (|log2 fold change| ≥ 1, P ≤ 0.01 and probability ≥ 0.8 were used as thresholds). (B) Up-regulated genes encoding plant cell wall degrading enzymes (CWDEs) and major facilitator superfamily (MFS) members. (C) Down-regulated genes encoding CWDEs and MFS members. The expression scale is represented as the Log2 fold-change in B and C. [file 13068_2016_616_MOESM4_ESM.pdf]

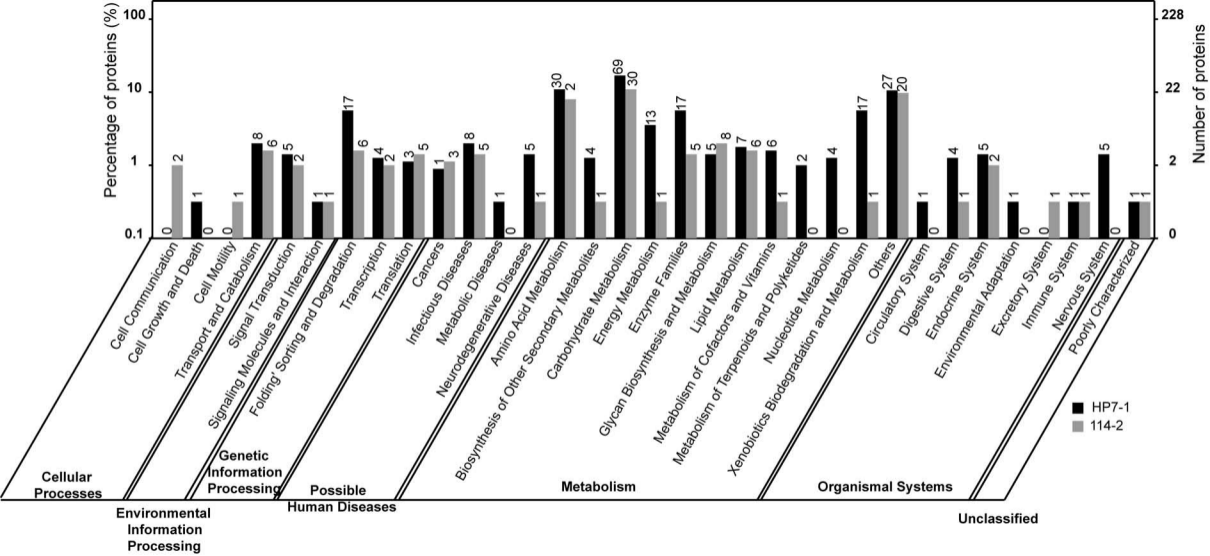

Supplement: Supplementary file 5 — 10.1186/s13068-016-0616-9 Functional annotation of secreted proteins as determined using the Kyoto Encyclopedia of Genes and Genomes database, and comparative analysis of the proteins identified in P. oxalicum strains HP7-1 and 114-2. [file 13068_2016_616_MOESM5_ESM.pdf]

**A**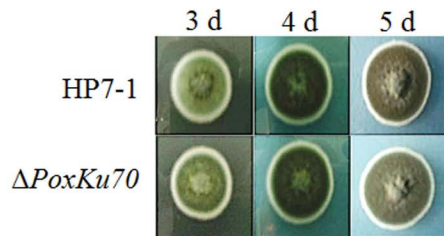**B**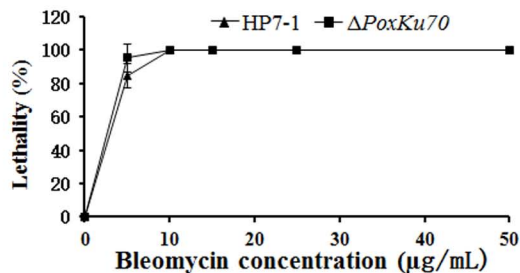**C**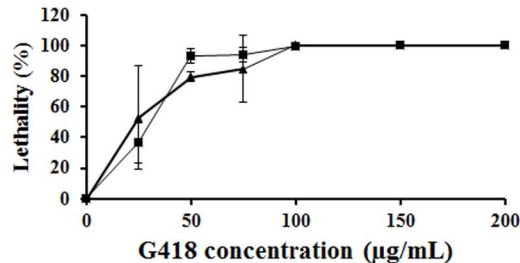**D**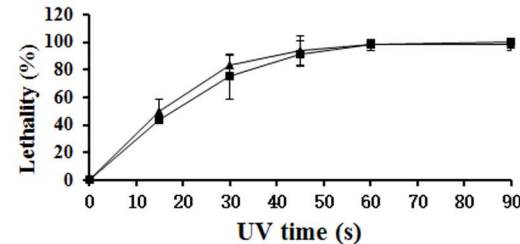**E**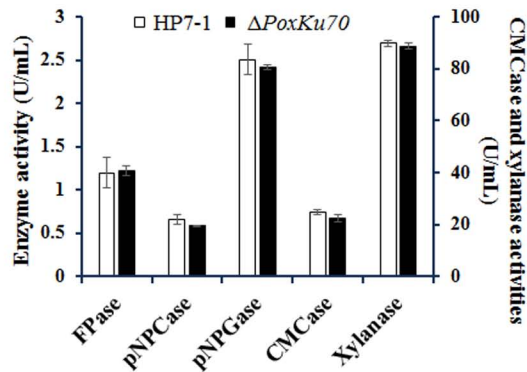

Supplement: Supplementary file 8 — 10.1186/s13068-016-0616-9 Phenotypic comparison of wild-type P. oxalicum strain HP7-1 and the ΔPoxKu70 mutant. (A) Comparison of growth on potato-dextrose-agar plates between HP7-1 and ΔPoxKu70. (B–E) Comparison of the sensitivity to different concentrations of hygromycin B, G418, and ultraviolet light, as well as the cellulase/xylanase activities, between HP7-1 and ΔPoxKu70. [file 13068_2016_616_MOESM8_ESM.pdf]

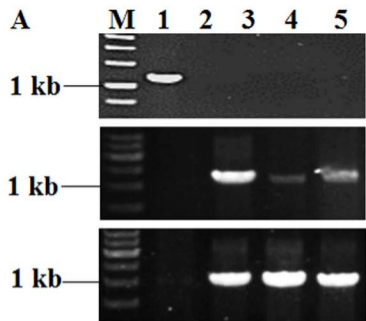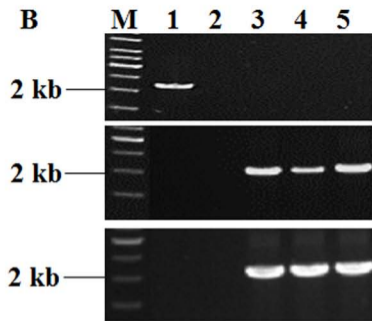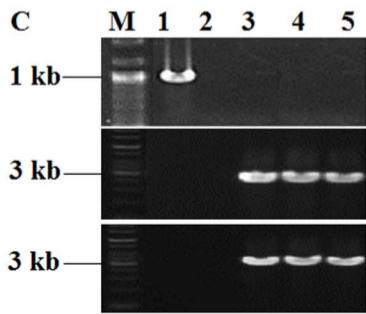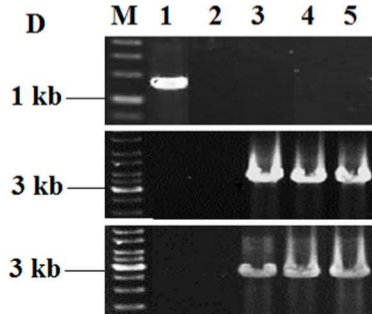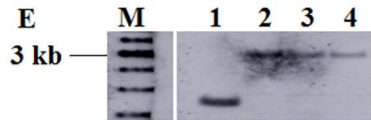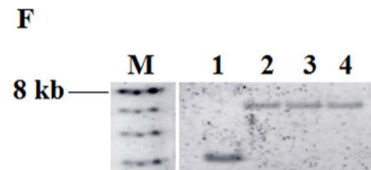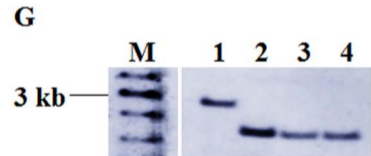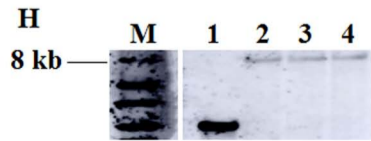

Supplement: Supplementary file 9 — 10.1186/s13068-016-0616-9 PCR and Southern hybridization analysis of deletion mutants of four candidate genes derived from the ΔPoxKu70 parent strain. (A) Mutant ΔPoxClrB, M, 1 kb marker; 1, ΔPoxKu70; 2, ddH2O; 3, ΔPoxClrB-1; 4, ΔPoxClrB-2; 5, ΔPoxClrB-3; (B) Mutant ΔPOX02484, M, 1 kb marker; 1, ΔPoxKu70; 2, ddH2O; 3, ΔPOX02484-6; 4, ΔPOX02484-10; 5, ΔPOX02484-17; (C) Mutant ΔPOX07921, M, 1 kb marker; 1, ΔPoxKu70; 2, ddH2O; 3, ΔPOX07291-1; 4, ΔPOX07291-2; 5, ΔPOX07291-3; (D) Mutant ΔPOX08522, M, 1 kb marker; 1, ΔPoxKu70; 2, ddH2O; 3, ΔPOX08522-2; 4, ΔPOX08522-11; 5, ΔPOX08522-13; (E) Southern hybridization of ΔPoxClrB, M, 1 kb marker; 1, ΔPoxKu70; 2, ΔPoxClrB-1; 3, ΔPoxClrB-2; 4, ΔPoxClrB-3; (F) Southern hybridization of ΔPOX02484, M, 1 kb marker; 1, ΔPoxKu70; 2, ΔPOX02484-6; 3, ΔPOX02484-10; 4, ΔPOX02484-17; (G) Southern hybridization of ΔPOX07291, M, 1 kb marker; 1, ΔPoxKu70; 2, ΔPOX07291-1; 3, ΔPOX07291-2; 4, ΔPOX07291-3; (H) Southern hybridization of ΔPOX08522, M, 1 kb marker; 1, ΔPoxKu70; 2, ΔPOX08522-2; 3, ΔPOX08522-11; 4, ΔPOX08522-13. In A–D, the top figure shows the production of each target gene, the middle figure shows the production of the fragment on the left of the target gene, and the bottom figure shows the production of the fragment on the right of the target gene. [file 13068_2016_616_MOESM9_ESM.pdf]

Relative enzyme activity (%)

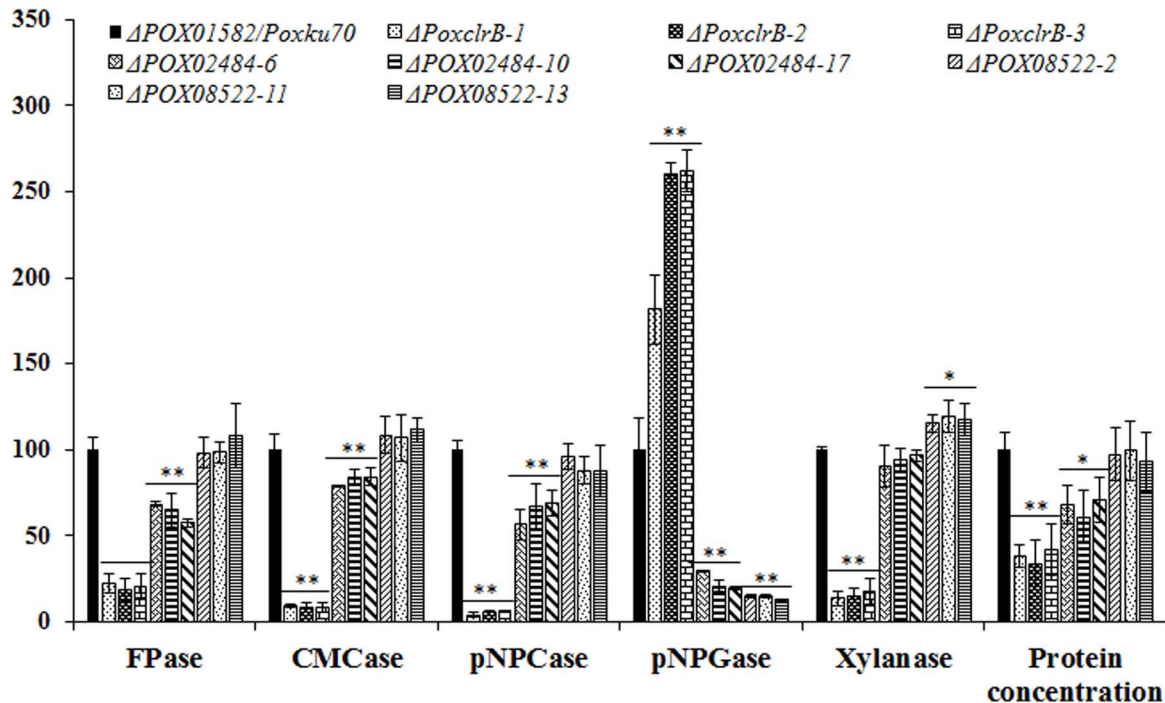

Supplement: Supplementary file 10 — 10.1186/s13068-016-0616-9 Activities of crude enzymes from PoxClrB, POX02484, and POX08522 deletion mutants following direct inoculation in Avicel. Crude enzymes were produced by fungal strains grown in 1.0 % Avicel as the sole carbon source. The symbols * and ** indicate significant differences (P ≤ 0.05 and P ≤ 0.01, respectively) between candidate mutants and the ΔPoxKu70 parent strain, as assessed by Student’s t test. [file 13068_2016_616_MOESM10_ESM.pdf]
