# Supplementary material for: Comparative genomic, transcriptomic and secretomic profiling of Penicillium oxalicum HP7-1 and its cellulase and xylanase hyper-producing mutant EU2106, and identification of two novel regulatory genes of cellulase and xylanase gene expression
Source: Biotechnol Biofuels. 2016 Sep 23;9:203. doi: 10.1186/s13068-016-0616-9 (PMC5035457; doi:10.1186/s13068-016-0616-9)
Supplement: Supplementary file 2 — 10.1186/s13068-016-0616-9 List of 274 single nucleotide variations and 12 Insertion/deletions (InDels) that occurred in P. oxalicum mutant strain EU2106 compared with the wild-type strain HP7-1. [file 13068_2016_616_MOESM2_ESM.pdf]

Additional file 2: Table S1. List of 274 single nucleotide variations (SNVs) and 12 Insertion/deletions (InDels) that occurred in *P. oxalicum* mutant strain EU2106 compared with the wild-type strain HP7-1.

	Gene ID	Functional annotation	Predicted transcription factor	CWDE	CAZy family	Mutation	Nucleoside position	Amino acid position	Amino acid mutation
**SNVs**									
**SNVs in CDSs**									
**Non-synonymous**	POX00008	exo-alpha-L-1, 5-arabinanase	NA	Exo-alpha-L-1, 5-arabinanase	GH33; GH93	C<->T	670	224	L<->F
	POX00193	hypothetical protein	NA	NA	NA	C<->T	500	167	S<->L
	POX00363	hypothetical protein	NA	NA	NA	G<->A	1193	398	G<->E
	POX00764	hypothetical protein	NA	NA	NA	C<->T	868	290	P<->S
	POX01100	hypothetical protein	NA	NA	NA	G<->A	5225	1742	G<->D
	POX01430	hypothetical protein	NA	NA	NA	G<->A	4693	1565	A<->T
	POX01441	hypothetical protein	NA	NA	NA	G<->A	331	111	A<->T
	POX01462	hypothetical protein	NA	NA	NA	G<->A	1597	533	D<->N
	POX01583	hypothetical protein	NA	NA	NA	G<->A	853	285	V<->I
	POX01960	cellulolytic transcriptional activator ClrB	Zn2Cys6: transcriptional regulatory protein, N-terminal	NA	NA	G<->A	2174	725	S<->N
	POX02233	hypothetical protein	NA	NA	NA	G<->A	887	296	E<->K
	POX02289	hypothetical protein	NA	NA	NA	G<->A	377	126	E<->K
	POX02603	hypothetical protein	NA	NA	NA	C<->T	1066	356	T<->I
	POX02808	hypothetical protein	NA	NA	NA	G<->A	421	141	G<->R
	POX02823	hypothetical protein	NA	NA	NA	G<->A	1823	608	R<->K
	POX03102	hypothetical protein	NA	NA	NA	G<->A	580	194	A<->T
	POX03177	putative dolichyl-phosphate beta-D-mannosyl transferase	NA	NA	GT27; GT2; GT81	C<->T	199	67	H<->Y

	POX03199	hypothetical protein	CCR4-Not complex component, Not1	NA	NA	G<->A	2594	865	R<->H
	POX03365	hypothetical protein	NA	NA	NA	G<->A	1575	525	/M<->I
	POX03807	hypothetical protein	NA	NA	NA	G<->A	283	95	G<->R
	POX03862	hypothetical protein	NA	NA	NA	A<->G	902	301	K<->R
	POX03974	hypothetical protein	NA	NA	NA	G<->A	490	164	V<->I
	POX04097	hypothetical protein	NA	NA	NA	G<->A	325	109	V<->M
	POX04237	hypothetical protein	NA	NA	NA	C<->T	1594	532	L<->F
	POX04271	hypothetical protein	NA	NA	NA	C<->T	185	62	S<->F
	POX04288	hypothetical protein	NA	NA	NA	C<->T	104	35	T<->M
	POX04324	hypothetical protein	NA	NA	NA	C<->G	199	67	P<->A
	POX04473	hypothetical protein	NA	NA	NA	G<->A	1949	650	G<->D
	POX04606	hypothetical protein	NA	NA	NA	C<->T	1040	347	T<->M
	POX04630	hypothetical protein	NA	NA	NA	G<->A	748	250	E<->K
	POX04690	hypothetical protein	NA	NA	NA	T<->C	797	266	F<->S
	POX04745	hypothetical protein	NA	NA	NA	G<->A	952	318	V<->M
	POX04773	hypothetical protein	NA	NA	NA	G<->A	625	209	D<->N
	POX04876	hypothetical protein	NA	NA	NA	C<->G	41	14	T<->S
	POX05415	hypothetical protein	NA	NA	NA	G<->A	1252	418	D<->N
	POX05477	hypothetical protein	NA	NA	NA	C<->G	2964	988	D<->E
	POX05543	hypothetical protein	NA	NA	NA	G<->A	872	291	G<->D
	POX05699	hypothetical protein	NA	NA	AA3;AA8	G<->A	878	293	C<->Y
	POX06062	hypothetical protein	NA	NA	NA	G<->A	166	56	D<->N
	POX06242	putative alpha-glucosidase	NA	NA	GH31	G<->A	434	145	R<->K
	POX06319	hypothetical protein	NA	NA	NA	G<->A	1270	424	D<->N

	POX06497	G protein complex alpha subunit PGA3	NA	NA	NA	C<->T	467	156	A<->V
	POX06692	hypothetical protein	NA	NA	NA	C<->A	1033	345	Q<->K
	POX06695	hypothetical protein	NA	NA	NA	G<->A	695	232	G<->E
	POX06751	putative alpha-glucosidase	NA	NA	GH31	G<->A	430	144	V<->I
	POX06827	hypothetical protein	NA	NA	NA	G<->A	123	41	M<->I
	POX06916	hypothetical protein	NA	NA	NA	G<->A	304	102	A<->T
	POX07037	hypothetical protein	NA	NA	NA	G<->A	1813	605	D<->N
	POX07129	hypothetical protein	NA	NA	NA	C<->T	1747	583	P<->S
	POX07327	hypothetical protein	NA	NA	CE10	G<->A	106	36	G<->R
	POX07405	hypothetical protein	NA	NA	NA	G<->A	1363	455	G<->S
	POX07491	hypothetical protein	NA	NA	NA	C<->T	83	28	P<->L
	POX07567	putative UDP-Glc: sterol glucosyltransferase	NA	NA	GT1	G<->A	521	174	G<->E
	POX07604	hypothetical protein	NA	NA	NA	G<->A	937	313	E<->K
	POX07624	hypothetical protein	NA	NA	NA	G<->A	1079	360	S<->N
	POX08039	putative polysialyl transferase	NA	NA	GT4	G<->A	1064	355	G<->E
	POX08082	hypothetical protein	NA	NA	NA	G<->A	3233	1078	G<->D
	POX08119	hypothetical protein	NA	NA	NA	C<->T	347	116	T<->M
	POX08503	hypothetical protein	NA	NA	NA	G<->A	511	171	V<->M
	POX08522	hypothetical protein	Forkhead box	NA	NA	C<->T	1834	612	H<->Y
	POX08692	hypothetical protein	NA	NA	NA	G<->A	12309	4103	G<->S
	POX08695	hypothetical protein	NA	NA	NA	A<->C	1388	463	E<->A
	POX08862	hypothetical protein	NA	NA	NA	C<->T	4277	1426	A<->V
	POX08954	carbohydrate binding domain-	NA	NA	NA	C<->T	848	283	A<->V

		containing protein							
	POX09340	hypothetical protein	NA	NA	NA	C<->T	17023	5675	R<->C
	POX09812	hypothetical protein	NA	NA	NA	A<->G	185	62	N<->S
Premature_stop	POX02087	hypothetical protein	NA	NA	NA	C<->T	340	114	Q<->*
	POX03561	hypothetical protein	NA	NA	NA	C<->T	667	223	Q<->*
	POX03683	hypothetical protein	NA	NA	NA	G<->A	3435	1145	W<->*
	POX07858	hypothetical protein	NA	NA	NA	C<->T	715	239	R<->*
**Synonymous**	POX00035	hypothetical protein	NA	NA	NA	G<->A	333	111	–
	POX00329	hypothetical protein	NA	NA	NA	G<->A	1134	378	–
	POX00524	hypothetical protein	NA	NA	NA	C<->T	210	70	–
	POX00930	hypothetical protein	NA	NA	NA	C<->T	1602	534	–
	POX01118	hypothetical protein	Zn2Cys6: Fungal transcriptional regulatory protein, N-terminal	NA	NA	G<->A	1179	393	–
	POX01261	hypothetical protein	NA	NA	NA	G<->A	1401	467	–
	POX01662	hypothetical protein	NA	NA	NA	G<->A	618	206	–
	POX02080	hypothetical protein	NA	NA	NA	C<->T	180	60	–
	POX02101	hypothetical protein	NA	NA	NA	G<->A	441	147	–
	POX02188	hypothetical protein	NA	NA	NA	C<->T	144	48	–
	POX02545	hypothetical protein	NA	NA	NA	G<->A	483	161	–
	POX02610	hypothetical protein	NA	NA	NA	C<->T	27	9	–
	POX03225	hypothetical protein	NA	NA	NA	C<->T	213	71	–
	POX03546	hypothetical protein	NA	NA	NA	G<->A	2496	832	–
	POX04325	hypothetical protein	NA	NA	NA	C<->T	39	13	–
	POX04773	hypothetical protein	NA	NA	NA	C<->T	636	212	–
	POX04805	hypothetical protein	NA	NA	NA	G<->A	1686	562	–

	POX04995	beta-xylosidase/alpha-L-arabinofuranosidase	NA	beta-xylosidase/alpha-L-arabinofuranosidase	GH43	C<->T	93	31	–
	POX05436	hypothetical protein	Lambda repressor-like, DNA-binding	NA	NA	G<->A	426	142	–
	POX05512	hypothetical protein	NA	NA	NA	A<->T	186	62	–
	POX05999	hypothetical protein	NA	NA	NA	C<->T	1458	486	–
	POX06350	putative beta-galactosidase	NA	NA	GH35	A<->C	465	155	–
	POX06353	hypothetical protein	NA	NA	NA	C<->T	1185	395	–
	POX06494	hypothetical protein	Winged helix repressor DNA-binding	NA	NA	G<->A	2100	700	–
	POX06710	hypothetical protein	NA	NA	NA	G<->A	15	5	–
	POX06718	hypothetical protein	NA	NA	NA	C<->T	522	174	–
	POX07254	carbon catabolite repressor	C2H2 zinc finger	NA	NA	G<->T	435	145	–
	POX07295	hypothetical protein	NA	NA	NA	G<->A	321	107	–
	POX07722	hypothetical protein	NA	NA	NA	G<->A	303	101	–
	POX07767	hypothetical protein	NA	NA	NA	G<->A	1089	363	–
	POX08038	hypothetical protein	NA	NA	NA	G<->A	243	81	–
	POX08039	putative polysialyltransferase	NA	NA	GT4	G<->A	1053	351	–
	POX08165	hypothetical protein	NA	NA	NA	G<->A	1101	367	–
	POX09038	hypothetical protein	NA	NA	NA	G<->A	438	146	–
	POX09635	hypothetical protein	NA	NA	NA	C<->T	1020	340	–
**SNVs in Introns**									
**Intron**	POX00655	hypothetical protein	NA	NA	NA	G->A	1582	–	–
	POX01016	hypothetical protein	NA	NA	NA	G->T	1816	–	–
	POX01642	hypothetical protein	NA	NA	NA	G->A	600	–	–

	POX02115	hypothetical protein	NA	NA	NA	G->T	1279	–	–
	POX03021	putative chitinase	NA	NA	GH18	C->T	702	–	–
	POX04007	Zinc finger C2H2-type/integrase DNA-binding domain	C2H2 zinc finger	NA	NA	G->T	1478	–	–
	POX04760	hypothetical protein	NA	NA	NA	A->T	336	–	–
	POX04769	hypothetical protein	C2H2 zinc finger	NA	NA	G->T	1722	–	–
	POX04950	hypothetical protein	NA	NA	NA	A->T	411	–	–
	POX05006	hypothetical protein	Zn2Cys6: Fungal transcriptional regulatory protein, N-terminal	NA	NA	G->A	163	–	–
	POX05078	hypothetical protein	NA	NA	GH109	G->A	1338	–	–
	POX05365	hypothetical protein	NA	NA	NA	G->A	235	–	–
	POX05598	hypothetical protein	NA	NA	NA	C->T	447	–	–
	POX05984	hypothetical protein	NA	NA	NA	C->T	189	–	–
	POX06452	hypothetical protein	NA	NA	NA	G->T	407	–	–
	POX07733	hypothetical protein	NA	NA	NA	C->T	448	–	–
	POX08513	hypothetical protein	NA	NA	NA	C->T	1640	–	–
	POX08588	hypothetical protein	NA	NA	NA	C->T	257	–	–
	POX09056	putative alpha-mannosidase	NA	NA	GH47	C->T	2044	–	–
	POX09087	hypothetical protein	NA	NA	NA	G->T	2824	–	–
	POX09087	hypothetical protein	NA	NA	NA	G->A	2803	–	–
	POX09689	hypothetical protein	NA	NA	NA	G->A	321	–	–
	POX09689	hypothetical protein	NA	NA	NA	G->A	125	–	–
**SNVs in Intergenic genes**									
**1500-bp upstream of**	POX00189	hypothetical protein	NA	NA	NA	G->A	+30	–	–
	POX00241	hypothetical protein	C2H2 zinc finger	NA	NA	C->T	+38	–	–

**start code**	POX00301	hypothetical protein	NA	NA	NA	G->A	+87	–	–
	POX00344	hypothetical protein	NA	NA	NA	C->T	+3	–	–
	POX00353	hypothetical protein	NA	NA	NA	G->A	+8	–	–
	POX01000	hypothetical protein	NA	NA	NA	G->A	+1141	–	–
	POX01035	hypothetical protein	NA	NA	NA	A->T	+589	–	–
	POX01042	hypothetical protein	NA	NA	NA	G->C	+638	–	–
	POX01126	hypothetical protein	NA	NA	NA	G->T	+742	–	–
	POX01135	hypothetical protein	Winged helix repressor DNA-binding	NA	NA	C->T	+360	–	–
	POX01264	hypothetical protein	NA	NA	NA	G->A	+182	–	–
	POX01411	hypothetical protein	NA	NA	NA	C->A	+1233	–	–
	POX01440	hypothetical protein	NA	NA	NA	C->T	+76	–	–
	POX01469	hypothetical protein	NA	NA	NA	G->A	+266	–	–
	POX01631	hypothetical protein	NA	NA	NA	G->C	+30	–	–
	POX01770	hypothetical protein	NA	NA	NA	G->A	+196	–	–
	POX01829	hypothetical protein	NA	NA	NA	G->T	+1110	–	–
	POX02060	hypothetical protein	NA	NA	NA	G->A	+69	–	–
	POX02101	hypothetical protein	NA	NA	NA	C->T	+111	–	–
	POX02489	hypothetical protein	NA	NA	NA	G->A	+125	–	–
	POX02524	putative UDP-Glc: glycoprotein alpha-glucosyltransferase	NA	NA	GT24;GT8	G->T	+31	–	–
	POX02540	hypothetical protein	bZIP: Basic-leucine zipper (bZIP) transcription factor	NA	NA	C->T	+377	–	–
	POX02647	hypothetical protein	NA	NA	NA	C->T	+1054	–	–
	POX02742	hypothetical protein	NA	NA	NA	C->T	+40	–	–

	POX02758	hypothetical protein	NA	NA	NA	G->A	+224	–	–
	POX02798	hypothetical protein	NA	NA	NA	C->T	+241	–	–
	POX03586	hypothetical protein	Zn2Cys6: Fungal transcriptional regulatory protein, N-terminal	NA	NA	C->T	+798	–	–
	POX03920	hypothetical protein	NA	NA	NA	G->A	+380	–	–
	POX03999	hypothetical protein	Zn2Cys6: Fungal transcriptional regulatory protein, N-terminal	NA	NA	C->T	+1375	–	–
	POX04009	hypothetical protein	NA	NA	NA	G->T	+1139	–	–
	POX04573	hypothetical protein	NA	NA	NA	A->G	+23	–	–
	POX04595	hypothetical protein	YL1 nuclear protein	NA	NA	G->A	+51	–	–
	POX04640	hypothetical protein	NA	NA	NA	G->A	+128	–	–
	POX04658	hypothetical protein	NA	NA	NA	C->T	+736	–	–
	POX04693	hypothetical protein	NA	NA	NA	A->C	+52	–	–
	POX05197	hypothetical protein	NA	NA	NA	G->A	+1005	–	–
	POX05320	hypothetical protein	NA	NA	NA	G->A	+681	–	–
	POX05369	hypothetical protein	NA	NA	NA	G->A	+98	–	–
	POX05643	hypothetical protein	NA	NA	NA	T->G	+682	–	–
	POX05696	hypothetical protein	NA	NA	NA	C->T	+409	–	–
	POX06007	hypothetical protein	NA	NA	NA	C->T	+923	–	–
	POX06041	hypothetical protein	NA	NA	NA	C->T	+164	–	–
	POX06094	hypothetical protein	NA	NA	NA	G->T	+213	–	–
	POX06219	hypothetical protein	NA	NA	NA	C->T	+282	–	–
	POX06779	hypothetical protein	NA	NA	NA	G->T	+7	–	–
	POX06820	hypothetical protein	NA	NA	NA	G->T	+31	–	–
	POX07186	hypothetical protein	NA	NA	NA	G->A	+888	–	–

	POX07253	hypothetical protein	NA	NA	NA	C->A	+156	–	–
	POX07541	hypothetical protein	NA	NA	NA	C->T	+435	–	–
	POX07547	hypothetical protein	NA	NA	NA	G->T	+96	–	–
	POX07777	hypothetical protein	NA	NA	NA	G->A	+332	–	–
	POX08476	putative rhamnogalacturonan acetylesterase	NA	Pectin acetylesterase; rhamnogalacturonan acetylesterase	CE12	G->T	+599	–	–
	POX08835	hypothetical protein	NA	NA	NA	G->A	+521	–	–
	POX09251	hypothetical protein	NA	NA	NA	G->A	+490	–	–
	POX09827	hypothetical protein	NA	NA	NA	C->G	+849	–	–
**200-bp downstream of terminal codes**	POX00316	hypothetical protein	NA	NA	NA	G->A	-165	–	–
	POX02696	hypothetical protein	NA	NA	NA	C->G	-171	–	–
	POX02766	hypothetical protein	Zn2Cys6: Fungal transcriptional regulatory protein, N-terminal	NA	NA	G->A	-83	–	–
	POX03118	hypothetical protein	NA	NA	NA	G->A	-127	–	–
	POX03204	hypothetical protein	NA	NA	NA	G->A	-40	–	–
	POX03328	hypothetical protein	NA	NA	NA	G->T	-18	–	–
	POX04322	hypothetical protein	NA	NA	NA	C->T	-29	–	–
	POX04791	hypothetical protein	NA	NA	NA	G->A	-79	–	–
	POX05545	hypothetical protein	NA	NA	NA	G->T	-91	–	–
	POX06196	hypothetical protein	NA	NA	AA1	T->G	-141	–	–
	POX06244	hypothetical protein	NA	NA	NA	G->A	-120	–	–
	POX06605	hypothetical protein	NA	NA	NA	C->T	-162	–	–
	POX07209	hypothetical protein	NA	NA	NA	C->T	-44	–	–
	POX07172	hypothetical protein	NA	NA	NA	A->G	-28	–	–

	POX07291	hypothetical protein	NA	NA	NA	G->A	-45	–	–
	POX07678	hypothetical protein	NA	NA	NA	C->T	-124	–	–
**Others**	POX00007	beta-xylosidase	NA	beta-xylosidase/alpha-L-arabinofuranosidase	GH3; AA5	G->A	-562	–	–
	POX00011	hypothetical protein	NA	NA	NA	A->C	-997	–	–
	POX00424	hypothetical protein	NA	NA	NA	G->A	-1144	–	–
	POX00478	hypothetical protein	NA	NA	NA	G->A	-769	–	–
	POX00811	hypothetical protein	NA	NA	NA	C->T	-395	–	–
	POX00937	hypothetical protein	NA	NA	NA	C->T	+1735	–	–
	POX01380	hypothetical protein	NA	NA	NA	C->T	-510	–	–
	POX01599	hypothetical protein	NA	NA	NA	G->A	-148	–	–
	POX01754	hypothetical protein	NA	NA	NA	G->A	-308	–	–
	POX01834	hypothetical protein	NA	NA	NA	G->A	-1040	–	–
	POX02108	hypothetical protein	NA	NA	NA	G->A	-1891	–	–
	POX02134	hypothetical protein	NA	NA	NA	T->A	-6374	–	–
	POX02246	hypothetical protein	NA	NA	NA	G->A	-448	–	–
	POX02451	hypothetical protein	NA	NA	NA	A->G	-3660	–	–
	POX02527	hypothetical protein	NA	NA	NA	G->A	+1782	–	–
	POX02583	hypothetical protein	NA	NA	NA	C->T	-261	–	–
	POX02586	hypothetical protein	NA	NA	NA	G->T	-353	–	–
	POX02589	hypothetical protein	NA	NA	NA	G->T	-1030	–	–
	POX02942	hypothetical protein	NA	NA	NA	G->A	-349	–	–
	POX03078	hypothetical protein	NA	NA	NA	G->A	-1504	–	–
	POX03207	hypothetical protein	NA	NA	NA	G->T	-683	–	–
	POX03211	hypothetical protein	NA	NA	NA	G->A	-618	–	–

	POX03731	hypothetical protein	NA	NA	NA	C->A	-624	–	–
	POX03783	hypothetical protein	NA	NA	NA	C->T	-389	–	–
	POX03876	hypothetical protein	NA	NA	NA	T->C	-41599	–	–
	POX03876	hypothetical protein	NA	NA	NA	C->T	-1650	–	–
	POX03997	hypothetical protein	NA	NA	NA	C->A	-1320	–	–
	POX03998	hypothetical protein	NA	NA	NA	G->A	+1522	–	–
	POX04025	hypothetical protein	NA	NA	NA	G->C	+1712	–	–
	POX04036	hypothetical protein	NA	NA	NA	G->A	-1552	–	–
	POX04146	hypothetical protein	NA	NA	NA	G->A	-334	–	–
	POX04532	carbohydrate binding domain-containing protein	NA	NA	NA	C->T	-818	–	–
	POX04697	hypothetical protein	NA	NA	NA	G->C	-489	–	–
	POX04701	hypothetical protein	Zn2Cys6: Fungal transcriptional regulatory protein, N-terminal	NA	NA	G->A	+2261	–	–
	POX04880	hypothetical protein	NA	NA	NA	C->T	-991	–	–
	POX04908	hypothetical protein	NA	NA	NA	G->T	-224	–	–
	POX04956	hypothetical protein	NA	NA	NA	G->A	-203	–	–
	POX05036	hypothetical protein	NA	NA	NA	C->T	-1193	–	–
	POX05410	hypothetical protein	NA	NA	NA	G->A	-3033	–	–
	POX05516	hypothetical protein	NA	NA	NA	G->A	-349	–	–
	POX05758	hypothetical protein	NA	NA	NA	G->A	-673	–	–
	POX05813	hypothetical protein	NA	NA	NA	G->A	-553	–	–
	POX05922	hypothetical protein	NA	NA	NA	G->T	-239	–	–
	POX06015	hypothetical protein	NA	NA	NA	G->A	-1900	–	–
	POX06142	hypothetical protein	NA	NA	NA	G->A	-1047	–	–

	POX06224	hypothetical protein	NA	NA	NA	G->A	+1690	–	–
	POX06461	hypothetical protein	NA	NA	NA	G->A	-1477	–	–
	POX06556	hypothetical protein	NA	NA	NA	C->T	-779	–	–
	POX06559	hypothetical protein	NA	NA	NA	G->A	-246	–	–
	POX06663	hypothetical protein	NA	NA	NA	C->T	-771	–	–
	POX06773	hypothetical protein	NA	NA	NA	T->C	-648	–	–
	POX07198	hypothetical protein	NA	NA	NA	C->T	-342	–	–
	POX07212	hypothetical protein	NA	NA	NA	G->A	-2182	–	–
	POX07257	hypothetical protein	NA	NA	NA	C->T	-570	–	–
	POX07253	hypothetical protein	NA	NA	NA	A->C	-6221	–	–
	POX07253	hypothetical protein	NA	NA	NA	C->T	-7653	–	–
	POX07714	hypothetical protein	NA	NA	NA	A->T	+2005	–	–
	POX07758	hypothetical protein	NA	NA	NA	G->A	-849	–	–
	POX07868	hypothetical protein	NA	NA	NA	C->T	-467	–	–
	POX08234	hypothetical protein	NA	NA	NA	G->A	-453	–	–
	POX08278	hypothetical protein	NA	NA	NA	C->T	-211	–	–
	POX08736	hypothetical protein	NA	NA	NA	A->C	-9233	–	–
	POX08872	hypothetical protein	NA	NA	NA	G->A	-1157	–	–
**InDels**									
	**Gene ID**	**Functional annotation**	**Predicted transcription factor**	**CWDE**	**CAZy family**	**Insertion/deletion**	**Mutation nucleotides**	**Nucleoside position**	
**CDS**	POX01312	hypothetical protein	NA	NA	NA	Deletion	C	278	
**Intron**	POX05376	hypothetical protein	NA	NA	NA	Deletion	C	289	
**Intergenic genes**	POX09781	hypothetical protein	NA	NA	NA	Deletion	TAAT	+123	
	POX07939	hypothetical protein	NA	NA	NA	Deletion	A	+1462	

	POX09804	hypothetical protein	NA	NA	NA	Deletion	T	+151
	POX02071	hypothetical protein	Zn2Cys6: Fungal specific transcription factor	NA	NA	Deletion	CG	-1624
	POX09745	hypothetical protein	NA	NA	NA	Deletion	TAC	+3971
	POX07713	hypothetical protein	NA	NA	NA	Deletion	A	+20762
	POX07713	hypothetical protein	NA	NA	NA	Insertion	TT	-7097
	POX06050	hypothetical protein	NA	NA	NA	Deletion	A	-747
	POX01922	hypothetical protein	NA	NA	NA	Deletion	C	-843
	POX08605	hypothetical protein	NA	NA	NA	Deletion	GCCAG	-912

'+' and '-' respectively represent the upstream of start codon and downstream of stop codon. CDS, coding DNA sequence; CAZy, Carbohydrate-Active Enzymes; GH, Glycoside hydrolase; CE, Carbohydrate esterase; GT, Glycosyl transferase; AA, Auxiliary activities; CWDE, Plant cell wall degrading enzyme
