# Supplementary material for: Comparative genomic, transcriptomic and secretomic profiling of Penicillium oxalicum HP7-1 and its cellulase and xylanase hyper-producing mutant EU2106, and identification of two novel regulatory genes of cellulase and xylanase gene expression
Source: Biotechnol Biofuels. 2016 Sep 23;9:203. doi: 10.1186/s13068-016-0616-9 (PMC5035457; doi:10.1186/s13068-016-0616-9)
Supplement: Supplementary file 3 — 10.1186/s13068-016-0616-9 List of 347 genes displaying altered expression in the cellulase and xylanase hyper-producing mutant P. oxalicum strain EU2106 compared with the wild-type strain HP7-1 when cultivated in medium containing wheat bran and Avicel as the carbon source. [file 13068_2016_616_MOESM3_ESM.pdf]

Additional file 3: Table S2. List of the 347 genes whose expression was altered in the cellulase hyper-producing mutant *P. oxalicum* strain EU2106 compared with the wild-type strain HP7-1 when cultivated in medium containing wheat bran and Avicel as the carbon source

Gene ID	Function annotation	CAZy family	CWDE	IPR prediction	FPKM in HP7-1	FPKM in EU2106	Log2 (EU2106 /HP7-1)	Regulation in EU2106	Probability
POX01796	beta-1, 4-N-acetylgalactosaminyltransferase	GT25	NA	IPR002654; Glycosyl transferase, family 25	21.2967	113.397	2.412678688	Up	0.849973305
POX06079	beta-glucosidase	GH1	beta-glucosidase	IPR001360; Glycoside hydrolase, family 1	1975.47	7435.5	1.912230741	Up	0.878092187
POX03641	beta-glucosidase	GH3	beta-glucosidase	IPR001764; Glycoside hydrolase, family 3	475.083	1242.64	1.387159769	Up	0.822975618
POX01961	beta-mannosidase	GH2	beta-mannosidase	IPR006102; Glycoside hydrolase, family 2, immunoglobulin-like beta-sandwich	280.567	772.76	1.461676802	Up	0.829079907
POX02490	cellobiohydrolase	GH7	cellobiohydrolase	IPR001722; Glycoside hydrolase, family 7	101.83	566.413	2.475692572	Up	0.898202527
POX06051	cellodextrin transporter cdt-c	NA	NA	IPR003663; Sugar/inositol transporter// IPR020846; Major facilitator superfamily domain	4601.59	12781.3	1.47383182	Up	0.836625734
POX05188	chitin glucanosyltransferase	GH16	NA	IPR000757; Glycoside hydrolase, family 16	0.84333	52.23	5.952631914	Up	0.851877558
POX07971	chitinase	GH18	NA	IPR001223; Glycoside hydrolase family 18, catalytic domain	80.0433	259.09	1.69460018	Up	0.837515572
POX05570	endo-beta-1,4-glucanase	CBM1; GH45	endo-beta-1,4-glucanase	IPR000254; Cellulose-binding domain, fungal// IPR000334; Glycoside hydrolase, family 45	3359.26	8017.75	1.255054804	Up	0.808097526
POX05571	endo-beta-1,4-glucanase 1	CBM1; GH7	endo-beta-1,4-glucanase	IPR000254; Cellulose-binding domain, fungal// IPR001722; Glycoside hydrolase, family 7	1616.75	4859.37	1.58767069	Up	0.850631785
POX00065	hypothetical protein	NA	NA	IPR001804; Isocitrate and isopropylmalate dehydrogenases family	52.5933	204.217	1.957148771	Up	0.850685175
POX00146	hypothetical protein	NA	NA	IPR001279; Metallo-beta-lactamase	61.3467	185.573	1.596932551	Up	0.818473038
POX00169	hypothetical protein	NA	NA	IPR018825; Domain of unknown function DUF2427	65.4767	422.57	2.690137565	Up	0.901192383
POX00252	hypothetical protein	NA	NA	IPR006254; Isocitrate lyase	115.593	545.987	2.239807525	Up	0.888538886
POX00292	hypothetical protein	NA	NA	IPR005631; Flavinator of succinate dehydrogenase	38.8133	128.72	1.729611987	Up	0.816408614

POX00301	hypothetical protein	NA	NA	IPR008146; Glutamine synthetase, catalytic domain	286.52	1135.43	1.986526778	Up	0.87933796
POX00441	hypothetical protein	NA	NA	IPR000146; Fructose-1,6-bisphosphatase class 1	50.47	252.923	2.325202147	Up	0.877237943
POX00445	hypothetical protein	NA	NA	NA	225.263	1394.99	2.630566926	Up	0.909788219
POX00659	hypothetical protein	NA	NA	IPR002327; Cytochrome c, class IA/ IB	620.397	2073.84	1.741041752	Up	0.864655633
POX00667	hypothetical protein	NA	NA	IPR006931; Calcipressin	65.8767	200.633	1.606721854	Up	0.821605268
POX00769	hypothetical protein	NA	NA	IPR002872; Proline dehydrogenase domain	23.41	92.6567	1.984769848	Up	0.816550988
POX00805	hypothetical protein	NA	NA	IPR002680; Alternative oxidase	73.9	368.1	2.31645148	Up	0.885566827
POX00860	hypothetical protein	NA	NA	NA	92.07	246.95	1.423415917	Up	0.805178857
POX01067	hypothetical protein	NA	NA	IPR015767; Rho GTPase activating protein	279.877	1066.44	1.92993517	Up	0.875191315
POX01075	hypothetical protein	NA	NA	IPR002020; Citrate synthase	676.687	1794.05	1.406657551	Up	0.826784125
POX01102	hypothetical protein	NA	NA	IPR002048; EF-hand domain	30.9033	121.69	1.977376253	Up	0.831393486
POX01238	hypothetical protein	NA	NA	IPR002085; Alcohol dehydrogenase superfamily, zinc-type	1073.32	4502.84	2.068760498	Up	0.887435487
POX01283	hypothetical protein	NA	NA	IPR006091; Acyl-CoA oxidase/dehydrogenase, central domain	178.987	653.693	1.868761864	Up	0.867609895
POX01420	hypothetical protein	NA	NA	IPR031833; Protein of unknown function DUF4748	122.687	332.907	1.440139293	Up	0.814878092
POX01435	hypothetical protein	NA	NA	IPR002198; Short-chain dehydrogenase/reductase SDR	18.07	81.66	2.176033061	Up	0.820074746
POX01617	hypothetical protein	NA	NA	IPR002198; Short-chain dehydrogenase/reductase SDR	36.4467	167.293	2.198521175	Up	0.858302189
POX01660	hypothetical protein	NA	NA	IPR008030; NmrA-like domain//IPR016040; NAD(P)-binding domain	24.6167	96.8667	1.976364877	Up	0.819256095
POX01761	hypothetical protein	NA	NA	IPR004136; Nitronate monooxygenase	76.29	254.65	1.738949849	Up	0.841751201
POX01812	hypothetical protein	NA	NA	IPR011701; Major facilitator superfamily	0.91	3334.81	11.83944896	Up	0.998665243
POX01840	hypothetical protein	NA	NA	IPR018713; Domain of unknown function DUF2236	224.093	605.787	1.434710095	Up	0.823135789
POX01981	hypothetical protein	NA	NA	IPR020846; Major facilitator superfamily domain	35.5467	142.323	2.00138602	Up	0.840434241
POX02134	hypothetical protein	NA	NA	IPR000103; Pyridine nucleotide-disulphide oxidoreductase, class-II	3.21	54.5333	4.08649314	Up	0.835362164
POX02199	hypothetical protein	NA	NA	IPR003736; Phenylacetic acid degradation-related domain	29.74	105.963	1.83308858	Up	0.814326393

POX02214	hypothetical protein	NA	NA	IPR006108; 3-hydroxyacyl-CoA dehydrogenase, C-terminal	66.03	211.843	1.681804177	Up	0.830895177
POX02301	hypothetical protein	NA	NA	IPR000634; Serine/threonine dehydratase, pyridoxal-phosphate-binding site	16.9667	99.5933	2.553346018	Up	0.848371596
POX02628	hypothetical protein	NA	NA	IPR001563; Peptidase S10, serine carboxypeptidase	233.533	1329.36	2.509031457	Up	0.906086492
POX02795	hypothetical protein	NA	NA	IPR027417; P-loop containing nucleoside triphosphate hydrolase	51.8567	204.26	1.977805332	Up	0.851966542
POX02896	hypothetical protein	NA	NA	IPR004045; Glutathione S-transferase, N-terminal	321.293	842.98	1.391607357	Up	0.820982381
POX02962	hypothetical protein	NA	NA	IPR005995; Phosphoglycerate mutase, 2,3-bisphosphoglycerate-independent	91.9433	264.023	1.521848559	Up	0.821445097
POX03007	hypothetical protein	NA	NA	IPR018620; Ubiquitin 3 binding protein But2, C-terminal	19.9567	78.9933	1.984860132	Up	0.806122086
POX03048	hypothetical protein	NA	NA	IPR011766; Thiamine pyrophosphate enzyme, C-terminal TPP-binding	167.853	862.483	2.361295391	Up	0.897437266
POX03111	hypothetical protein	NA	NA	IPR007698; Alanine dehydrogenase/pyridine nucleotide transhydrogenase, NAD(H)-binding domain	40.1933	128.713	1.679133375	Up	0.81197722
POX03113	hypothetical protein	NA	NA	IPR000771; Ketose-bisphosphate aldolase, class-II	700.62	2280.45	1.70261657	Up	0.860811532
POX03202	hypothetical protein	NA	NA	IPR016181; Acyl-CoA N-acyltransferase	69.55	275.983	1.988458725	Up	0.860811532
POX03214	hypothetical protein	NA	NA	IPR007859; Electron transfer flavoprotein-ubiquinone oxidoreductase	74.4467	212.3	1.511825212	Up	0.813543335
POX03248	hypothetical protein	NA	NA	IPR008253; Marvel domain	167.757	516.167	1.621466877	Up	0.843281723
POX03300	hypothetical protein	NA	NA	NA	53.04	232.127	2.129759588	Up	0.864548852
POX03389	hypothetical protein	NA	NA	NA	54.5433	3102.55	5.829906229	Up	0.957412351
POX03665	hypothetical protein	NA	NA	IPR002067; Mitochondrial carrier protein	98.9	260.293	1.396095936	Up	0.804182239
POX03833	hypothetical protein	NA	NA	IPR004299; Membrane bound O-acyl transferase, MBOAT	57.9233	196.71	1.763853768	Up	0.835896067
POX03836	hypothetical protein	NA	NA	NA	54.75	321.957	2.555935654	Up	0.891617726
POX03937	hypothetical protein	NA	NA	IPR000796; Aspartate/other aminotransferase	264.257	1232.73	2.221848974	Up	0.893450792
POX03950	hypothetical protein	NA	NA	IPR001753; Crotonase superfamily	75.74	222.04	1.551692272	Up	0.819113721

POX03973	hypothetical protein	NA	NA	IPR021838; Protein of unknown function DUF3431	15.5767	82.5567	2.405998185	Up	0.83087738
POX04022	hypothetical protein	NA	NA	NA	171.513	454.673	1.406509657	Up	0.816141662
POX04289	hypothetical protein	NA	NA	IPR001753; Crotonase superfamily	62.11	244.143	1.974830911	Up	0.856504716
POX04352	hypothetical protein	NA	NA	IPR028116; Putative oxalocrotonate tautomerase	1.48667	40.22	4.757759967	Up	0.813009432
POX04356	hypothetical protein	NA	NA	IPR002020; Citrate synthase	113.13	434.103	1.940056944	Up	0.867325147
POX04391	hypothetical protein	NA	NA	IPR006254; Isocitrate lyase	74.2433	584.68	2.977313852	Up	0.913632319
POX04450	hypothetical protein	NA	NA	IPR000362; Fumarate lyase family	150.91	388.56	1.364448983	Up	0.807172095
POX04452	hypothetical protein	NA	NA	IPR000796; Aspartate/other aminotransferase	33.7167	148.133	2.135362497	Up	0.850791956
POX04462	hypothetical protein	NA	NA	IPR012336; Thioredoxin-like fold	96.7433	639.35	2.724371771	Up	0.907456843
POX04515	hypothetical protein	NA	NA	NA	73.1667	477.817	2.707198738	Up	0.903523759
POX04526	hypothetical protein	NA	NA	IPR002085; Alcohol dehydrogenase superfamily, zinc-type	7.88	86.09	3.449578133	Up	0.861060687
POX04527	hypothetical protein	NA	NA	IPR012336; Thioredoxin-like fold	281.017	789.71	1.490667262	Up	0.833440114
POX04768	hypothetical protein	NA	NA	NA	28.97	113.743	1.9731504	Up	0.827424809
POX04781	hypothetical protein	NA	NA	NA	417.137	1003.94	1.267085803	Up	0.805552589
POX04819	hypothetical protein	NA	NA	IPR001576; Phosphoglycerate kinase	206.377	584.017	1.500729675	Up	0.832550276
POX04838	hypothetical protein	NA	NA	IPR001753; Crotonase superfamily	21.38	132.15	2.627842667	Up	0.865474284
POX04991	hypothetical protein	NA	NA	IPR000652; Triosephosphate isomerase	328.23	1032.54	1.653423318	Up	0.852820787
POX05087	hypothetical protein	NA	NA	IPR014756; Immunoglobulin E-set	48.0833	149.17	1.633348602	Up	0.813258587
POX05105	hypothetical protein	NA	NA	IPR025187; Protein of unknown function DUF4112	42.3967	140.05	1.723919237	Up	0.819736608
POX05155	hypothetical protein	NA	NA	IPR011701; Major facilitator superfamily	35.7367	134.49	1.912021927	Up	0.832123154
POX05260	hypothetical protein	AA7	NA	IPR006094; FAD linked oxidase, N-terminal	43.7333	181	2.049184478	Up	0.851948745
POX05321	hypothetical protein	NA	NA	IPR002198; Short-chain dehydrogenase/reductase SDR	34.16	186.83	2.451346253	Up	0.87236163
POX05482	hypothetical protein	NA	NA	IPR000454; ATPase, F0 complex, subunit C	57.9533	589.433	3.3463651	Up	0.920786617
POX05492	hypothetical protein	NA	NA	IPR002067; Mitochondrial carrier protein	23.11	101.723	2.138061478	Up	0.831571454
POX05495	hypothetical protein	NA	NA	IPR000073; Alpha/beta hydrolase fold-1	28.5967	108.253	1.920492553	Up	0.821498487

POX05577	hypothetical protein	NA	NA	NA	181.203	491.683	1.440119958	Up	0.821658658
POX05586	hypothetical protein	NA	NA	IPR002376; Formyl transferase, N-terminal	49.6667	234.197	2.237370712	Up	0.871276028
POX05593	hypothetical protein	NA	NA	IPR008030; NmrA-like domain// IPR016040; NAD(P)-binding domain	51.3733	179.7	1.806498818	Up	0.836501157
POX05697	hypothetical protein	NA	NA	IPR015590; Aldehyde dehydrogenase domain	42.9333	150.777	1.81224309	Up	0.830147713
POX05796	hypothetical protein	NA	NA	IPR001841; Zinc finger, RING-type//	70.35	246.543	1.809218914	Up	0.846075814
POX05956	hypothetical protein	NA	NA	IPR007667; Hypoxia induced protein, domain	131.697	350.567	1.412469995	Up	0.811710269
POX05960	hypothetical protein	NA	NA	IPR002155; Thiolase	135.39	360.52	1.412958111	Up	0.812244172
POX06017	hypothetical protein	NA	NA	IPR006683; Thioesterase superfamily	43.52	152.39	1.808017773	Up	0.830485852
POX06020	hypothetical protein	NA	NA	IPR005593; Xylulose 5-phosphate/Fructose 6-phosphate phosphoketolase	534.477	1263.46	1.241184925	Up	0.80181527
POX06092	hypothetical protein	NA	NA	IPR009686; Senescence/spartin-associated	286.073	709.04	1.309481997	Up	0.808453461
POX06117	hypothetical protein	NA	NA	IPR006089; Acyl-CoA dehydrogenase, conserved site	114.563	330.543	1.528694043	Up	0.827211248
POX06121	hypothetical protein	NA	NA	NA	77.8833	239.94	1.623287151	Up	0.828029899
POX06204	hypothetical protein	NA	NA	IPR002067; Mitochondrial carrier protein	392.227	1050.53	1.421357832	Up	0.82576971
POX06223	hypothetical protein	NA	NA	NA	8.38667	51.3367	2.613820202	Up	0.800444919
POX06224	hypothetical protein	NA	NA	IPR002293; Amino acid/polyamine transporter I	58.18	229.287	1.978557261	Up	0.855632675
POX06283	hypothetical protein	NA	NA	IPR011701; Major facilitator superfamily	0.31667	79.7233	7.975893209	Up	0.912867058
POX06361	hypothetical protein	NA	NA	IPR001917; Aminotransferase, class-II, pyridoxal-phosphate binding site	46.0433	197.413	2.100155246	Up	0.858035238
POX06446	hypothetical protein	NA	NA	IPR002068; Alpha crystallin/Hsp20 domain	50.6167	195.84	1.951991055	Up	0.848709735
POX06621	hypothetical protein	NA	NA	IPR009027; Ribosomal protein L9/RNase H1, N-terminal	307.687	802.453	1.382955568	Up	0.819220502
POX06646	hypothetical protein	NA	NA	IPR000941; Enolase	639.71	1584.4	1.308449701	Up	0.812991636
POX06699	hypothetical protein	NA	NA	IPR000634; Serine/threonine dehydratase, pyridoxal-phosphate-binding site	26.83	136.81	2.350254727	Up	0.857269977

POX06725	hypothetical protein	NA	NA	IPR002539; MaoC-like domain	21.8467	121.6	2.476658152	Up	0.85654031
POX06753	hypothetical protein	NA	NA	IPR008816; Glycine zipper 2TM domain	1015.94	3819.25	1.910468165	Up	0.877843033
POX06754	hypothetical protein	NA	NA	NA	7.91	129.647	4.034763609	Up	0.891475351
POX06848	hypothetical protein	NA	NA	IPR004045; Glutathione S-transferase, N-terminal	50.58	213.7	2.078947966	Up	0.859103043
POX06874	hypothetical protein	NA	NA	IPR004136; Nitronate monooxygenase	9.8	808.437	6.366209197	Up	0.9571454
POX06884	hypothetical protein	NA	NA	IPR002067; Mitochondrial carrier protein	2660.28	6764.43	1.34638956	Up	0.82071543
POX06901	hypothetical protein	NA	NA	IPR012292; Globin/Protoglobin	125.167	433.273	1.791427039	Up	0.856682684
POX06915	hypothetical protein	NA	NA	IPR011701; Major facilitator superfamily	68.2133	217.493	1.672845511	Up	0.831090941
POX06926	hypothetical protein	NA	NA	NA	689.52	1694.14	1.296891636	Up	0.811906033
POX07055	hypothetical protein	NA	NA	IPR016039; Thiolase-like	18.29	188.623	3.366381173	Up	0.896956754
POX07072	hypothetical protein	NA	NA	IPR011893; Selenoprotein, Rdx type	42.3767	130.133	1.618648537	Up	0.80565937
POX07177	hypothetical protein	NA	NA	IPR003736; Phenylacetic acid degradation-related domain	26.1733	95.88	1.873132237	Up	0.811639082
POX07255	hypothetical protein	NA	NA	NA	107.753	282.657	1.391318225	Up	0.80500089
POX07291	hypothetical protein	NA	NA	IPR018108; Mitochondrial substrate/solute carrier	83.6433	759.52	3.182765493	Up	0.920181527
POX07339	hypothetical protein	NA	NA	IPR009009; RlpA-like double-psi beta-barrel domain	16.0433	146.547	3.191314325	Up	0.884089696
POX07364	hypothetical protein	NA	NA	IPR001214; SET domain//IPR003616; Post-SET domain	12.8267	73.6767	2.522061493	Up	0.827798541
POX07393	hypothetical protein	NA	NA	IPR003439; ABC transporter-like//IPR013103	20.85	87.89	2.075651643	Up	0.818633209
POX07416	hypothetical protein	NA	NA	IPR020846; Major facilitator superfamily domain	23.0933	92.4167	2.000676625	Up	0.817440826
POX07419	hypothetical protein	NA	NA	IPR006913; Glutathione-dependent formaldehyde-activating enzyme/centromere protein V	17.6367	73.4033	2.057267662	Up	0.804555971
POX07456	hypothetical protein	NA	NA	IPR002048; EF-hand domain	51.0933	157.793	1.626829288	Up	0.815518776
POX07795	hypothetical protein	NA	NA	IPR009291; Vacuolar protein sorting-associated protein 62	27.7333	104.77	1.91753274	Up	0.819683218
POX07938	hypothetical protein	NA	NA	IPR001138; Zn(2)-C6 fungal-type DNA-binding domain	13.6367	201.973	3.888601884	Up	0.90560598
POX07996	hypothetical protein	NA	NA	IPR007138; Antibiotic biosynthesis monooxygenase domain	34.5533	183.593	2.409616874	Up	0.870297206
POX08125	hypothetical protein	NA	NA	IPR002085; Alcohol dehydrogenase superfamily, zinc-type	36.9267	120.953	1.711715586	Up	0.811443317

POX08455	hypothetical protein	NA	NA	IPR000898; Indoleamine 2,3-dioxygenase	102.567	268.503	1.388378056	Up	0.803826304
POX08459	hypothetical protein	NA	NA	IPR001452; SH3 domain	69.1967	201.773	1.543961071	Up	0.815607759
POX08478	hypothetical protein	NA	NA	IPR002068; Alpha crystallin/Hsp20 domain	588.997	3069.26	2.381561055	Up	0.903470368
POX08659	hypothetical protein	NA	NA	IPR002655; Acyl-CoA oxidase, C-terminal	8.02667	73.8933	3.202571319	Up	0.845862253
POX08794	hypothetical protein	NA	NA	IPR017926; Glutamine amidotransferase	30.62	146.283	2.256219219	Up	0.857092009
POX08821	hypothetical protein	NA	NA	NA	227.2	860.193	1.920698115	Up	0.873233671
POX08822	hypothetical protein	NA	NA	IPR000971; Globin	393.447	2591.82	2.719727392	Up	0.914984873
POX08943	hypothetical protein	NA	NA	IPR001260; Coproporphyrinogen III oxidase, aerobic	60.9267	182.823	1.585304493	Up	0.817120484
POX08956	hypothetical protein	NA	NA	IPR002489; Glutamate synthase, alpha subunit, C-terminal	45.6567	133.833	1.551540048	Up	0.800800854
POX08961	hypothetical protein	NA	NA	IPR031342; Meiotically up-regulated gene 163 protein/ YGR053C	41.3033	125.583	1.604314889	Up	0.802438156
POX09344	hypothetical protein	NA	NA	IPR006091; Acyl-CoA oxidase/dehydrogenase, central domain	35.2667	111.683	1.66303678	Up	0.803363588
POX09345	hypothetical protein	NA	NA	IPR018809; Protein of unknown function DUF2406	240.257	600.147	1.320738653	Up	0.808115323
POX09632	hypothetical protein	NA	NA	IPR011701; Major facilitator superfamily	16.3933	194.83	3.571034702	Up	0.9005517
POX09827	hypothetical protein	NA	NA	NA	39.5267	162.437	2.038979126	Up	0.847962271
POX01218	acetyl xylan esterase	CBM1; CE1	Acetyl xylan esterase	IPR000254; Cellulose-binding domain, fungal// IPR010126; Esterase, PHB depolymerase	580.17	112.363	-2.368304368	Down	0.894571988
POX02710	alpha-1, 3-glucanase	GH71	NA	IPR005197; Glycoside hydrolase family 71	449.683	60.1167	-2.903072492	Down	0.908079729
POX09352	alpha-amylase Amy13A	CBM20; GH13	NA	IPR002044; Carbohydrate binding module family 20// IPR006047; Glycosyl hydrolase, family 13	1305.54	546.347	-1.256758098	Down	0.805018687
POX09085	alpha-galactosidase	CBM1; GH27; GH36	alpha-galactosidase	IPR000254; Cellulose-binding domain, fungal// IPR002241; Glycoside hydrolase, family 27	892.003	273.363	-1.706229356	Down	0.857536928
POX03741	alpha-glucosidase	GH13	NA	IPR006047; Glycosyl hydrolase, family 13, catalytic domain	211.83	54.7767	-1.951273541	Down	0.851308062
POX06751	alpha-glucosidase	GH31	NA	IPR000322; Glycoside hydrolase family 31	185.063	27.3333	-2.759285768	Down	0.882434597

POX01921	alpha-L-arabinofuranosidase	GH43	beta-xylosidase/ alpha-L-arabinofuranosidase	IPR006710; Glycoside hydrolase, family 43	82.4033	10.4167	-2.983809009	Down	0.849261434
POX05240	alpha-L-arabinofuranosidase	GH117; GH43	beta-xylosidase/ alpha-L-arabinofuranosidase	IPR006710; Glycoside hydrolase, family 43	47.82	3.47	-3.784606562	Down	0.819434063
POX05540	alpha-L-arabinofuranosidase	GH62	beta-xylosidase/ alpha-L-arabinofuranosidase	IPR005193; Glycoside hydrolase, family 62, arabinosidase	107.867	10.2867	-3.390401641	Down	0.873500623
POX06789	alpha-L-rhamnosidase	GH78	NA	IPR008902; Bacterial alpha-L-rhamnosidase	85.89	17.4633	-2.29816121	Down	0.829596014
POX00053	alpha-mannosidase	GH92	NA	IPR005887; Alpha-1,2-mannosidase, putative	91.6367	6.57333	-3.80122793	Down	0.869567539
POX01189	alpha-mannosidase	GH47	NA	IPR001382; Glycoside hydrolase family 47	630.523	159.723	-1.980974675	Down	0.874426054
POX06301	alpha-mannosidase	GH47	NA	IPR001382; Glycoside hydrolase family 47	302.357	80.6633	-1.906266459	Down	0.857999644
POX04559	beta-1,3-galactosyltransferase	NA	NA	IPR003378; Fringe-like//IPR003609; PAN/Apple domain	114.05	14.18	-3.007737009	Down	0.869425165
POX07534	beta-1,3-glucanosyltransferase	GH17	NA	IPR013781; Glycoside hydrolase, catalytic domain	4980.43	1738.51	-1.518416175	Down	0.84411817
POX04626	beta-1,3-glucanosyltransglycosylase	CBM43; GH72	NA	IPR004886; Glucanosyltransferase	569.86	221.657	-1.362280775	Down	0.8131874
POX08390	beta-1,6-glucanase	GH30	NA	IPR001139; Glycoside hydrolase, family 30	277.17	52.3733	-2.403866779	Down	0.882238833
POX09120	beta-glucanase	GH16	NA	IPR000757; Glycoside hydrolase, family 16	145.49	32.3733	-2.168042169	Down	0.852019932
POX06835	beta-glucosidase BGL1	GH3	beta-glucosidase	IPR001764; Glycoside hydrolase, family 3, N-terminal	442.93	146.977	-1.591491578	Down	0.837978288
POX06900	beta-glucuronidase	GH2	NA	IPR006102; Glycoside hydrolase, family 2, immunoglobulin-like beta-sandwich	88.45	25.3533	-1.802686705	Down	0.801370351
POX08337	beta-glucuronidase	GH79	NA	IPR013781; Glycoside hydrolase, catalytic domain	492.427	44.5133	-3.467599446	Down	0.920519665
POX02591	carbohydrate acetylesterase	CE16	NA	IPR001087; GDSL lipase/esterase	96.4667	23.96	-2.009402608	Down	0.820733227
POX05968	cellulose monooxygenase	AA9	cellulose monooxygenase	IPR005103; Glycoside hydrolase, family 61	122.66	23.2933	-2.396675843	Down	0.854084357

POX02308	cellulose monooxygenase Cel61A	AA9	cellulose monooxygenase	IPR005103; Glycoside hydrolase, family 61	10021.9	3847.04	-1.381339151	Down	0.824968856
POX07423	chitin binding domain- and peptidoglycan binding domain-containing protein	CBM18	NA	IPR001002; Chitin-binding, type 1	265.84	68.94	-1.947144994	Down	0.857287774
POX01381	chitin binding domain-containing protein	AA11	NA	NA	475.033	169.727	-1.484815499	Down	0.826232426
POX00089	chitinase	GH18	NA	IPR001223; Glycoside hydrolase family 18, catalytic domain	6795.32	1209.49	-2.490138674	Down	0.908382274
POX07424	chitinase	GH18	NA	IPR001223; Glycoside hydrolase family 18, catalytic domain	223.447	54.1267	-2.045519074	Down	0.858675921
POX09387	chitinase	GH18	NA	IPR001223; Glycoside hydrolase family 18, catalytic domain	2003.3	507.343	-1.98134419	Down	0.881188824
POX08903	chitosanase	GH75	NA	IPR009939; Fungal chitosanase	269.643	17.97	-3.907390051	Down	0.914201815
POX07378	cutinase	CE5	NA	IPR000675; Cutinase	2223.22	243.943	-3.188032756	Down	0.926054458
POX03738	endo-alpha-1, 4-polygalactosaminidase	GH114	NA	IPR004352; Glycoside-hydrolase family GH114, TIM-barrel domain	517.857	131.133	-1.981518384	Down	0.872201459
POX07535	endo-beta-1, 4-glucanase	GH12	endo-beta-1,4-glucanase	IPR002594; Glycoside hydrolase, family 12	387.733	26.97	-3.845637342	Down	0.921534081
POX05916	endo-beta-1, 4-xylanase	GH10	endo-beta-1,4-xylanase	IPR001000; Glycoside hydrolase, family 10	298.08	51.2467	-2.540169507	Down	0.889268553
POX03730	endopolygalacturonase	GH28	polygalacturonase; rhamnogalacturonase	IPR000743; Glycoside hydrolase, family 28	380.173	22.64	-4.069711477	Down	0.923918847
POX01219	exo-beta-1, 3-galactanase	CBM35; GH43	NA	IPR005084; Carbohydrate binding module family 6// IPR006710; Glycoside hydrolase, family 43	170.113	20.8067	-3.031378461	Down	0.886545649
POX09801	exo-beta-1, 3-galactanase	GH43	NA	IPR006710; Glycoside hydrolase, family 43	97.9867	20.23	-2.276089131	Down	0.837124043
POX00105	exo-beta-1, 3-glucanase	GH55	NA	IPR011050; Pectin lyase fold/virulence factor	284.957	67.9	-2.069259066	Down	0.866168357
POX06324	exo-beta-1, 3-glucanase	GH55	NA	IPR011050; Pectin lyase fold/virulence factor	105.913	24.13	-2.1339844	Down	0.832817227
POX01117	feruloyl esterase	NA	Feruloyl esterase	IPR011118; Tannase/feruloyl esterase	89.8833	18.0967	-2.312329645	Down	0.833262146

POX06393	histone H3	NA	NA	IPR000164; Histone H3/CENP-A	3842.3	1581.43	-1.28074172	Down	0.810873821
POX06394	histone H4	NA	NA	IPR001951; Histone H4	1767.28	615.217	-1.522361418	Down	0.842427478
POX00044	hypothetical protein	NA	NA	IPR008427; Extracellular membrane protein, CFEM domain	533.44	213.687	-1.319829118	Down	0.806709379
POX00093	hypothetical protein	NA	NA	NA	853.987	351.9	-1.279048031	Down	0.806015305
POX00243	hypothetical protein	NA	NA	IPR009311; Interferon alpha-inducible protein 6/27	64.9467	2.27	-4.83849128	Down	0.857875067
POX00244	hypothetical protein	NA	NA	NA	90.7333	4.18667	-4.437758697	Down	0.876686243
POX00338	hypothetical protein	NA	NA	IPR021851; Protein of unknown function DUF3455	124.483	4.03333	-4.947836146	Down	0.899750845
POX00388	hypothetical protein	NA	NA	IPR026777; Plasma membrane fusion protein PRM1	144.023	24.5333	-2.553487392	Down	0.867609895
POX00429	hypothetical protein	NA	NA	IPR011701; Major facilitator superfamily	385.403	128.447	-1.585199598	Down	0.835611319
POX00449	hypothetical protein	NA	NA	IPR001849; Pleckstrin homology domain	1304.15	389.807	-1.742282832	Down	0.863053924
POX00453	hypothetical protein	NA	NA	IPR006076; FAD dependent oxidoreductase	120.013	25.0333	-2.261272384	Down	0.847357181
POX00610	hypothetical protein	NA	NA	NA	1034.88	384.143	-1.429746867	Down	0.827264638
POX00775	hypothetical protein	NA	NA	IPR031606; Potassium transporter Kch	136.51	44.45	-1.618751315	Down	0.807972949
POX00795	hypothetical protein	NA	NA	IPR002129; Pyridoxal phosphate-dependent decarboxylase	358.273	135.21	-1.40585881	Down	0.812048407
POX00837	hypothetical protein	NA	NA	IPR011701; Major facilitator superfamily	492.613	115.373	-2.09414587	Down	0.878270155
POX00961	hypothetical protein	NA	NA	IPR004160; Translation elongation factor EFTu/EF1A, C-terminal	147.32	49.8967	-1.561937957	Down	0.80621107
POX01184	hypothetical protein	NA	NA	NA	66.2433	7.90333	-3.067242108	Down	0.835397758
POX01188	hypothetical protein	NA	NA	NA	195.323	15.6733	-3.639480361	Down	0.901548318
POX01193	hypothetical protein	NA	NA	NA	62.5333	3.45667	-4.177172029	Down	0.847962271
POX01201	hypothetical protein	NA	NA	IPR005828; Major facilitator, sugar transporter-like// IPR020846; Major facilitator superfamily domain	483.62	165.563	-1.546490708	Down	0.835860473
POX01460	hypothetical protein	NA	NA	IPR001251; CRAL-TRIO lipid binding domain	101.28	29.26	-1.791347635	Down	0.809165332
POX01614	hypothetical protein	NA	NA	NA	104.43	25.5733	-2.029824095	Down	0.826143442
POX01636	hypothetical protein	NA	NA	IPR002110; Ankyrin repeat	469.25	177.727	-1.400696581	Down	0.815643353

POX01704	hypothetical protein	NA	NA	NA	280.253	29.3367	-3.255954671	Down	0.905944118
POX01763	hypothetical protein	NA	NA	NA	371.407	101.657	-1.869294881	Down	0.859494572
POX01764	hypothetical protein	NA	NA	IPR001338; Hydrophobin	706.293	93.31	-2.920163871	Down	0.913863677
POX01797	hypothetical protein	NA	NA	IPR018535; Domain of unknown function DUF1996	123.273	38.9867	-1.660808032	Down	0.808400071
POX01798	hypothetical protein	NA	NA	IPR001395; Aldo/keto reductase/potassium channel subunit beta	610.03	190.25	-1.680983739	Down	0.852073323
POX01825	hypothetical protein	GT90	NA	IPR006598; Lipopolysaccharide-modifying protein	99.8	14.1133	-2.821981047	Down	0.856700481
POX01875	hypothetical protein	NA	NA	IPR002921; Fungal lipase-like domain	219.563	69.3133	-1.663432342	Down	0.830396868
POX01918	hypothetical protein	NA	NA	IPR013057; Amino acid transporter, transmembrane domain	140.163	38.4967	-1.864303553	Down	0.830788397
POX01929	hypothetical protein	NA	NA	IPR001563; Peptidase S10, serine carboxypeptidase	1031.48	168.18	-2.616642455	Down	0.907848372
POX01942	hypothetical protein	NA	NA	IPR002293; Amino acid/polyamine transporter I	200.577	47.6067	-2.074918263	Down	0.8565937
POX01949	hypothetical protein	NA	NA	NA	168.63	22.3133	-2.917883265	Down	0.883324435
POX01987	hypothetical protein	NA	NA	NA	302.963	32.0433	-3.241047061	Down	0.907456843
POX02008	hypothetical protein	NA	NA	NA	487.1	130.727	-1.89766452	Down	0.866275138
POX02016	hypothetical protein	NA	NA	IPR003154; S1/P1 nuclease	113.723	21.05	-2.433636153	Down	0.850720769
POX02066	hypothetical protein	NA	NA	IPR017795; Aromatic prenyltransferase, DMATS type	69.9133	4.87	-3.843573944	Down	0.851895355
POX02067	hypothetical protein	NA	NA	NA	560.39	72.93	-2.941846912	Down	0.91197722
POX02068	hypothetical protein	NA	NA	IPR001128; Cytochrome P450	149.163	11.3933	-3.710631141	Down	0.893183841
POX02231	hypothetical protein	NA	NA	IPR013920; Protein of unknown function DUF1774, fungi	1978.91	840.8	-1.234873856	Down	0.801690692
POX02355	hypothetical protein	NA	NA	IPR003439; ABC transporter-like	241.693	69.5967	-1.796087565	Down	0.845132586
POX02396	hypothetical protein	NA	NA	NA	174.027	49.3067	-1.819453763	Down	0.836323189
POX02405	hypothetical protein	NA	NA	IPR000109; Proton-dependent oligopeptide transporter family	1649.7	481.037	-1.777987836	Down	0.866631073
POX02407	hypothetical protein	NA	NA	IPR002198; Short-chain dehydrogenase/reductase SDR	569.82	146.187	-1.962694527	Down	0.872183663
POX02485	hypothetical protein	NA	NA	IPR001660; Sterile alpha motif domain	894.1	348.213	-1.360464752	Down	0.817262858
POX02502	hypothetical protein	NA	NA	NA	54.06	6.36	-3.087462841	Down	0.819202705
POX02529	hypothetical protein	NA	NA	NA	329.16	56.2867	-2.54792391	Down	0.89186688

POX02553	hypothetical protein	NA	NA	IPR004813; Oligopeptide transporter, OPT superfamily	815.58	141.487	-2.527160294	Down	0.904039865
POX02595	hypothetical protein	NA	NA	NA	108.883	23.77	-2.195569331	Down	0.838707955
POX02623	hypothetical protein	NA	NA	IPR000132; Nitrilase/cyanide hydratase, conserved site	65.0333	12.0133	-2.436542862	Down	0.814646734
POX02721	hypothetical protein	NA	NA	IPR000791; GPR1/FUN34/yaaH	1512.54	643.33	-1.233342424	Down	0.800800854
POX02754	hypothetical protein	NA	NA	NA	174.657	55.27	-1.659953194	Down	0.823634099
POX02811	hypothetical protein	NA	NA	IPR001107; Band 7 domain	727.753	287.437	-1.340205432	Down	0.812973839
POX02834	hypothetical protein	NA	NA	NA	115.383	37.5033	-1.621344113	Down	0.800088984
POX02847	hypothetical protein	NA	NA	NA	1687.87	309.33	-2.447987977	Down	0.904662751
POX02858	hypothetical protein	NA	NA	NA	160.943	27.7633	-2.535300121	Down	0.870937889
POX02972	hypothetical protein	NA	NA	NA	308.263	33.24	-3.21317101	Down	0.907296672
POX03078	hypothetical protein	NA	NA	IPR000718; Peptidase M13	306.86	113.08	-1.440236811	Down	0.812991636
POX03084	hypothetical protein	NA	NA	NA	56.3533	6.31333	-3.158027119	Down	0.824061221
POX03094	hypothetical protein	NA	NA	NA	81.32	7.82	-3.378369703	Down	0.856113187
POX03234	hypothetical protein	NA	NA	IPR008217; Ccc1 family	88.4833	19.8633	-2.155297989	Down	0.824434953
POX03465	hypothetical protein	NA	NA	NA	196.047	64.6267	-1.600995625	Down	0.820092543
POX03540	hypothetical protein	NA	NA	IPR011250; Outer membrane protein/outer membrane enzyme PagP, beta-barrel	121.99	9.97	-3.613025574	Down	0.883413419
POX03600	hypothetical protein	NA	NA	IPR011701; Major facilitator superfamily	64.1667	13.88	-2.208816472	Down	0.803861897
POX03627	hypothetical protein	NA	NA	IPR001663; Aromatic-ring-hydroxylating dioxygenase, alpha subunit	226.023	61.51	-1.877578835	Down	0.848691938
POX03666	hypothetical protein	NA	NA	IPR001128; Cytochrome P450	118.94	29.2567	-2.023396669	Down	0.832479089
POX03728	hypothetical protein	NA	NA	IPR011701; Major facilitator superfamily	88.5867	23.29	-1.927378933	Down	0.810304325
POX03742	hypothetical protein	NA	NA	IPR003663; Sugar/inositol transporter//<br>IPR020846; Major facilitator superfamily domain	417.503	140.7	-1.569165387	Down	0.835326571
POX03782	hypothetical protein	NA	NA	IPR006771; Blastomyces yeast-phase-specific protein	234.223	56.4033	-2.054032473	Down	0.859903897

POX03873	hypothetical protein	NA	NA	IPR001138; Zn(2)-C6 fungal-type DNA-binding domain	161.82	44.4467	-1.864242796	Down	0.836803702
POX03935	hypothetical protein	NA	NA	NA	61.5367	0.01	-12.58723058	Down	0.905872931
POX04276	hypothetical protein	NA	NA	IPR003663; Sugar/inositol transporter// IPR020846; Major facilitator superfamily domain	386.213	118.89	-1.699770599	Down	0.846769888
POX04278	hypothetical protein	NA	NA	NA	629.47	195.72	-1.685346434	Down	0.853176722
POX04316	hypothetical protein	NA	NA	IPR003663; Sugar/inositol transporter// IPR020846; Major facilitator superfamily domain	325.093	88.0267	-1.884841428	Down	0.857714896
POX04529	hypothetical protein	NA	NA	IPR003663; Sugar/inositol transporter// IPR020846; Major facilitator superfamily domain	1601.63	117.193	-3.772581497	Down	0.933279943
POX04531	hypothetical protein	NA	NA	IPR003663; Sugar/inositol transporter// IPR020846; Major facilitator superfamily domain	705.667	243.7	-1.533880612	Down	0.83835202
POX04534	hypothetical protein	NA	NA	NA	51.6667	2.85667	-4.176829201	Down	0.831802812
POX04552	hypothetical protein	NA	NA	NA	153.047	46.4967	-1.718772425	Down	0.823634099
POX04801	hypothetical protein	NA	NA	IPR015879; Aromatic-ring-hydroxylating dioxygenase, alpha subunit, C-terminal domain	224.083	67.6	-1.728940198	Down	0.837159637
POX04866	hypothetical protein	NA	NA	IPR000092; Polyprenyl synthetase	90.5467	8.87	-3.351655522	Down	0.862413241
POX04867	hypothetical protein	NA	NA	IPR001128; Cytochrome P450	165.88	19.5567	-3.084407556	Down	0.88686599
POX04888	hypothetical protein	NA	NA	NA	164.023	56.8933	-1.527569547	Down	0.807759388
POX05007	hypothetical protein	NA	NA	IPR001461; Aspartic peptidase	1814.46	11.3267	-7.323675741	Down	0.976864211
POX05017	hypothetical protein	NA	NA	IPR006461; PLAC8 motif-containing protein	333.41	90.8567	-1.875633094	Down	0.858124221
POX05076	hypothetical protein	NA	NA	IPR003663; Sugar/inositol transporter// IPR020846; Major facilitator superfamily domain	76.5833	11.2733	-2.764116297	Down	0.837675743
POX05112	hypothetical protein	NA	NA	IPR022234; Protein of unknown function DUF3759	366.887	98.3267	-1.899679835	Down	0.861274248
POX05220	hypothetical protein	NA	NA	NA	82.9633	8.47333	-3.291472331	Down	0.855792846
POX05271	hypothetical protein	NA	NA	IPR003864; Calcium-dependent channel, 7TM region, putative	120.837	39.2633	-1.621803729	Down	0.802082221

				phosphate					
POX05515	hypothetical protein	NA	NA	IPR003663; Sugar/inositol transporter// IPR020846; Major facilitator superfamily domain	239.003	53.7733	-2.152067929	Down	0.866933618
POX05575	hypothetical protein	NA	NA	IPR000250; Peptidase G1	5533.42	112.317	-5.622527502	Down	0.956006407
POX05576	hypothetical protein	NA	NA	IPR023753; FAD/NAD(P)-binding domain	121.547	7.58667	-4.001900367	Down	0.888200747
POX05646	hypothetical protein	NA	NA	NA	59.5333	4.14667	-3.84367369	Down	0.839028297
POX05703	hypothetical protein	NA	NA	IPR018306; Bacteriophage T5, Orf172 DNA-binding	111.863	12.7	-3.138836824	Down	0.87104467
POX05767	hypothetical protein	NA	NA	NA	5887.09	2047.42	-1.523749133	Down	0.844171561
POX05897	hypothetical protein	NA	NA	IPR003663; Sugar/inositol transporter// IPR020846; Major facilitator superfamily domain	390.337	97.9067	-1.995239986	Down	0.868677701
POX06008	hypothetical protein	NA	NA	IPR003663; Sugar/inositol transporter// IPR020846; Major facilitator superfamily domain	1347.05	475.037	-1.503689052	Down	0.838441004
POX06145	hypothetical protein	NA	NA	NA	197.013	4.69	-5.392561538	Down	0.920982381
POX06146	hypothetical protein	NA	NA	NA	78.6533	1.79333	-5.454792331	Down	0.878038797
POX06165	hypothetical protein	NA	NA	NA	121.543	22.9233	-2.406582014	Down	0.853657234
POX06231	hypothetical protein	NA	NA	NA	493.73	139.967	-1.818639021	Down	0.860847126
POX06317	hypothetical protein	NA	NA	IPR010440; Lipopolysaccharide kinase	141.483	20.7133	-2.772000468	Down	0.872859939
POX06345	hypothetical protein	NA	NA	IPR021706; Protein of unknown function DUF2990	1443.2	227.15	-2.667557365	Down	0.911834846
POX06525	hypothetical protein	NA	NA	NA	133.167	41.8733	-1.669129329	Down	0.812902652
POX06526	hypothetical protein	NA	NA	IPR003445; Cation transporter	155.57	43.34	-1.84379282	Down	0.833796049
POX06527	hypothetical protein	NA	NA	NA	76.04	17.33	-2.133486877	Down	0.812208578
POX06528	hypothetical protein	NA	NA	NA	1382.3	159.547	-3.115023864	Down	0.92329596
POX06530	hypothetical protein	NA	NA	IPR001568; Ribonuclease T2-like	1628.44	447.353	-1.864006846	Down	0.872450614
POX06549	hypothetical protein	NA	NA	IPR019931; LPXTG cell wall anchor domain	146.847	29.7533	-2.303187307	Down	0.858818295
POX06595	hypothetical protein	NA	NA	IPR001338; Hydrophobin	177.783	23.8167	-2.900076666	Down	0.88499733

POX06641	hypothetical protein	NA	NA	IPR011701; Major facilitator superfamily	73.0267	15.68	-2.21949782	Down	0.814184019
POX06730	hypothetical protein	NA	NA	IPR032675; Leucine-rich repeat domain, L domain-like	71.36	13.5433	-2.397532751	Down	0.820288308
POX06738	hypothetical protein	NA	NA	IPR006823; Neutral/alkaline nonlysosomal ceramidase	340.037	54.4067	-2.643834976	Down	0.894821143
POX06740	hypothetical protein	NA	NA	NA	90.05	19.68	-2.193996055	Down	0.827246841
POX06820	hypothetical protein	NA	NA	NA	7952.22	1535.89	-2.372282776	Down	0.903897491
POX06986	hypothetical protein	NA	NA	IPR006671; Cyclin, N-terminal	65.53	12.0867	-2.438739107	Down	0.815038263
POX07135	hypothetical protein	NA	NA	NA	691.6	279.123	-1.309035137	Down	0.808275494
POX07269	hypothetical protein	NA	NA	IPR005801; ADC synthase	60.2667	3.04333	-4.307636007	Down	0.846129204
POX07270	hypothetical protein	NA	NA	IPR025714; Methyltransferase domain	179.407	13.9067	-3.689384939	Down	0.899608471
POX07371	hypothetical protein	NA	NA	IPR000209; Peptidase S8/S53 domain	133.8	37.39	-1.83935374	Down	0.826962093
POX07428	hypothetical protein	NA	NA	IPR004648; Tetrapeptide transporter, OPT1/isp4	414.11	121.93	-1.763960907	Down	0.853568251
POX07457	hypothetical protein	NA	NA	NA	797.47	51.3033	-3.958305777	Down	0.931251112
POX07584	hypothetical protein	NA	NA	IPR006045; Cupin 1	125.407	35.78	-1.809388752	Down	0.821409503
POX07644	hypothetical protein	NA	NA	IPR001128; Cytochrome P450	280.283	63.4567	-2.143042314	Down	0.870457377
POX07646	hypothetical protein	NA	NA	NA	484.287	90.01	-2.427704086	Down	0.894305036
POX07666	hypothetical protein	NA	NA	IPR011701; Major facilitator superfamily// IPR020846; Major facilitator superfamily domain	89.34	24.55	-1.86358323	Down	0.80626446
POX07688	hypothetical protein	NA	NA	NA	373.27	125.3	-1.574833148	Down	0.833279943
POX07889	hypothetical protein	NA	NA	IPR002938; FAD-binding domain	213.533	62.7033	-1.767847253	Down	0.838796939
POX07903	hypothetical protein	NA	NA	IPR016191; Ribonuclease/ribotoxin	42.8367	3.33	-3.685252139	Down	0.806727176
POX08132	hypothetical protein	NA	NA	IPR016509; Membrane fusion mating protein FIG1	144.697	44.0633	-1.715381145	Down	0.821195942
POX08136	hypothetical protein	NA	NA	IPR001466; Beta-lactamase-related	181.01	32.6433	-2.471209113	Down	0.872272646
POX08190	hypothetical protein	NA	NA	IPR008928; Six-hairpin glycosidase-like	239.317	27.4167	-3.12579579	Down	0.899768642
POX08194	hypothetical protein	NA	NA	IPR009291; Vacuolar protein sorting-associated protein 62	512.41	88.0933	-2.540193882	Down	0.899644065
POX08305	hypothetical protein	NA	NA	IPR004813; Oligopeptide transporter, OPT superfamily	63.3967	9.58667	-2.725305812	Down	0.822495106

POX08371	hypothetical protein	NA	NA	IPR000209; Peptidase S8/S53 domain	186.45	16.53	-3.495630167	Down	0.898611853
POX08486	hypothetical protein	NA	NA	NA	820.68	336.397	-1.286656385	Down	0.80697633
POX08497	hypothetical protein	NA	NA	NA	207.187	57.34	-1.853317353	Down	0.843922406
POX08698	hypothetical protein	NA	NA	IPR001077; O-methyltransferase, family 2	49.3867	7.23333	-2.771389056	Down	0.801583912
POX08783	hypothetical protein	NA	NA	IPR003663; Sugar/inositol transporter// IPR020846; Major facilitator superfamily domain	219.06	14.9867	-3.869574635	Down	0.907990746
POX08891	hypothetical protein	NA	NA	IPR003663; Sugar/inositol transporter// IPR020846; Major facilitator superfamily domain	86.4467	16.5467	-2.385269721	Down	0.83340452
POX09258	hypothetical protein	GH127	NA	IPR008928; Six-hairpin glycosidase-like	147.01	32.3767	-2.182887928	Down	0.853016551
POX09299	hypothetical protein	NA	NA	IPR005592; Mono-/di-acylglycerol lipase, N-terminal	425.403	12.1967	-5.124272518	Down	0.936821498
POX09301	hypothetical protein	NA	NA	NA	548.073	113.997	-2.265377302	Down	0.890069407
POX09353	hypothetical protein	CE10	NA	IPR002018; Carboxylesterase, type B	427.117	46.07	-3.212730692	Down	0.914059441
POX09358	hypothetical protein	NA	NA	IPR004045; Glutathione S-transferase, N-terminal	432.217	92.3967	-2.225841994	Down	0.884570208
POX09365	hypothetical protein	NA	NA	IPR011701; Major facilitator superfamily	264.783	84.0367	-1.655721472	Down	0.835468945
POX09814	hypothetical protein	NA	NA	NA	93.1033	5.50667	-4.079581635	Down	0.873803168
POX09819	hypothetical protein	NA	NA	IPR027417; P-loop containing nucleoside triphosphate hydrolase	225.017	29.9433	-2.909725126	Down	0.892489767
POX09821	hypothetical protein	NA	NA	IPR002921; Fungal lipase-like domain	869.377	22.5167	-5.270916201	Down	0.945844456
POX09822	hypothetical protein	NA	NA	IPR007312; Phosphoesterase	2027.65	486.697	-2.058711474	Down	0.885566827
POX09829	hypothetical protein	NA	NA	NA	159.813	12.3333	-3.695753103	Down	0.89555081
POX09561	hypothetical protein	NA	NA	NA	98.1767	14.86	-2.723946069	Down	0.8527496
POX09563	hypothetical protein	NA	NA	NA	124.703	34.5267	-1.852717066	Down	0.824328172
POX09580	hypothetical protein	NA	NA	NA	70.5267	3.53	-4.320428766	Down	0.858675921
POX09666	hypothetical protein	NA	NA	IPR002119; Histone H2A	2497.5	906.09	-1.46275649	Down	0.834151984
POX09743	hypothetical protein	NA	NA	NA	26.9467	0.01333	-10.98085361	Down	0.817120484
POX09766	hypothetical protein	NA	NA	IPR016491; Septin	554.98	222.56	-1.318241457	Down	0.807154298

POX09786	hypothetical protein	NA	NA	NA	149.737	11.09	-3.755096273	Down	0.893699947
POX09798	hypothetical protein	NA	NA	IPR021264; Protein of unknown function DUF2841	108.22	10.0667	-3.426309192	Down	0.874248087
POX07932	pectin methylesterase	CE8	pectin methylesterase	IPR000070; Pectinesterase, catalytic	352.8	119.953	-1.556377312	Down	0.83142908
POX08067	pectin methylesterase	CE8	pectin methylesterase	IPR000070; Pectinesterase, catalytic	72.4533	17.76	-2.028420483	Down	0.802100018
POX02484	hypothetical protein	NA	NA	IPR001138; Zn(2)-C6 fungal-type DNA-binding domain	99.75	28.3433	-1.815307404	Down	0.810375512
POX07380	putative lysozyme	GH25	NA	IPR002053; Glycoside hydrolase, family 25	1480.75	249.747	-2.567790758	Down	0.908684819
POX06790	rhamnogalacturonan alpha-L-rhamnopyranohydrolase	GH28	polygalacturonase; rhamnogalacturonase	IPR000743; Glycoside hydrolase, family 28	103.993	22.57	-2.204012721	Down	0.836056238
POX06380	SUN domain-containing protein	GH132	NA	IPR005556; SUN family	136.87	39.3567	-1.798126321	Down	0.82516462

CAZy, Carbohydrate-Active Enzymes; GH, Glycoside hydrolase; CE, Carbohydrate esterase; GT, Glycosyl transferase; AA, Auxiliary activities; CWDE, Plant cell wall degrading enzyme; IPR, InterPro database (http://www.ebi.ac.uk/interpro/scan.html).
