# Supplementary material for: Comparative genomic, transcriptomic and secretomic profiling of Penicillium oxalicum HP7-1 and its cellulase and xylanase hyper-producing mutant EU2106, and identification of two novel regulatory genes of cellulase and xylanase gene expression
Source: Biotechnol Biofuels. 2016 Sep 23;9:203. doi: 10.1186/s13068-016-0616-9 (PMC5035457; doi:10.1186/s13068-016-0616-9)
Supplement: Supplementary file 6 — 10.1186/s13068-016-0616-9 List of 240 proteins secreted by P. oxalicum strains HP7-1 and EU2106 when grown in medium containing wheat bran and Avicel as the carbon source. [file 13068_2016_616_MOESM6_ESM.pdf]

Additional file 6: Table S3. List of the 240 proteins secreted by *P. oxalicum* strains HP7-1 and EU2106 when grown in medium containing wheat bran and Avicel as the carbon source.

Protein ID	Functional annotation	Predicted secretion signals	Protein contents (HP7-1 /EU2106)	Regulation (EU2106/ HP7-1)	CWDE	CAZy family	KEGG_ID	KEGG_First class	KEGG_Second class	KEGG_Third class
POX08861	acetyl xylan esterase	Y	0.193201824	Up	acetyl xylan esterase	CBM1; CE2	NA	NA	NA	NA
POX03711	acetyl xylan esterase	Y	0.655513261	Up	acetyl xylan esterase	CE5	afm:AFUA_3G00420	Unclassified	Metabolism	Others
POX01218	acetyl xylan esterase	Y	0.138369066	Up	acetyl xylan esterase	CBM1; CE1	afm:AFUA_8G06570	Unclassified	Metabolism	Others
POX03230	alpha-amylase	NA	0.151833799	Up	NA	GH13	NA	NA	NA	NA
POX09352	alpha-amylase Amy13A	Y	0.206921407	Up	NA	CBM20; GH13	pcs:Pc16g00630	Metabolism//Organismal Systems	Carbohydrate Metabolism// Digestive System	Starch and sucrose metabolism//Carbohydrate digestion and absorption
POX09085	alpha-galactosidase	Y	0.328768606	Up	alpha-galactosidase	CBM1; GH27; GH36	NA	NA	NA	NA
POX05540	alpha-L-arabinofuranosidase	Y	0.215981799	Up	beta-xylosidase/alpha-L-arabinofuranosidase	GH62	ang:ANI_1_108034	Metabolism	Carbohydrate Metabolism	Amino sugar and nucleotide sugar metabolism
POX06599	alpha-L-arabinofuranosidase	Y	0.375481626	Up	beta-xylosidase/alpha-L-arabinofuranosidase	CBM1; GH62	sco:SCO5932	Metabolism	Carbohydrate Metabolism	Amino sugar and nucleotide sugar metabolism
POX01914	alpha-L-arabinofuranosidase	Y	0.32949444	Up	beta-xylosidase/alpha-L-arabinofuranosidase	CBM42; GH54	ang:ANI_1_372134	Metabolism	Carbohydrate Metabolism	Amino sugar and nucleotide sugar metabolism
POX06600	alpha-L-arabinofuranosidase	Y	0.878482284	Up	beta-xylosidase/alpha-L-arabinofuranosidase	CBM1; GH43	afm:AFUA_2G00930	Unclassified	Metabolism	Others

POX06301	alpha-mannosidase	Y	0.244732122	Up	NA	GH47	pcs:Pc16g14170	Metabolism// Metabolism// Genetic Information Processing	Glycan Biosynthesis and Metabolism// Glycan Biosynthesis and Metabolism// Folding, Sorting and Degradation	N-Glycan biosynthesis // Various types of N-glycan biosynthesis // Protein processing in endoplasmic reticulum
POX01716	beta-1, 3-glucanase	NA	0.645715858	Up	NA	GH17	NA	NA	NA	NA
POX02423	beta-1, 3-glucanase	NA	0.359901992	Up	NA	GH64	NA	NA	NA	NA
POX06042	beta-1, 3-glucanosyltransferase	Y	0.194303023	Up	NA	GH17	pcs:Pc13g08730	Metabolism	Carbohydrate Metabolism	Starch and sucrose metabolism
POX09120	beta-glucanase	Y	0.602607135	Up	NA	GH16	NA	NA	NA	NA
POX03641	beta-glucosidase	NA	0.275155631	Up	beta-glucosidase	GH3	pcs:Pc21g18230	Metabolism// Metabolism// Metabolism	Carbohydrate Metabolism// Metabolism of Other Amino Acids// Biosynthesis of Other Secondary Metabolites	Starch and sucrose metabolism // Cyanoamino acid metabolism // Phenylpropanoid biosynthesis
POX06079	beta-glucosidase	NA	0.033276231	Up	beta-glucosidase	GH1	afm:AFUA_1G14710	Metabolism// Metabolism// Metabolism	Carbohydrate Metabolism// Metabolism of Other Amino Acids// Biosynthesis of Other Secondary Metabolites	Starch and sucrose metabolism // Cyanoamino acid metabolism // Phenylpropanoid biosynthesis
POX01961	beta-mannosidase	NA	0.165886776	Up	beta-mannosidase	GH2	pcs:Pc21g13810	Metabolism//Cellular Processes	Glycan Biosynthesis and Metabolism// Transport and Catabolism	Other glycan degradation // Lysosome
POX07573	beta-N-acetylhexosaminidase	Y	0.41018228	Up	NA	GH3	afm:AFUA_4G13770	Unclassified	Metabolism	Others
POX07665	beta-xylosidase	NA	0.29614444	Up	beta-xylosidase/alpha-	GH117;	afm:AFUA_8G04710	Metabolism//	Carbohydrate Metabolism//	Starch and sucrose

					L-arabinofuranosidase	GH43		Metabolism	Carbohydrate Metabolism	metabolism // Amino sugar and nucleotide sugar metabolism
POX00007	beta-xylosidase	Y	0.692954033	Up	beta-xylosidase/alpha-L-arabinofuranosidase	GH3; AA5	NA	NA	NA	NA
POX04688	bifunctional alpha-glucuronidase/N-acetyl beta-glucosaminidase	Y	0.188333321	Up	alpha-glucuronidase	GH84	NA	NA	NA	NA
POX02739	carbohydrate acetylesterase	NA	0.130505243	Up	NA	CE16	NA	NA	NA	NA
POX02490	cellobiohydrolase	Y	0.106743285	Up	cellobiohydrolase	GH7	afm:AFUA_6G11610	Unclassified	Metabolism	Others
POX05587	cellobiohydrolase CBHI	Y	0.249154222	Up	cellobiohydrolase	CBM1; GH7	afm:AFUA_6G11610	Unclassified	Metabolism	Others
POX04786	cellobiohydrolase Cel6A	Y	0.229568081	Up	cellobiohydrolase	CBM1; GH6	afm:AFUA_3G01910	Unclassified	Metabolism	Others
POX08897	cellulose monooxygenase	Y	0.199367758	Up	cellulose monooxygenase	CBM1; AA9	afm:AFUA_3G03870	Metabolism	Carbohydrate Metabolism	Starch and sucrose metabolism
POX02308	cellulose monooxygenase Cel61A	NA	0.130058235	Up	cellulose monooxygenase	AA9	afm:AFUA_1G12560	Unclassified	Metabolism	Others
POX06051	cellodextrin transporter cdt-c	NA	0.21323944	Up	NA	NA	NA	NA	NA	NA
POX01329	chitin deacetylase	NA	0.194671836	Up	NA	CE4	NA	NA	NA	NA
POX06241	chitin glucanosyltransferase	Y	0.311305518	Up	NA	GH16	afm:AFUA_1G16190	Unclassified	Metabolism	Others
POX02668	chitin	Y	0.685664458	Up	NA	GH16	afm:AFUA_2G03120	Unclassified	Metabolism	Others

	glucanosyltransferase									
POX01470	chitinase	Y	0.108273306	Up	NA	GH18	NA	NA	NA	NA
POX00089	chitinase	Y	0.48517094	Up	NA	GH18	afm:AFUA_8G01410	Metabolism	Carbohydrate Metabolism	Amino sugar and nucleotide sugar metabolism
POX07424	chitinase	NA	0.511199241	Up	NA	GH18	NA	NA	NA	NA
POX03021	chitinase	NA	0.491553476	Up	NA	GH18	afv:AFLA_031380	Metabolism	Carbohydrate Metabolism	Amino sugar and nucleotide sugar metabolism
POX01004	dipeptidyl-peptidase 3	NA	0.669676127	Up	NA	NA	pcs:Pc22g16760	Metabolism	Enzyme Families	Peptidases
POX06959	endo-beta-1, 3-glucanase	Y	0.325828277	Up	NA	GH81	afm:AFUA_1G04260	Unclassified	Metabolism	Others
POX01166	endo-beta-1, 4-glucanase	Y	0.18702696	Up	endo-beta-1, 4-glucanase	CBM1; GH5	act:ACLA_085250	Metabolism	Carbohydrate Metabolism	Starch and sucrose metabolism
POX07535	endo-beta-1, 4-glucanase	Y	0.380123591	Up	endo-beta-1, 4-glucanase	GH12	afm:AFUA_7G06150	Unclassified	Metabolism	Others
POX04137	endo-beta-1, 4-glucanase	NA	0.217282765	Up	endo-beta-1, 4-glucanase	CBM1; GH5	pcs:Pc22g19230	Metabolism	Carbohydrate Metabolism	Starch and sucrose metabolism
POX02740	endo-beta-1, 4-glucanase	Y	0.149162769	Up	endo-beta-1, 4-glucanase	GH5	nfi:NFIA_053150	Metabolism	Carbohydrate Metabolism	Starch and sucrose metabolism
POX05571	endo-beta-1, 4-glucanase 1	Y	0.2361764	Up	endo-beta-1, 4-glucanase	CBM1; GH7	nfi:NFIA_047960	Metabolism	Carbohydrate Metabolism	Starch and sucrose metabolism
POX06147	endo-beta-1, 4-glucanase Cel5A	Y	0.261346967	Up	endo-beta-1, 4-glucanase	CBM1; GH5	NA	NA	NA	NA
POX01896	endo-beta-1, 4-glucanase Cel5C	Y	0.142513504	Up	endo-beta-1, 4-glucanase	CBM1; CBM46; GH5	pcs:Pc21g14410	Metabolism	Carbohydrate Metabolism	Starch and sucrose metabolism
POX06983	endo-beta-1, 4-	Y	0.172388229	Up	endo-beta-1, 4-	GH12	afm:AFUA_1G04730	Unclassified	Metabolism	Others

	glucanase				glucanase					
POX08990	endo-beta-1, 4-xylanase	Y	0.244287914	Up	endo-beta-1, 4-xylanase	GH10	pcs:Pc20g07020	Unclassified	Metabolism	Carbohydrate metabolism
POX05916	endo-beta-1, 4-xylanase	Y	0.07332994	Up	endo-beta-1, 4-xylanase	GH10	NA	NA	NA	NA
POX00063	endo-beta-1, 4-xylanase	Y	0.14577176	Up	endo-beta-1, 4-xylanase	CBM1; GH10	afm:AFUA_6G13610	Unclassified	Metabolism	Carbohydrate metabolism
POX08484	endo-beta-1, 4-xylanase	Y	0.565009701	Up	Endo-beta-1, 4-xylanase	CBM1; GH11	afm:AFUA_3G00470	Unclassified	Metabolism	Carbohydrate metabolism
POX06601	endo-beta-1, 4-xylanase	Y	0.528570911	Up	Endo-beta-1, 4-xylanase	CBM1; GH30	NA	NA	NA	NA
POX01480	endo-N-acetyl-beta-d-glucosaminidase	NA	0.383395574	Up	NA	NA	afm:AFUA_5G03850	Metabolism	Carbohydrate Metabolism	Amino sugar and nucleotide sugar metabolism
POX03730	endopolygalacturonase	Y	0.331527343	Up	polygalacturonase; rhamnogalacturonase	GH28	ani:AN8327.2	Metabolism// Metabolism	Carbohydrate Metabolism// Carbohydrate Metabolism	Pentose and glucuronate interconversions // Starch and sucrose metabolism
POX09801	exo-beta-1, 3-galactanase	NA	0.080694524	Up	NA	GH43	NA	NA	NA	NA
POX07201	exo-beta-1, 3-glucanase	Y	0.376557463	Up	NA	GH17	afm:AFUA_3G00270	Unclassified	Metabolism	Others
POX00105	exo-beta-1, 3-glucanase	Y	0.262091777	Up	NA	GH55	pcs:Pc22g01380	Metabolism	Carbohydrate Metabolism	Starch and sucrose metabolism
POX09137	feruloyl esterase	Y	0.12349984	Up	feruloyl esterase	CBM1; CE1	NA	NA	NA	NA
POX02412	glucoamylase	Y	0.65634002	Up	NA	CBM20; GH15	nfi:NFIA_001210	Metabolism	Carbohydrate Metabolism	Starch and sucrose metabolism
POX01356	glucoamylase Amy15A	Y	0.47573462	Up	NA	CBM20; GH15	afm:AFUA_2G00690	Metabolism	Carbohydrate Metabolism	Starch and sucrose metabolism
POX09655	hypothetical protein	NA	0.154867096	Up	NA	NA	pcs:Pc20g08320	Metabolism	Enzyme Families	Peptidases

POX00645	hypothetical protein	NA	0.014904063	Up	NA	NA	NA	NA	NA	NA
POX01338	hypothetical protein	NA	0.032753727	Up	NA	NA	afm:AFUA_5G09230	Metabolism	Carbohydrate Metabolism	Pentose phosphate pathway
POX07330	hypothetical protein	NA	0.124747649	Up	NA	NA	pcs:Pc20g15010	Cellular Processes	Transport and Catabolism	Peroxisome
POX07408	hypothetical protein	NA	0.12697581	Up	NA	NA	afm:AFUA_2G15430	Unclassified	Metabolism	Others
POX01834	hypothetical protein	NA	0.053238668	Up	NA	NA	pcs:Pc21g15240	Metabolism// Metabolism// Human Diseases	Metabolism of Terpenoids and Polyketides// Metabolism of Terpenoids and Polyketides// Infectious Diseases	Prenyltransferases // Terpenoid backbone biosynthesis//Influenza A
POX09012	hypothetical protein	NA	0.171994975	Up	NA	NA	ang:ANI_1_1206064	Metabolism// Metabolism// Metabolism// Metabolism// Metabolism	Carbohydrate Metabolism// Carbohydrate Metabolism// Carbohydrate Metabolism// Carbohydrate Metabolism// Amino Acid Metabolism	Glycolysis//Gluconeogenesis //Citrate cycle (TCA cycle)// Pyruvate metabolism// Butanoate metabolism// Valine, leucine and isoleucine biosynthesis
POX02340	hypothetical protein	NA	0.027619375	Up	NA	NA	pcs:Pc13g07790	Metabolism// Metabolism// Metabolism	Carbohydrate Metabolism// Amino Acid Metabolism// Amino Acid Metabolism	Butanoate metabolism // Alanine, aspartate and glutamate metabolism // Tyrosine metabolism
POX09399	hypothetical protein	NA	0.050060818	Up	NA	NA	ani:AN6563.2	Genetic Information Processing	Translation	Translation factors
POX00588	hypothetical protein	NA	0.075428005	Up	NA	NA	pcs:Pc22g02800	Genetic Information Processing// Genetic Information Processing// Environmental Information Processing//	Folding, Sorting and Degradation// Folding, Sorting and Degradation// Signaling Molecules and Interaction// Transport and	Chaperones and folding catalysts // Protein processing in endoplasmic reticulum // Glycan bindng proteins // Phagosome //

								Cellular Processes// Organismal Systems	Catabolism// Immune System	Antigen processing and presentation
POX05184	hypothetical protein	Y	0.105330594	Up	NA	NA	NA	NA	NA	NA
POX04189	hypothetical protein	NA	0.108168472	Up	NA	NA	act:ACLA_046680	Metabolism// Metabolism	Amino Acid Metabolism// Metabolism of Other Amino Acids	Cysteine and methionine metabolism // Selenocompound metabolism
POX00862	hypothetical protein	NA	0.123494678	Up	NA	NA	pcs:Pc12g09030	Metabolism// Metabolism	Amino Acid Metabolism// Xenobiotics Biodegradation and Metabolism	Tyrosine metabolism // Styrene degradation
POX05099	hypothetical protein	NA	0.185626264	Up	NA	NA	NA	NA	NA	NA
POX05553	hypothetical protein	NA	0.162283987	Up	NA	NA	act:ACLA_065100	Metabolism// Environmental Information Processing	Carbohydrate Metabolism// Signal Transduction	Pyruvate metabolism // Two-component system
POX00551	hypothetical protein	NA	0.107094346	Up	NA	NA	pcs:Pc22g04680	Cellular Processes// Human Diseases	Transport and Catabolism// Neurodegenerative Diseases	Peroxisome // Huntington's disease
POX04212	hypothetical protein	Y	0.381806907	Up	NA	NA	NA	NA	NA	NA
POX00861	hypothetical protein	NA	0.096277151	Up	NA	NA	afm:AFUA_2G04220	Metabolism// Metabolism	Amino Acid Metabolism// Xenobiotics Biodegradation and Metabolism	Tyrosine metabolism // Styrene degradation
POX05221	hypothetical protein	NA	0.192558557	Up	NA	NA	cim:CIMG_09126	Genetic Information Processing	Folding, Sorting and Degradation	Chaperones and folding catalysts
POX02628	hypothetical protein	Y	0.313512237	Up	NA	NA	afm:AFUA_2G03510	Metabolism	Enzyme Families	Peptidases
POX07816	hypothetical protein	NA	0.084479206	Up	NA	NA	pcs:Pc22g17810	Unclassified	Poorly Characterized	Function unknown
POX09026	hypothetical protein	NA	0.117043329	Up	NA	NA	pcs:Pc22g11870	Metabolism	Amino Acid Metabolism	Tryptophan metabolism
POX07127	hypothetical protein	NA	0.136176001	Up	NA	NA	nfi:NFIA_021660	Metabolism	Carbohydrate Metabolism	Pentose phosphate pathway
POX01238	hypothetical protein	NA	0.085296724	Up	NA	NA	pcs:Pc12g07100	Metabolism//	Carbohydrate Metabolism//	Glycolysis//

								Metabolism// Metabolism// Metabolism// Metabolism// Metabolism// Metabolism// Metabolism	Lipid Metabolism//Amino Acid Metabolism// Metabolism of Cofactors and Vitamins//Xenobiotics Biodegradation and Metabolism//Xenobiotics Biodegradation and Metabolism//Xenobiotics Biodegradation and Metabolism//Xenobiotics Biodegradation and Metabolism	Gluconeogenesis//Fatty acid metabolism//Tyrosine metabolism//Retinol metabolism//Chloroalkane and chloroalkene degradation//Naphthalene degradation//Metabolism of xenobiotics by cytochrome P450//Drug metabolism - cytochrome P450
POX08322	hypothetical protein	NA	0.138983995	Up	NA	NA	pcs:Pc20g07230	Metabolism	Energy Metabolism	Oxidative phosphorylation
POX00147	hypothetical protein	NA	0.146485996	Up	NA	NA	pcs:Pc22g02000	Metabolism// Metabolism// Metabolism	Carbohydrate Metabolism// Carbohydrate Metabolism// Energy Metabolism	Citrate cycle (TCA cycle) // Glyoxylate and dicarboxylate metabolism // Carbon fixation pathways in prokaryotes
POX04943	hypothetical protein	NA	0.144221553	Up	NA	NA	pcs:Pc21g10100	Metabolism// Metabolism// Metabolism	Metabolism of Other Amino Acids// Xenobiotics Biodegradation and Metabolism// Xenobiotics Biodegradation and Metabolism	Glutathione metabolism // Metabolism of xenobiotics by cytochrome P450 // Drug metabolism - cytochrome P450
POX04910	hypothetical protein	NA	0.238054172	Up	NA	AA2	pcs:Pc12g13740	Metabolism// Metabolism//	Energy Metabolism// Amino Acid Metabolism// Amino	Methane metabolism // Phenylalanine metabolism //

								Metabolism// Metabolism	Acid Metabolism// Biosynthesis of Other Secondary Metabolites	Tryptophan metabolism // Phenylpropanoid biosynthesis
POX08673	hypothetical protein	NA	0.1598279	Up	NA	NA	pcs:Pc20g01610	Metabolism// Metabolism// Metabolism// Metabolism	Carbohydrate Metabolism// Carbohydrate Metabolism// Carbohydrate Metabolism// Energy Metabolism	Citrate cycle (TCA cycle) // Pyruvate metabolism // Glyoxylate and dicarboxylate metabolism // Carbon fixation in photosynthetic organisms
POX08949	hypothetical protein	NA	0.148501885	Up	NA	NA	pcs:Pc22g11240	Genetic Information Processing// Genetic Information Processing// Genetic Information Processing// Genetic Information Processing// Genetic Information Processing// Genetic Information Processing// Environmental Information Processing// Cellular Processes// Organismal Systems// Human Diseases// Human Diseases// Human Diseases	Transcription// Transcription// Translation// Folding, Sorting and Degradation// Folding, Sorting and Degradation// Folding, Sorting and Degradation// Signal Transduction// Transport and Catabolism// Immune System// Infectious Diseases// Infectious Diseases// Infectious Diseases	Spliceosome // Spliceosome // Ribosome Biogenesis // Chaperones and folding catalysts // Protein processing in endoplasmic reticulum // Proteasome // MAPK signaling pathway // Endocytosis // Antigen processing and presentation // Measles // Influenza A // Toxoplasmosis
POX00770	hypothetical protein	NA	0.103087459	Up	NA	NA	pcs:Pc12g08900	Metabolism//	Amino Acid Metabolism//	Alanine, aspartate and

								Metabolism	Amino Acid Metabolism	glutamate metabolism // Arginine and proline metabolism
POX07663	hypothetical protein	NA	0.17139734	Up	NA	CE10;CE1	NA	NA	NA	NA
POX00727	hypothetical protein	NA	0.137005665	Up	feruloyl esterase	NA	pcs:Pc12g08300	Unclassified	Metabolism	Others
POX07023	hypothetical protein	NA	0.305729415	Up	NA	NA	mgr:MGG_03648	Metabolism// Metabolism// Metabolism// Metabolism// Metabolism	Carbohydrate Metabolism// Carbohydrate Metabolism// Lipid Metabolism// Xenobiotics Biodegradation and Metabolism// Xenobiotics Biodegradation and Metabolism	Fructose and mannose metabolism//Butanoate metabolism//Linoleic acid metabolism//Chloroalkane and chloroalkene degradation//Bisphenol degradation
POX02372	hypothetical protein	NA	0.138069678	Up	NA	NA	NA	NA	NA	NA
POX00913	hypothetical protein	NA	0.184088182	Up	NA	NA	NA	NA	NA	NA
POX09390	hypothetical protein	NA	0.140650683	Up	NA	NA	NA	NA	NA	NA
POX00530	hypothetical protein	NA	0.168161276	Up	NA	NA	NA	NA	NA	NA
POX03582	hypothetical protein	NA	0.205570458	Up	NA	NA	ang:ANI_1_642104	Genetic Information Processing// Genetic Information Processing// Human Diseases// Human Diseases	Folding, Sorting and Degradation// Folding, Sorting and Degradation// Metabolic Diseases// Infectious Diseases	Chaperones and folding catalysts // RNA degradation // Type I diabetes mellitus // Tuberculosis
POX04116	hypothetical protein	NA	0.280336862	Up	NA	NA	afv:AFLA_044820	Metabolism// Metabolism// Metabolism// Metabolism	Carbohydrate Metabolism// Carbohydrate Metabolism// Carbohydrate Metabolism// Carbohydrate Metabolism	Glycolysis / Gluconeogenesis // Pentose phosphate pathway // Starch and sucrose metabolism // Amino sugar and nucleotide sugar

										metabolism
POX03509	hypothetical protein	NA	0.17947271	Up	NA	NA	pcs:Pc21g08590	Metabolism	Carbohydrate Metabolism	Pyruvate metabolism
POX07066	hypothetical protein	NA	0.161014772	Up	NA	NA	afv:AFLA_030160	Metabolism	Metabolism of Cofactors and Vitamins	Porphyrin and chlorophyll metabolism
POX04040	hypothetical protein	NA	0.227890106	Up	NA	NA	NA	NA	NA	NA
POX08090	hypothetical protein	NA	0.271073805	Up	NA	NA	afm:AFUA_2G02100	Metabolism// Metabolism// Metabolism// Metabolism// Metabolism	Carbohydrate Metabolism// Carbohydrate Metabolism// Carbohydrate Metabolism// Amino Acid Metabolism// Amino Acid Metabolism	Glycolysis / Gluconeogenesis // Citrate cycle (TCA cycle) // Pyruvate metabolism // Glycine, serine and threonine metabolism // Valine, leucine and isoleucine degradation
POX00170	hypothetical protein	NA	0.159530211	Up	NA	NA	afm:AFUA_6G12740	Metabolism// Metabolism// Metabolism	Biosynthesis of Other Secondary Metabolites// Xenobiotics Biodegradation and Metabolism// Xenobiotics Biodegradation and Metabolism	Tropane, piperidine and pyridine alkaloid biosynthesis// Aminobenzoate degradation //Bisphenol degradation
POX08331	hypothetical protein	NA	0.226682755	Up	NA	NA	pcs:Pc20g06940	Metabolism	Enzyme Families	Peptidases
POX07194	hypothetical protein	NA	0.488296191	Up	NA	NA	afv:AFLA_134120	Metabolism	Nucleotide Metabolism	Pyrimidine metabolism
POX01443	hypothetical protein	NA	0.565465358	Up	NA	NA	pcs:Pc21g15880	Metabolism// Metabolism	Nucleotide Metabolism// Nucleotide Metabolism	Purine metabolism // Pyrimidine metabolism
POX05577	hypothetical protein	NA	0.27384223	Up	NA	NA	NA	NA	NA	NA
POX03078	hypothetical protein	NA	0.385668192	Up	NA	NA	pcs:Pc18g01570	Metabolism	Enzyme Families	Peptidases
POX05098	hypothetical protein	NA	0.432294618	Up	NA	NA	NA	NA	NA	NA
POX08126	hypothetical protein	NA	0.631443967	Up	NA	NA	nfi:NFIA_068150	Metabolism// Metabolism	Amino Acid Metabolism// Metabolism of Cofactors and	Glycine, serine and threonine metabolism // Vitamin B6

									Vitamins	metabolism
POX08200	hypothetical protein	NA	0.264794598	Up	NA	NA	cpw:CPC735_015480	Genetic Information Processing	Folding, Sorting and Degradation	Chaperones and folding catalysts
POX02457	hypothetical protein	NA	0.361080198	Up	NA	NA	pcs:Pc18g03470	Metabolism	Enzyme Families	Peptidases
POX03157	hypothetical protein	NA	0.443354566	Up	NA	NA	aor:AOR_1_1206154	Metabolism	Nucleotide Metabolism	Purine metabolism
POX03806	hypothetical protein	NA	0.329265044	Up	NA	NA	NA	NA	NA	NA
POX04781	hypothetical protein	Y	0.453780758	Up	NA	NA	NA	NA	NA	NA
POX02119	hypothetical protein	NA	0.443960265	Up	NA	NA	NA	NA	NA	NA
POX04093	hypothetical protein	NA	0.272080435	Up	NA	NA	pcs:Pc22g19990	Genetic Information Processing// Genetic Information Processing// Human Diseases	Folding, Sorting and Degradation// Folding, Sorting and Degradation// Infectious Diseases	Chaperones and folding catalysts // RNA degradation // Tuberculosis
POX08821	hypothetical protein	Y	0.312153428	Up	NA	NA	NA	NA	NA	NA
POX08978	hypothetical protein	NA	0.416033758	Up	NA	NA	afm:AFUA_1G07200	Metabolism	Carbohydrate Metabolism	Pyruvate metabolism
POX01339	hypothetical protein	NA	0.292720326	Up	NA	NA	ang:ANI_1_470064	Cellular Processes// Human Diseases// Human Diseases// Human Diseases	Transport and Catabolism// Neurodegenerative Diseases// Neurodegenerative Diseases// Neurodegenerative Diseases	Peroxisome // Amyotrophic lateral sclerosis (ALS) // Huntington's disease // Prion diseases
POX08626	hypothetical protein	NA	0.548265359	Up	NA	NA	pcs:Pc20g01390	Metabolism	Enzyme Families	Peptidases
POX00441	hypothetical protein	NA	0.27056641	Up	NA	NA	ani:AN5604.2	Metabolism// Metabolism// Metabolism// Metabolism// Metabolism//	Carbohydrate Metabolism// Carbohydrate Metabolism// Carbohydrate Metabolism// Energy Metabolism// Energy Metabolism// Endocrine	Glycolysis / Gluconeogenesis // Pentose phosphate pathway // Fructose and mannose metabolism // Carbon fixation in photosynthetic

								Organismal Systems	System	organisms // Methane metabolism // Insulin signaling pathway
POX02610	hypothetical protein	Y	0.351682401	Up	NA	NA	NA	NA	NA	NA
POX03835	hypothetical protein	NA	0.318077575	Up	NA	NA	pcs:Pc13g05440	Metabolism	Enzyme Families	Peptidases
POX03389	hypothetical protein	Y	0.526631478	Up	NA	NA	NA	NA	NA	NA
POX00044	hypothetical protein	Y	0.476120777	Up	NA	NA	NA	NA	NA	NA
POX05561	hypothetical protein	NA	0.385064064	Up	NA	NA	pcs:Pc21g20440	Metabolism// Metabolism	Carbohydrate Metabolism// Energy Metabolism	Pentose phosphate pathway // Carbon fixation in photosynthetic organisms
POX05122	hypothetical protein	Y	0.44989899	Up	NA	NA	act:ACLA_015280	Genetic Information Processing// Genetic Information Processing	Folding, Sorting and Degradation// Folding, Sorting and Degradation	Chaperones and folding catalysts // Protein processing in endoplasmic reticulum
POX06888	hypothetical protein	Y	0.563538272	Up	NA	CE10	NA	NA	NA	NA
POX05260	hypothetical protein	Y	0.325096634	Up	NA	AA7	NA	NA	NA	NA
POX08835	hypothetical protein	NA	0.747389517	Up	NA	NA	NA	NA	NA	NA
POX03409	hypothetical protein	NA	0.564877255	Up	NA	NA	NA	NA	NA	NA
POX04694	hypothetical protein	NA	0.295425097	Up	NA	NA	ang:ANI_1_146074	Metabolism// Metabolism// Metabolism// Metabolism// Cellular Processes	Energy Metabolism// Amino Acid Metabolism// Amino Acid Metabolism// Amino Acid Metabolism// Transport and Catabolism	Methane metabolism // Alanine, aspartate and glutamate metabolism // Glycine, serine and threonine metabolism // Amino acid related enzymes // Peroxisome
POX08836	hypothetical protein	Y	0.638705231	Up	NA	NA	afm:AFUA_4G03230	Unclassified	Genetic Information	Translation proteins

									Processing	
POX06951	hypothetical protein	Y	0.770925678	Up	NA	NA	NA	NA	NA	NA
POX03949	hypothetical protein	NA	0.558493053	Up	NA	NA	NA	NA	NA	NA
POX05014	hypothetical protein	NA	0.620293206	Up	NA	NA	NA	NA	NA	NA
POX01875	hypothetical protein	Y	0.340310802	Up	NA	NA	afm:AFUA_5G02040	Metabolism	Lipid Metabolism	Glycerolipid metabolism
POX09822	hypothetical protein	Y	0.661997756	Up	NA	NA	NA	NA	NA	NA
POX01931	hypothetical protein	NA	0.512631147	Up	NA	NA	NA	NA	NA	NA
POX08190	hypothetical protein	Y	0.501979923	Up	NA	NA	NA	NA	NA	NA
POX05436	hypothetical protein	NA	0.447658508	Up	NA	NA	act:ACLA_075450	Metabolism	Energy Metabolism	Nitrogen metabolism
POX09284	hypothetical protein	Y	0.555323582	Up	NA	NA	NA	NA	NA	NA
POX02402	hypothetical protein	NA	0.849157308	Up	NA	NA	pcs:Pc18g02900	Metabolism	Lipid Metabolism	Glycerophospholipid metabolism
POX07371	hypothetical protein	Y	0.590840294	Up	NA	NA	NA	NA	NA	NA
POX01059	hypothetical protein	NA	0.363556886	Up	NA	NA	NA	NA	NA	NA
POX00590	hypothetical protein	NA	0.492116163	Up	NA	NA	NA	NA	NA	NA
POX02276	hypothetical protein	Y	0.583695424	Up	NA	NA	NA	NA	NA	NA
POX08037	hypothetical protein	NA	0.139367351	Up	NA	NA	NA	NA	NA	NA
POX03543	hypothetical protein	NA	0.808581871	Up	NA	NA	afm:AFUA_3G00310	Metabolism	Carbohydrate Metabolism	Inositol phosphate metabolism
POX03535	hypothetical protein	NA	0.23937428	Up	NA	CE12	NA	NA	NA	NA
POX08814	hypothetical protein	Y	0.667215711	Up	NA	NA	pcs:Pc12g14680	Metabolism// Genetic Information Processing// Cellular Processes	Enzyme Families// Folding, Sorting and Degradation// Transport and Catabolism	Peptidases // Chaperones and folding catalysts // Lysosome
POX08499	hypothetical protein	Y	0.171602643	Up	NA	NA	NA	NA	NA	NA
POX04230	hypothetical protein	Y	0.334213204	Up	NA	NA	NA	NA	NA	NA

POX06153	hypothetical protein	Y	0.174034808	Up	NA	NA	ure:UREG_00545	Metabolism// Metabolism// Metabolism// Environmental Information Processing	Carbohydrate Metabolism// Lipid Metabolism// Lipid Metabolism// Signaling Molecules and Interaction	Inositol phosphate metabolism // Glycerophospholipid metabolism // Ether lipid metabolism // Bacterial toxins
POX01867	hypothetical protein	Y	0.089929769	Up	NA	NA	NA	NA	NA	NA
POX00144	hypothetical protein	Y	0.271840884	Up	NA	AA7	NA	NA	NA	NA
POX08906	hypothetical protein	Y	0.413094757	Up	NA	NA	NA	NA	NA	NA
POX09419	hypothetical protein	Y	0.43562236	Up	NA	NA	NA	NA	NA	NA
POX07164	hypothetical protein	NA	0.680041193	Up	NA	NA	afv:AFLA_133950	Metabolism// Metabolism// Metabolism// Metabolism	Carbohydrate Metabolism// Carbohydrate Metabolism// Carbohydrate Metabolism// Energy Metabolism	Glycolysis / Gluconeogenesis // Fructose and mannose metabolism // Inositol phosphate metabolism // Carbon fixation in photosynthetic organisms
POX05578	hypothetical protein	Y	0.29293567	Up	NA	NA	afm:AFUA_4G13750	Metabolism// Metabolism	Amino Acid Metabolism// Metabolism of Cofactors and Vitamins	Lysine degradation // Biotin metabolism
POX08136	hypothetical protein	NA	0.174376526	Up	NA	NA	NA	NA	NA	NA
POX03395	hypothetical protein	NA	0.192353311	Up	NA	NA	NA	NA	NA	NA
POX00796	hypothetical protein	NA	0.631192578	Up	NA	NA	afm:AFUA_2G05000	Metabolism// Metabolism	Amino Acid Metabolism// Enzyme Families	Arginine and proline metabolism // Peptidases
POX04370	hypothetical protein	Y	0.40623745	Up	NA	NA	NA	NA	NA	NA
POX07409	hypothetical protein	Y	0.371877772	Up	NA	GH131	NA	NA	NA	NA
POX08885	hypothetical protein	Y	0.493338206	Up	NA	NA	NA	NA	NA	NA

POX07081	hypothetical protein	Y	0.095928255	Up	NA	NA	NA	NA	NA	NA
POX00050	hypothetical protein	Y	0.286049852	Up	NA	CE10	NA	NA	NA	NA
POX00876	hypothetical protein	NA	0.626085656	Up	NA	NA	pcs:Pc12g09820	Metabolism// Environmental Information Processing	Carbohydrate Metabolism// Signal Transduction	Pyruvate metabolism // MAPK signaling pathway - yeast
POX02016	hypothetical protein	Y	0.44830853	Up	NA	NA	NA	NA	NA	NA
POX03232	hypothetical protein	Y	0.6145695	Up	NA	NA	NA	NA	NA	NA
POX03244	hypothetical protein	NA	0.042175931	Up	NA	NA	NA	NA	NA	NA
POX03937	hypothetical protein	NA	0.055651683	Up	NA	NA	afv:AFLA_026470	Metabolism// Metabolism// Metabolism// Metabolism// Metabolism// Metabolism// Metabolism// Organismal Systems	Amino Acid Metabolism// Amino Acid Metabolism// Amino Acid Metabolism// Amino Acid Metabolism// Amino Acid Metabolism// Amino Acid Metabolism// Amino Acid Metabolism// Digestive System	Alanine, aspartate and glutamate metabolism // Cysteine and methionine metabolism // Arginine and proline metabolism // Tyrosine metabolism // Phenylalanine metabolism // Phenylalanine, tyrosine and tryptophan biosynthesis // Amino acid related enzymes // Fat digestion and absorption
POX02962	hypothetical protein	NA	0.155206301	Up	NA	NA	pcs:Pc12g16040	Metabolism// Metabolism	Carbohydrate Metabolism// Energy Metabolism	Glycolysis / Gluconeogenesis // Methane metabolism
POX06646	hypothetical protein	NA	0.088847601	Up	NA	NA	act:ACLA_088550	Metabolism// Metabolism// Genetic Information Processing	Carbohydrate Metabolism// Energy Metabolism// Folding, Sorting and Degradation	Glycolysis / Gluconeogenesis // Methane metabolism // RNA degradation

POX05650	hypothetical protein	NA	0.225754044	Up	NA	NA	afm:AFUA_6G10880	Metabolism// Metabolism	Metabolism of Terpenoids and Polyketides// Xenobiotics Biodegradation and Metabolism	Geraniol degradation // Naphthalene degradation
POX02826	hypothetical protein	Y	0.37839669	Up	NA	NA	afm:AFUA_2G01250	Metabolism// Metabolism	Amino Acid Metabolism// Metabolism of Cofactors and Vitamins	Lysine degradation // Biotin metabolism
POX05715	hypothetical protein	Y	0.251494142	Up	NA	CE10	afm:AFUA_8G04210	Unclassified	Metabolism	Others
POX01547	hypothetical protein	NA	0.204086057	Up	NA	NA	mgr:MGG_08166	Metabolism	Carbohydrate Metabolism	Glyoxylate and dicarboxylate metabolism
POX02774	hypothetical protein	NA	0.418720504	Up	NA	NA	afm:AFUA_3G11920	Metabolism// Metabolism// Metabolism// Metabolism// Metabolism	Carbohydrate Metabolism// Metabolism of Terpenoids and Polyketides// Xenobiotics Biodegradation and Metabolism// Xenobiotics Biodegradation and Metabolism// Xenobiotics Biodegradation and Metabolism	Butanoate metabolism // Limonene and pinene degradation // Benzoate degradation // Bisphenol degradation // Naphthalene degradation
POX01488	hypothetical protein	NA	0.513460551	Up	NA	NA	pcs:Pc22g13950	Metabolism	Enzyme Families	Peptidases
POX01912	hypothetical protein	Y	0.434004943	Up	NA	NA	NA	NA	NA	NA
POX01526	hypothetical protein	NA	0.327841782	Up	NA	NA	pcs:Pc22g15910	Metabolism	Enzyme Families	Peptidases
POX03009	hypothetical protein	NA	0.30300342	Up	NA	NA	pcs:Pc18g01490	Metabolism	Carbohydrate Metabolism	Glycolysis / Gluconeogenesis
POX01696	hypothetical protein	NA	0.468909064	Up	NA	NA	NA	NA	NA	NA
POX02681	hypothetical protein	NA	0.214337227	Up	NA	NA	ure:UREG_01779	Genetic Information Processing// Genetic	Transcription//Transcription	Spliceosome//Spliceosome

								Information Processing		
POX07380	lysozyme	Y	0.13982375	Up	NA	GH25	afm:AFUA_6G10130	Unclassified	Metabolism	Others
POX01937	mannanase	Y	0.279758209	Up	beta-1, 4-mannanase	CBM1; GH5	NA	NA	NA	NA
POX04920	pectin lyase	Y	0.092264282	Up	pectate lyase; pectin lyase	PL1	afm:AFUA_2G00800	Unclassified	Metabolism	Others
POX07932	pectin methylesterase	Y	0.342956658	Up	pectin methylesterase	CE8	NA	NA	NA	NA
POX08761	PHB depolymerase family esterase	NA	0.0849636	Up	NA	CBM1; CE1	afm:AFUA_4G03560	Unclassified	Metabolism	Others
POX05580	polygalacturonase	Y	0.37641125	Up	polygalacturonase; rhamnogalacturonase	GH28	nfi:NFIA_102450	Metabolism// Metabolism	Carbohydrate Metabolism// Carbohydrate Metabolism	Pentose and glucuronate interconversions // Starch and sucrose metabolism
POX03893	probable Polyubiquitin protein	NA	0.168057549	Up	NA	NA	pcs:Pc22g12390	Genetic Information Processing// Organismal Systems	Folding, Sorting and Degradation// Endocrine System	Ubiquitin system // PPAR signaling pathway
POX07745	protein disulfide-isomerase	Y	0.304684917	Up	NA	NA	pcs:Pc21g11280	Genetic Information Processing// Genetic Information Processing	Folding, Sorting and Degradation// Folding, Sorting and Degradation	Chaperones and folding catalysts // Protein processing in endoplasmic reticulum
POX08813	starch binding domain- and chitin binding domain-containing protein	Y	0.169606456	Up	NA	CBM20; AA13	NA	NA	NA	NA
POX08485	swollenin	Y	0.16793311	Up	expansin-like protein	CBM1	afm:AFUA_6G03280	Unclassified	Metabolism	Others
POX09046	TPA: Calmodulin (CaM)	NA	0.313284837	Up	NA	NA	cpw:CPC735_040420	Environmental Information Processing//	Signal Transduction//Signal Transduction// Cell Growth	Calcium signaling pathway // Phosphatidylinositol

								Environmental Information Processing// Cellular Processes// Organismal Systems// Organismal Systems// Organismal Systems// Organismal Systems// Organismal Systems// Organismal Systems// Organismal Systems// Organismal Systems// Organismal Systems// Organismal Systems// Organismal Systems// Organismal Systems// Human Diseases// Human Diseases// Human Diseases// Human Diseases	and Death// Endocrine System// Endocrine System// Endocrine System// Circulatory System// Digestive System// Digestive System// Nervous System// Nervous System// Sensory System// Sensory System// Sensory System// Environmental Adaptation// Cancers// Neurodegenerative Diseases// Infectious Diseases// Infectious Diseases	signaling system // Oocyte meiosis // Insulin signaling pathway // GnRH signaling pathway // Melanogenesis // Vascular smooth muscle contraction // Salivary secretion // Gastric acid secretion // Long-term potentiation // Neurotrophin signaling pathway // Phototransduction // Phototransduction - fly // Olfactory transduction // Plant-pathogen interaction // Glioma // Alzheimer's disease // Pertussis // Tuberculosis
POX00348	TPA: Histone H 4.2	NA	0	Up	NA	NA	afv:AFLA_137440	Genetic Information Processing// Human Diseases	Replication and Repair// Immune System Diseases	Chromosome // Systemic lupus erythematosus
POX03228	alpha-1, 3-glucan synthase	NA	1.115365578	Down	NA	GH13; GT4; GT5	ang:ANI_1_360084	Metabolism	Glycan Biosynthesis and Metabolism	Glycosyltransferases
POX08017	alpha-1, 6-mannanase	Y	4.303423037	Down	NA	GH76	NA	NA	NA	NA

POX06067	alpha-mannosidase	Y	2.817795192	Down	NA	GH92	NA	NA	NA	NA
POX07083	beta-1, 3-1, 4-glucanase	Y	1.732813547	Down	NA	GH16	afm:AFUA_1G05290	Unclassified	Metabolism	Others
POX04390	beta-1, 3-glucanosyltransglycosylase	NA	1.196200572	Down	NA	CBM43; GH72	NA	NA	NA	NA
POX05604	beta-1, 3-glucanosyltransglycosylase	NA	1.321328741	Down	NA	GH72	NA	NA	NA	NA
POX04626	beta-1, 3-glucanosyltransglycosylase	Y	1.774401591	Down	NA	CBM43; GH72	NA	NA	NA	NA
POX03294	beta-1, 3-glucanosyltransglycosylase	NA	2.262135895	Down	NA	GH72	NA	NA	NA	NA
POX08390	beta-1, 6-glucanase	Y	3.508932166	Down	NA	GH30	act:ACLA_060250	Metabolism// Metabolism// Cellular Processes	Lipid Metabolism// Glycan Biosynthesis and Metabolism// Transport and Catabolism	Sphingolipid metabolism // Other glycan degradation // Lysosome
POX05968	cellulose monooxygenase	Y	1.717975307	Down	cellulose monooxygenase	AA9	afm:AFUA_1G12560	Unclassified	Metabolism	Others
POX09387	chitinase	Y	2.261724137	Down	NA	GH18	afm:AFUA_8G00700	Metabolism	Carbohydrate Metabolism	Amino sugar and nucleotide sugar metabolism
POX08903	chitosanase	Y	4.426723279	Down	NA	GH75	afm:AFUA_8G00930	Metabolism	Carbohydrate Metabolism	Amino sugar and nucleotide sugar metabolism
POX07820	ecm33 domain-containing protein	Y	2.132719504	Down	NA	NA	NA	NA	NA	NA

POX06689	endo-beta-1, 4-galactanase	Y	1.420300361	Down	endo-beta-1, 4-galactanase	GH53	nfi:NFIA_017780	Unclassified	Metabolism	Others
POX09440	hypothetical protein	NA	0.152110713	Down	NA	NA	zma:100501669	Metabolism// Metabolism	Carbohydrate Metabolism// Energy Metabolism	Glyoxylate and dicarboxylate metabolism // Methane metabolism
POX05595	hypothetical protein	NA	1.230071084	Down	NA	NA	pcs:Pc06g00090	Metabolism	Enzyme Families	Peptidases
POX05569	hypothetical protein	Y	1.043918718	Down	NA	NA	pcs:Pc21g03510	Metabolism	Enzyme Families	Peptidases
POX07147	hypothetical protein	NA	1.013517804	Down	NA	NA	NA	NA	NA	NA
POX05322	hypothetical protein	NA	1.224700911	Down	NA	NA	NA	NA	NA	NA
POX08831	hypothetical protein	Y	1.189973656	Down	NA	NA	NA	NA	NA	NA
POX01123	hypothetical protein	NA	1.435672029	Down	NA	NA	NA	NA	NA	NA
POX08818	hypothetical protein	Y	1.597842124	Down	NA	NA	NA	NA	NA	NA
POX09361	hypothetical protein	NA	1.526587565	Down	NA	NA	NA	NA	NA	NA
POX03840	hypothetical protein	NA	2.070010447	Down	NA	NA	pcs:Pc13g05400	Metabolism// Metabolism	Lipid Metabolism// Enzyme Families	Arachidonic acid metabolism // Peptidases
POX08878	hypothetical protein	NA	1.634428618	Down	NA	CE10	NA	NA	NA	NA
POX06904	hypothetical protein	Y	2.159574145	Down	NA	NA	NA	NA	NA	NA
POX09222	hypothetical protein	Y	2.97009438	Down	NA	NA	NA	NA	NA	NA
POX09299	hypothetical protein	Y	1.968835571	Down	NA	NA	NA	NA	NA	NA
POX08849	hypothetical protein	Y	2.010643482	Down	NA	NA	afm:AFUA_7G00800	Metabolism// Metabolism// Human Diseases	Metabolism of Cofactors and Vitamins// Xenobiotics Biodegradation and Metabolism// Infectious Diseases	Riboflavin metabolism // Aminobenzoate degradation // Tuberculosis
POX01929	hypothetical protein	Y	2.931151334	Down	NA	NA	afm:AFUA_5G01200	Metabolism	Enzyme Families	Peptidases
POX09821	hypothetical protein	NA	2.237949134	Down	NA	NA	NA	NA	NA	NA

POX09258	hypothetical protein	Y	3.162339876	Down	NA	GH127	mau:Micau_2566	Unclassified	Poorly Characterized	Function unknown
POX05007	hypothetical protein	Y	3.10830225	Down	NA	NA	pcs:Pc21g03510	Metabolism	Enzyme Families	Peptidases
POX02885	hypothetical protein	NA	2.593865666	Down	NA	NA	NA	NA	NA	NA
POX02880	hypothetical protein	NA	1.503935719	Down	NA	NA	NA	NA	NA	NA
POX06146	hypothetical protein	Y	3.178895749	Down	NA	NA	NA	NA	NA	NA
POX06530	hypothetical protein	Y	2.13949975	Down	NA	NA	pcs:Pc06g00430	Unclassified	Genetic Information Processing	Translation proteins
POX08770	hypothetical protein	NA	3.417927461	Down	NA	NA	NA	NA	NA	NA
POX01524	hypothetical protein	NA	6.996508084	Down	expansin-like protein	CBM63	NA	NA	NA	NA
POX01158	SUN domain-containing protein	Y	1.644344603	Down	NA	GH132	NA	NA	NA	NA

CAZy, Carbohydrate-Active Enzymes; GH, Glycoside hydrolase; CE, Carbohydrate esterase; GT, Glycosyl transferase; AA, Auxiliary activities; CBM, Carbohydrate-binding modules; CWDE, Plant cell wall degrading enzyme; KEGG, Kyoto Encyclopedia of Genes and Genomes (http://www.genome.jp/kegg/); NA, No annotation.
