# Supplementary material for: Comparative genomic, transcriptomic and secretomic profiling of Penicillium oxalicum HP7-1 and its cellulase and xylanase hyper-producing mutant EU2106, and identification of two novel regulatory genes of cellulase and xylanase gene expression
Source: Biotechnol Biofuels. 2016 Sep 23;9:203. doi: 10.1186/s13068-016-0616-9 (PMC5035457; doi:10.1186/s13068-016-0616-9)
Supplement: Supplementary file 7 — 10.1186/s13068-016-0616-9 Eleven candidate transcription factors identified from the initial screen. [file 13068_2016_616_MOESM7_ESM.pdf]

Additional file 7: Table S4. Eleven candidates with putative biological function in the first screening of transcription factors

Gene ID	BlastP function analysis	Candidate transcription factors of cellulase gene expression	Nucleoside mutation	Amino acid mutation	SNVs position	FPKM in HP7-1	FPKM in EU2106	Log_2(EU2106 /HP7-1)	Regulation in EU2106	Probability
POX00301	glutamine synthase	No	G->A	–	1500_up	286.52	1135.43	1.98653	Up	0.87933796
POX06751	putative alpha-glucosidase	No	G<->A	V<->I	CDS	185.063	27.3333	-2.7593	Down	0.882434597
POX06820	Glycosyl phosphatidyl inositol (GPI) anchored serine-rich protein	No	G->T	–	1500_up	7952.22	1535.89	-2.3723	Down	0.903897491
POX07291	putative mitochondrial phosphate transporter Pic2	Yes[a]	G->A	–	200_down	83.6433	759.52	3.18277	Up	0.920181527
POX09827	hypothetical protein (only 35 amino acids)	No	C->G	–	1500_up	39.5267	162.437	2.03898	Up	0.847962271
POX01960	Known transcription factor ClrB ortholog of cellulase gene expression	Yes	G<->A	S<->N	CDS	NA	NA	NA	NA	NA
POX03199	CCR4-Not complex component, Not1	No[b]	G<->A	R<->H	CDS	NA	NA	NA	NA	NA
POX08522	Forkhead transcription factor	Yes[c]	C<->T	H<->Y	CDS	NA	NA	NA	NA	NA
POX02484	GAL4-like Zn_2Cys_6 binuclear cluster DNA binding domain and Fungal_Trans_2	Yes[d]	–	–	–	99.75	28.3433	-1.8153	Down	0.810375512
POX03873	GAL4-like Zn2Cys6 binuclear cluster DNA binding domain	No[d]	–	–	–	161.82	44.4467	-1.8642	Down	0.836803702
POX07938	GAL4-like Zn2Cys6 binuclear cluster DNA binding domain	No[d]	–	–	–	13.6367	201.973	3.8886	Up	0.90560598

1500_up, 1500-bp upstream of start codon ATG; 200_down, 200-bp downstream of stop codon TAA, TGA or TAG; CDS, coding DNA sequence; CAZy, Carbohydrate-Active Enzymes; CWDE, Plant cell wall degrading enzyme; NA, no .

[a] Mitochondrial phosphate transporter Pic is a mitochondrial solute carrier protein, delivering phosphate, a key substrate of oxidative phosphorylation[1].

[b] Not1, CCR4-Not complex component, is essential for yeast viability as a member of CCR4-Not complex by regulating transcription expression of the interrelated genes[2].

[c] The forkhead box binding nucleasomal DNA, participates a wide variety of cellular processes including cell cycle regulation, cell death control, pre-mRNA processing and

morphogenesis in yeast and filamentous fungi, as well as in mammals[3].

[d] Generally, the important transcription factors contains two domains GAL4-like Zn2Cys6 binuclear cluster DNA binding domain and Fungal_Trans_2/Fungal_TF_MHR. The Fungal_Trans_2/Fungal_TF_MHR domain is required for the protein function[4].

Reference:

1. Seiferta, E. L., Ligetib, E., Mayrc, J. A., Sondheimerd, N., Hajnóczkya, G. The mitochondrial phosphate carrier: role in oxidative metabolism, calcium handling and mitochondrial disease. Biochem. Bioph. Res. Co. 464, 369-375 (2015).

2. Maillet, L. & Collart, M. A. Interaction between Not1p, a component of the Ccr4-Not complex, a global regulator of transcription, and Dhh1p, a putative RNA helicase. J. Biol. Chem. 277, 2835-2842 (2002).

3. Murakami, H., Aiba, H., Nakanishi, M. & Tonami, Y.M. Regulation of yeast forkhead transcription factors and FoxM1 by cyclin-dependent and polo-like kinases. Cell Cycle 9, 3253-3262 (2010).

4. MacPherson, S., Larochelle, M. & Turcotte, B. A fungal family of transcriptional regulators: the zinc cluster proteins. Microbiol. Mol. Biol. Rev. 70, 583-604 (2014).
