# Supplementary material for: Comparative genomic, transcriptomic and secretomic profiling of Penicillium oxalicum HP7-1 and its cellulase and xylanase hyper-producing mutant EU2106, and identification of two novel regulatory genes of cellulase and xylanase gene expression
Source: Biotechnol Biofuels. 2016 Sep 23;9:203. doi: 10.1186/s13068-016-0616-9 (PMC5035457; doi:10.1186/s13068-016-0616-9)
Supplement: Supplementary file 11 — 10.1186/s13068-016-0616-9 Primers used in this study. [file 13068_2016_616_MOESM11_ESM.pdf]

Additional file 11: Table S5. Primers used in this study

Primer name	Sequence (5'-3’)
**Primers used for the construction of deletion mutants**	
PoxClrB_Left_F	GGAATTCGATAGATCGCGGACCCGAGG
PoxClrB_Left_R	GTTTAGAGGTAATCCTTCTTTCTAGAAATGACTGTACGGTAGCACC
PoxClrB_nest_F	GAACAGACTGGTGGCGTAGAA
PoxClrB_nest_R	TGCGGCGACATAGCGTAGT
PoxClrB_Right_F	CTTCAATATCATCTTCTGTCGACGGGATCTTGAACTTCTGTTG
PoxClrB_Right_R	CCCAAGCTTGAACTGAACAACGCCATTG
POX02484_Left_F	ATCTGATTACTCCCTGGATGCGAAC
POX02484_Left_R	TTTAGAGGTAATCCTTCTTTCTAGACTTGATTATGAGAGCCAATGCTCG
POX02484_Nest_F	TATCTCTTACGAAATGATTGGTCTT
POX02484_Nest_R	CAAGGGTGGATTGTCCTCTAA
POX02484_Right_F	TCCTTCAATATCATCTTCTGTCGACATTTCTACAGTTTTGAGCGATCG
POX02484_Right_R	GATAGCGAAACAGTAAAGGCG
POX07291_Left_F	ATGGACGGGAGATGTTTCA
POX07291_Left_R	GGTAATCCTTCTTTCTAGACTTGACTTGTTAATCGTTCCAGG
POX07291_Nest_F	CCGAACGCCAAAGGAAAGA
POX07291_Nest_R	CTCAAGGCCAACTGACCCATCTGGAGTTGCGGTGTTGGAAT
POX07291_Right_F	AATATCATCTTCTGTCGACATAATAGTGCATCGTTCCAGG
POX07291_Right_R	CGCCGACACAGCCCCTCTA
POX08522_Left_F	GGGACGATGTATGAGGTTGGGA
POX08522_Left_R	GGTAATCCTTCTTTCTAGATATGTGCAAGGATCGAGAACG
POX08522_Nest_F	AAAAGTTGGAGATGCGGAAGTTGGT
POX08522_Nest_R	GACATAGGACCTTTGGTTGACTG

POX08522_Right_F	CAATATCATCTTCTGTCGACCGGGGGTTCCTTGTTCCTCCCTCCTCC
POX08522_Right_R	CAGGGTTATGAGAAGGTGAGTT
PoxClrB_confirm_F	AAGTCATCGTGTGGCTGGAT
PoxClrB_confirm_R	CGGTCGGATCGTTGAGGAA
PoxClrBleft_confirm-F	GTCACACTCCACGCAACTG
PoxClrBleft_confirm-R	TCGCCTTCTATCGCCTTCTT
PoxClrBright_confirm-F	GATCCGCCTGGACGACTAA
PoxClrBright_confirm-F	AATCACACCTGCTAGGAATGC
POX02484_confirm-F	CGAGATTGATGTGAAGACCGAG
POX02484_confirm-R	GATAAATCCGGGCAGCGAG
POX02484left_confirm-F	CGGCTGGTACTAGAGATCTGATCA
POX02484left_confirm-R	ACCTCGAAATCATTCCTACTAAG
POX02484right_confirm-F	ACCAGTTGCCTAAATGAACCATC
POX02484right_confirm-F	TCGGCATGTTGACAAGATGATGAGA
POX07291_confirm_F	GAACGCCGACCGAAAGTGT
POX07291_confirm_R	AGGACTACGGCGATGAATGG
POX07291left_confirm-F	ATGGACGGGAGATGTTTCA
POX07291left_confirm-R	GCCCTGGGTTCGCAAAGATA
POX07291right_confirm-F	AATAATGTCCTCGTTCCTGTCTGC
POX07291right_confirm-F	CGCCGACACAGCCCCTCTA
POX08522_confirm_F	CCATTGCTCCCTTCATCCA
POX08522_confirm_R	GCTTCATCTGCGGTATCTTCC
POX08522left_confirm-F	GGGACGATGTATGAGGTTGGGA
POX08522left_confirm-R	GTGAATGCTCCGTAACACCCAAT
POX08522right_confirm-F	CGCTACTGCTTACAAGTGGGCTGAT

POX08522right_confirm-F	CAGGGTTATGAGAAGGTGAGTT
G418_F	TCTAGAAAGAAGGATTACC
G418_R	GTCGACAGAAGATGATATT
**Primers used for qRT-PCR**	
Actin-F	CTCCATCCAGGCCGTTCTG
Actin-R	CATGAGGTAGTCGGTCAAGTCAC
POX05587-F	GTACTTGCGATCCTGATGGG
POX05587-R	CCACGGTGAAGGGAGACTTG
POX04786-F	TACTACGCTTCCGAGGTTCAGAG
POX04786-R	GTGTCCAGCCAAACGAAGG
POX02490-F	TGAAACCCACCCACCACTC
POX02490-R	GTAGCATCGTCAGGGCACA
POX01166-F	CGATACTACGGCAACATCATCAC
POX01166-R	AGGCACCAGTCCACGAGTTT
POX07535-F	CGACTACTTGACCCAGCACCA
POX07535-R	CTAGTACACGCTCGCAGACCA
POX06983-F	GATCAACCACCAGGGTCTCAA
POX06983-R	CAAACAACAGCCACGGAGTAAG
POX06147-F	CACAATTACGCTCGCTGGAA
POX06147-F	GGCTCGTTCATCACACCAAA
POX01896-F	GGGCAAGGATACTCGGGAAG
POX01896-R	GGCAGTTGTTGTTGACGATGTT
POX02740-F	GTTCAGTTCCTGATGGAAAGATTG
POX02740-R	CATAACCGCCTGCTTGAGTG
POX04137-F	CGGCACTCTCGGCAAGGATTA

POX04137-R	CATCAGGAAGGGGACACGGAA
POX05571-F	AACCTGGAAGAACGGCACC
POX05571-R	CCTTGTCACAGTCATCGGAGC
POX06835-F	GTGCTGGATGGGAACAGGA
POX06835-R	TACGAACGCCGAGAGGAGA
POX07963-F	ATCTTTTATGTGCTCCTACAACCAG
POX07963-R	GCCAGTCACTCATCACGAACC
POX08882-F	TCCAGGAAGCCGAGAAGAACC
POX08882-R	AGCACCGATGACACGAACGC
POX08484-F	ACAAGCACACGCAGGTCAA
POX08484-R	CGCTGAAGTGGTTGGCAGT
POX06783-F	TGAGCCCAGGACCATCAACTT
POX06783-R	TACCCTTGCTTTTGCCGCC
POX00063-F	TACCTCCCCATCGCCTTT
POX00063-R	CTTGGCACCGTAGGACTGAAC
POX05916-F	ATCGAGAATCAGGGCACAAAG
POX05916-R	ATCCGCCAACGAAGGTGT
POX02484-F	GCCCTTTTATGTCCCCTTACGA
POX02484-R	CAAACCATCCTTCATCACCAAA
POX08522-F	CGGATCATGCTCACAGTACCTT
POX08522-R	CAGGGGATGAGTGTTGCTTTC
PoxClrB-F	CTTCCAGGCGTCTCTCGTTC
PoxClrB-R	CGCTTGCTGGCTTCGTAAA
**Primers used for amplifying the probes for Southern hybridization**	
PoxClrB-F	TGACGGGAGTGGATGGGC

PoxClrB-R	AACCGGATGCAAAGGATG
POX02484-F	ATTTCTACAGTTTTGAGCGATCG
POX02484-R	GATAGCGAAACAGTAAAGGCG
POX07291-F	CCGAACGCCAAAGGAAAG
POX07291-R	GGTCAGGACAAAAGAACGATG
POX08522-F	GCAGGCAACCCTTCTAAC
POX08522-R	CGGCAGGGTCTGTATTTG
